# Development and Characterization of a Fluorinated MS-Cleavable Cross-Linker for Structural Proteomics

**DOI:** 10.1021/jasms.4c00489

**Published:** 2025-06-04

**Authors:** Oleksandr Sorokin, Frank Hause, Christian H. Ihling, Tomáš Vranka, Václav Matoušek, Andrea Sinz

**Affiliations:** † Department of Pharmaceutical Chemistry and Bioanalytics, 9176 Martin Luther University Halle-Wittenberg, 06120 Halle (Saale), Germany; ‡ Center for Structural Mass Spectrometry, Martin Luther University Halle-Wittenberg, 06120 Halle (Saale), Germany; § Institute of Molecular Medicine, Section for Molecular Cell Biology, Faculty of Medicine, Martin Luther University Halle-Wittenberg, 06120 Halle (Saale), Germany; ∥ CF Plus Chemicals s.r.o., Brno-Řečkovice 62100, Czechia

**Keywords:** cross-linking mass spectrometry, XL-MS, structural proteomics, DPFU, protein-protein interactions

## Abstract

Cross-linking mass spectrometry (XL-MS) is an important method for studying three-dimensional protein structures and mapping protein–protein interactions. Some limitations of XL-MS still consist of its use for in-cell and in vivo applications. To date, cross-linking reagents are urgently needed that can efficiently penetrate the cell membrane to comprehensively map protein–protein interaction networks in intact cells. In this study, the fluorinated MS-cleavable cross-linker bis­(pentafluorophenyl) ureido-4,4′-dibutyrate (DPFU) is described. DPFU is based on the MS-cleavable cross-linker disuccinimidyl dibutyric urea (DSBU) with the aim of balancing the hydrophobicity and solubility to improve membrane permeability. DPFU was evaluated for its solubility behavior in different detergent solutions to optimize conditions for its potential application in live cells. Using bovine serum albumin (BSA) as a model protein, XL-MS experiments were conducted across different temperatures and cross-linker concentrations. Solubility assays identified sodium dodecyl sulfate (SDS) as effective for enhancing DPFU solubility in an aqueous environment. DPFU yielded fewer cross-links for BSA than DSBU, highlighting limitations regarding its cross-linking efficiency under similar experimental conditions. This study provides the first insights into fluorinated cross-linkers, suggesting that further optimization is needed for a broader application of DPFU for future in-cell XL-MS studies.

## Graphical abstract

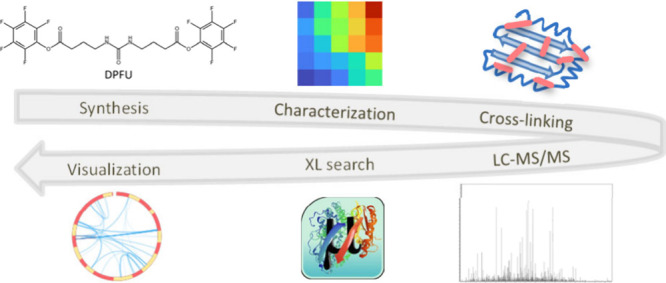

## Introduction

For more than 20 years, cross-linking mass spectrometry (XL-MS) has emerged as a powerful technique for exploring the 3D-structures of proteins and protein complexes in their native environment.^1–6^ Chemical cross-linkers covalently bridge two amino acids in spatial proximity, and therefore serve as “molecular rulers” to study the topology and plasticity of proteins and protein complexes. The complexity of cross-linked samples is one of the challenges for MS data analysis in XL-MS. MS-cleavable cross-linkers, such as disuccinimidyl dibutyric urea (DSBU),^7,8^ disuccinimidylsulfoxide (DSSO),^9^ as well as the trifunctional MS-cleavable and enrichable protein interaction reporter (PIR)^10^ and disuccinimidyl disuccinic imide (DSSI)^11^ offer valuable solutions for reducing sample complexity. By collision-induced dissociation of the cross-linker, the individual masses of cross-linked peptides are obtained. This reduces the software search space and improves sequencing of the connected peptides.^12–15^

One of the major advantages of XL-MS is its capability to study proteins and protein–protein interactions in intact cells, in tissues, and in organisms.^16,17^ For XL-MS studies of intact cells, cross-linkers that can efficiently permeate the cell membrane are needed. The MS-cleavable *N*-hydroxysuccinimide (NHS) ester DSBU can enter cells, but our aim was to improve cell membrane permeability by substituting the NHS esters with pentafluorinated phenyl groups (Chart 1). Therefore, the DPFU linker containing fluorinated phenyl residues is expected to possess a higher membrane permeability compared to that of DSBU due to its higher lipophilicity.

**Chart 1:**
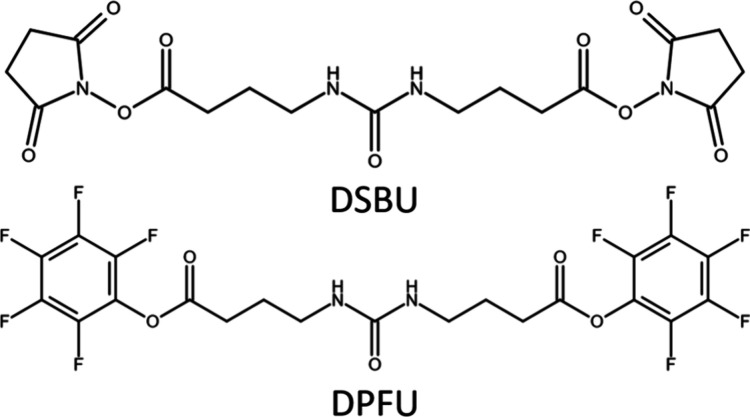
Chemical Structures of Disuccinimidyl Dibutyric Urea (DSBU) and Bis­(pentafluorophenyl) Ureido-4,4′-dibutyrate (DPFU)

## Experimental Section

### Materials

DSBU was obtained from CF Plus Chemicals, while BSA (bovine serum albumin) and other reagents were obtained from Sigma-Aldrich. The synthesis of DPFU (bis­(pentafluorophenyl) ureido-4,4′-dibutyrate) is provided in the Supporting Information, Figures S1–S4. All solvents were of LC/MS grade and purchased from VWR.

### DPFU Solubility Assays

DPFU was dissolved in DMSO to a stock concentration of 50 mM prior to each experiment. The detergents sodium dodecylsufate (SDS), desoxycholate (DOC), and dodecyl maltoside (DDM) were solubilized in 50 mM HEPES buffer (pH 7.5) to create 4 mM stock solutions.

All samples were prepared in a 96-well plate (Greiner Bio-One) with a final volume of 100 μL per well. DPFU was applied at final concentrations ranging from 0 to 1 mM in 5% (v/v) DMSO. 95 μL of detergent solution (50 mM HEPES, pH 7.5) was added to yield detergent concentrations ranging from 0 to 2 mM. Samples were incubated for 15 min at room temperature before measuring OD values at 600 nm indicating DPFU aggregation (Supporting Information, Tables S1–S3). Heatmaps representing detergent versus DPFU concentrations were generated (Supporting Information, Figure S5).

### XL-MS Experiments

BSA was solubilized in 50 mM HEPES buffer (pH 7.5) to a final concentration of 10 μM. Cross-linking reactions were performed at 4 and 20 °C for 30, 90, and 120 min with a 20-, 50-, and 100-fold molar excess of DPFU. Successful cross-linking of BSA was confirmed by SDS-PAGE analysis (Supporting Information, Figure S6). The DMSO concentration in each sample was adjusted to 5% using pure DMSO. Cross-linking reactions were quenched by adding hydroxyl-amine to a final concentration of 2.5% (v/v). For comparison, cross-linking reactions were also conducted with DSBU.

After cross-linking, samples were lyophilized, and the pellet was dissolved in 25 μL of 8 M urea in a 400 mM ammonium bicarbonate solution. Proteolysis of cross-linked samples was performed acording to an existing protocol.^18^

Tryptic digestion of the reaction mixtures was carried out with Mass Spectrometry grade Trypsin Gold (Promega) at 37 °C overnight. After digestion, samples were analyzed by LC-MS/MS (Ultimate RSLC nano-HPLC, Thermo Fisher Scientific, coupled to a timsTOF Pro mass spectrometer, Bruker Daltonik) using an established protocol.^19^

### XL-MS Data Analysis

Cross-linking sites were identified with the MeroX 2.0.1.7 software^20^ using established settings^18^ at a false discovery rate (FDR) of 1%. Cross-links with MeroX scores below 50 were excluded. Heatmaps and Circos plots were generated using Matplotlib and XLDataGraph (https://github.com/a-helix/XLDataGraph) Python3 libraries.

## Results and Discussion

### Solubility of DPFU in Aqueous Solutions

Due to its structure, DPFU has a relatively low water solubility. To address this limitation, three detergents (SDS, DOC, and DDM) were evaluated for their ability to enhance DPFU solubility. Key criteria for detergent selection included minimal protein-denaturing properties and compatibility with LC-MS/MS analysis. SDS is known to be applicable for protein structure analysis at concentrations between 0.1 and 2.0 mM.^21,22^

The solubility assay indicated that DOC and DDM had minimal impact on DPFU solubility in aqueous solution (Supporting Information and Figure S5). In contrast, SDS improved DPFU solubility at concentrations exceeding 0.75 mM, with no additional improvement above 1 mM SDS (Figure 1). Therefore, this concentration was selected for XL-MS experiments as optimal concentration to enhance DPFU solubility, while minimizing potential SDS-induced denaturing effects on BSA.

**Figure 1:**
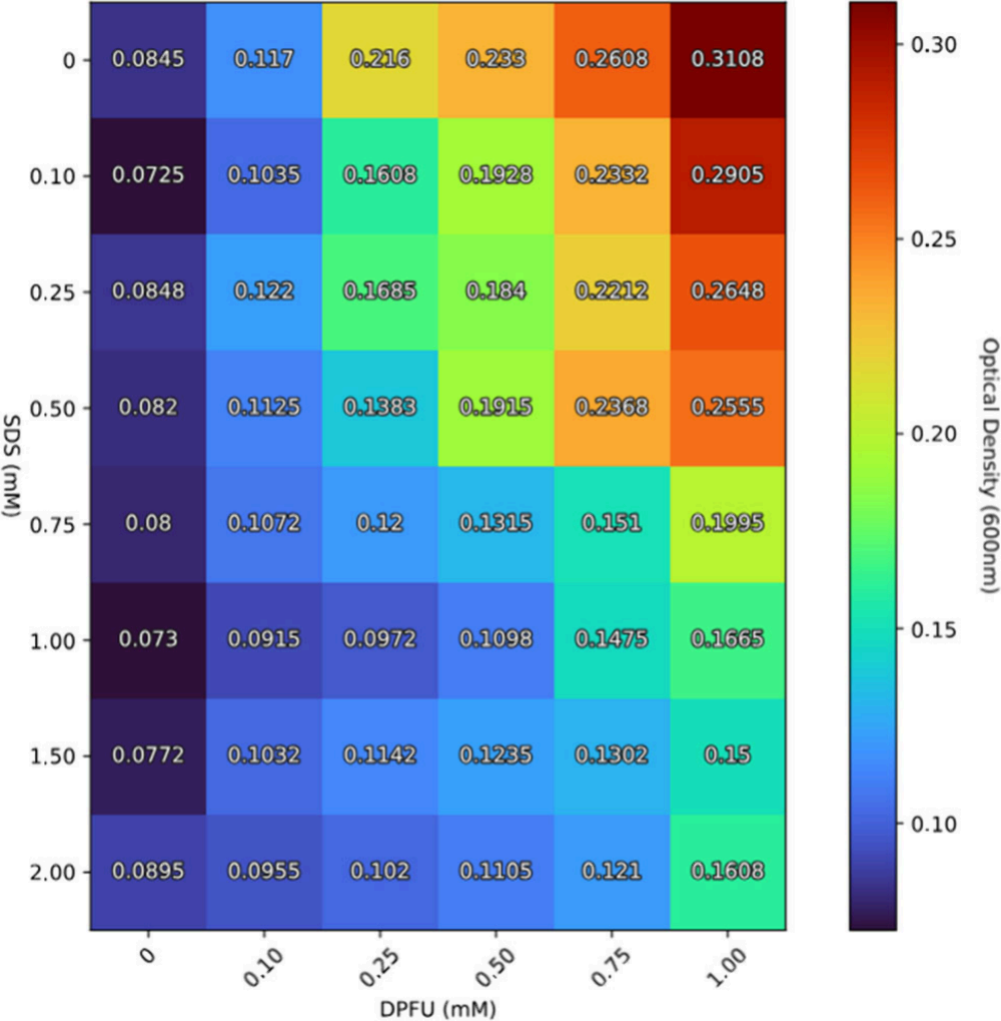
Assay for examining the solubility-enhancing properties of sodium dodecyl sulfate (SDS) in an aqueous solution of DPFU (bis­(pentafluorophenyl) ureido-4,4′-dibutyrate).

The highest numbers of cross-links with DPFU were identified at 90 min reaction time, 20 °C, and a 50-fold molar excess of cross-linker (Supporting Information and Table S4). Exemplary fragment ion mass spectra, automatically assigned by MeroX, are presented in the Supporting Information and Figures S7–S21. The cross-links were also found for the reference cross-linker DSBU, but the total number was lower due to the presence of SDS. All cross-links are in agreement with the 3D structure of BSA (Supporting Information, Figures S22–S66). This underlines the general applicability of the DPFU cross-linker to structural proteomics.

As for DSBU, DPFU contains a central urea group (Figure 1) that is cleavable upon collisional activation. Consequently, the two characteristic doublets are visible in the fragment ion mass spectra due to the fragmentation of the C=O–NH bond at the central urea moiety of DPFU (Figure 2).

**Figure 2:**
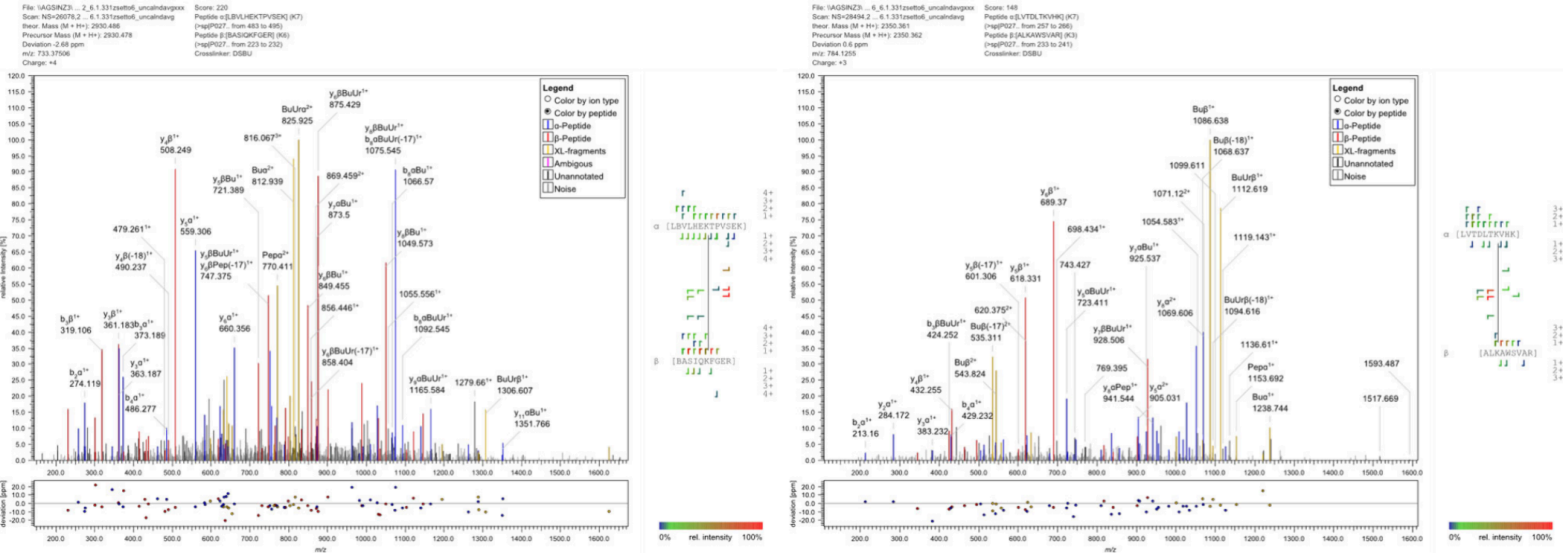
Two exemplary fragment ion mass spectra for DPFU cross-links analyzed by MeroX; b- and y-type ions are shown in blue and red*;* fragment ions of the cross-linker are shown in yellow. B (in the amino acid sequence) indicates carbamidomethylated Cys.

The DPFU linker exhibits identical fragmentation behavior upon collisional activation as the established DSBU linker and therefore offers identical possibilities as DSBU for the facilitated identification of cross-links from complex mixtures.

## Conclusion

In this study, we describe the first fluorinated MS-cleavable cross-linker DPFU as a potential tool for advancing XL-MS applications. While DPFU demonstrates improved solubility in an aqueous environment in the presence of SDS, its cross-linking efficiency with BSA under the conditions tested was lower compared to DSBU. Our original idea was to increase the diffusion rate of the cross-linker through the cell membrane by incorporating a leaving group of similar potency to N-hydroxysuccinimide but increased lipophilicity. Clearly, the overall cross-linking performance is the combined result of solubility, membrane pentration rate, and chemical reactivity. The application of this fluorinated cross-linker for in-cell XL-MS is a challenge in future studies.

## Supplementary Material



## Notes

Synthesis of DPFU, OD_600_ values of DPFU solubility in the presence of detergents, gradient heatmaps of precipitation intensities for DPFU in the presence of detergents, SDS-PAGE analysis of cross-linked BSA, distribution of unique DSBU and DPFU cross-links in BSA, MeroX analysis of selected fragment ion mass spectra of cross-linked peptides, Circos plots of DSBU and DPFU cross-links in BSA, and 3D models of DSBU and DPFU cross-links in BSA (PDF)
