# Supplementary material for: Development and Characterization of a Fluorinated MS-Cleavable Cross-Linker for Structural Proteomics
Source: J Am Soc Mass Spectrom. 2025 Jun 4;36(6):1410–3. doi: 10.1021/jasms.4c00489 (PMC12142660; doi:10.1021/jasms.4c00489)
Supplement: Supplementary file 1 [file js4c00489_si_001.pdf]

Supporting Information

Development and Characterization of a Fluorinated MS-Cleavable Cross-Linker for Structural Proteomics

Oleksandr Sorokin[1,2], Frank Hause[1,2,3], Christian H. Ihling[1,2], Tomáš Vranka[4], Václav Matoušek[4], Andrea Sinz[*,1,2]

[1] Department of Pharmaceutical Chemistry and Bioanalytics, Martin Luther University Halle-Wittenberg, 06120 Halle (Saale), Germany

[2] Center for Structural Mass Spectrometry, Martin Luther University Halle-Wittenberg, 06120 Halle (Saale), Germany

[3] Institute of Molecular Medicine, Section for Molecular Cell Biology, Faculty of Medicine, Martin Luther University Halle-Wittenberg, 06120 Halle (Saale), Germany

[4] CF Plus Chemicals s.r.o., Brno-Řečkovice 62100, Czechia

*to whom correspondence should be addressed – andrea.sinz@pharmazie.uni-halle.de

**Table of Contents**	**S2**
Synthesis of bis(pentafluorophenyl) ureido-4,4´-dibutyrate (DPFU)	S3–6
OD_{600} values of DPFU solubility in the presence of detergents	S7-9
Gradient heatmaps of precipitation intensities for DPFU in the presence of detergents	S10
SDS-PAGE analysis of cross-linked BSA	S11
Distribution of unique DSBU and DPFU cross-links in BSA	S12
MeroX analysis of selected fragment ion mass spectra of cross-linked peptides	S13-27
Circos plots of DSBU and DPFU cross-links in BSA	S28-63
3D models of DSBU and DPFU cross-links in BSA	S64-72

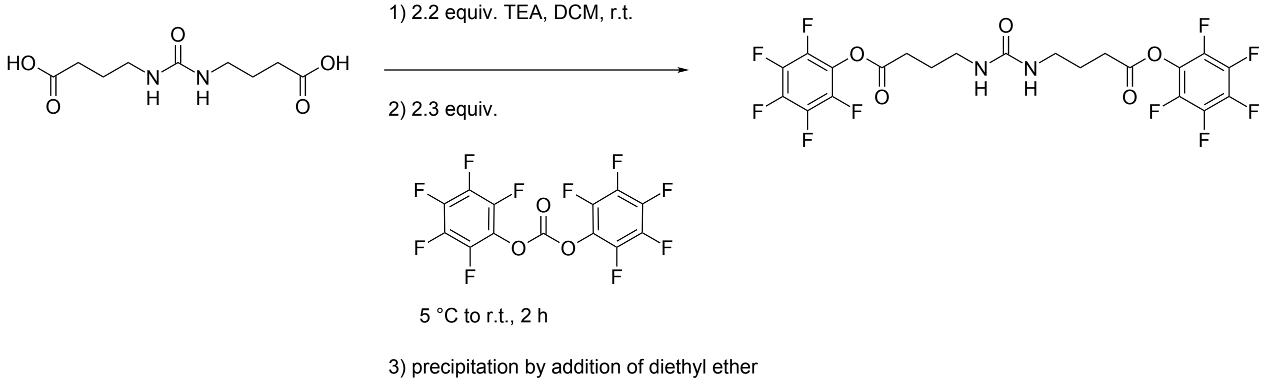

Figure S1. Synthesis of bis(pentafluorophenyl) ureido-4,4′-dibutyrate (DPFU). Ureido-4,4′-dibutanoic acid (1 g, 4.305 mmol, 1 equiv.) was suspended in 10 ml anhydrous dichloromethane (DCM) at room temperature under argon. Triethylamine (0.96 g, 1.32 ml, 9.47 mmol, 2.2 equiv.) was added dropwise (1 min) to the suspension. The resulting mixture was sonicated for 3 min to obtain a solution, indicating the formation of the DCM-soluble bis-triethylammonium salt. The solution was cooled to 4 °C and bis(pentafluorophenyl)carbonate (3.9 g, 9.9 mmol, 2.3 equiv.) was added. The mixture was stirred for 2 h at room temperature. Afterwards, the reaction mixture was cooled to -20 °C and diethyl ether (30 ml) was added dropwise (2 min) to induce precipitation of the product. The suspension was stirred at -20 °C for 5 min and the solid compound was washed with cold diethyl ether (20 ml, -20 °C). DPFU (1.28 g, 53%) was obtained as a colorless solid and dried under high vacuum. Identity of the compound was confirmed by NMR (Figures S2-S4).

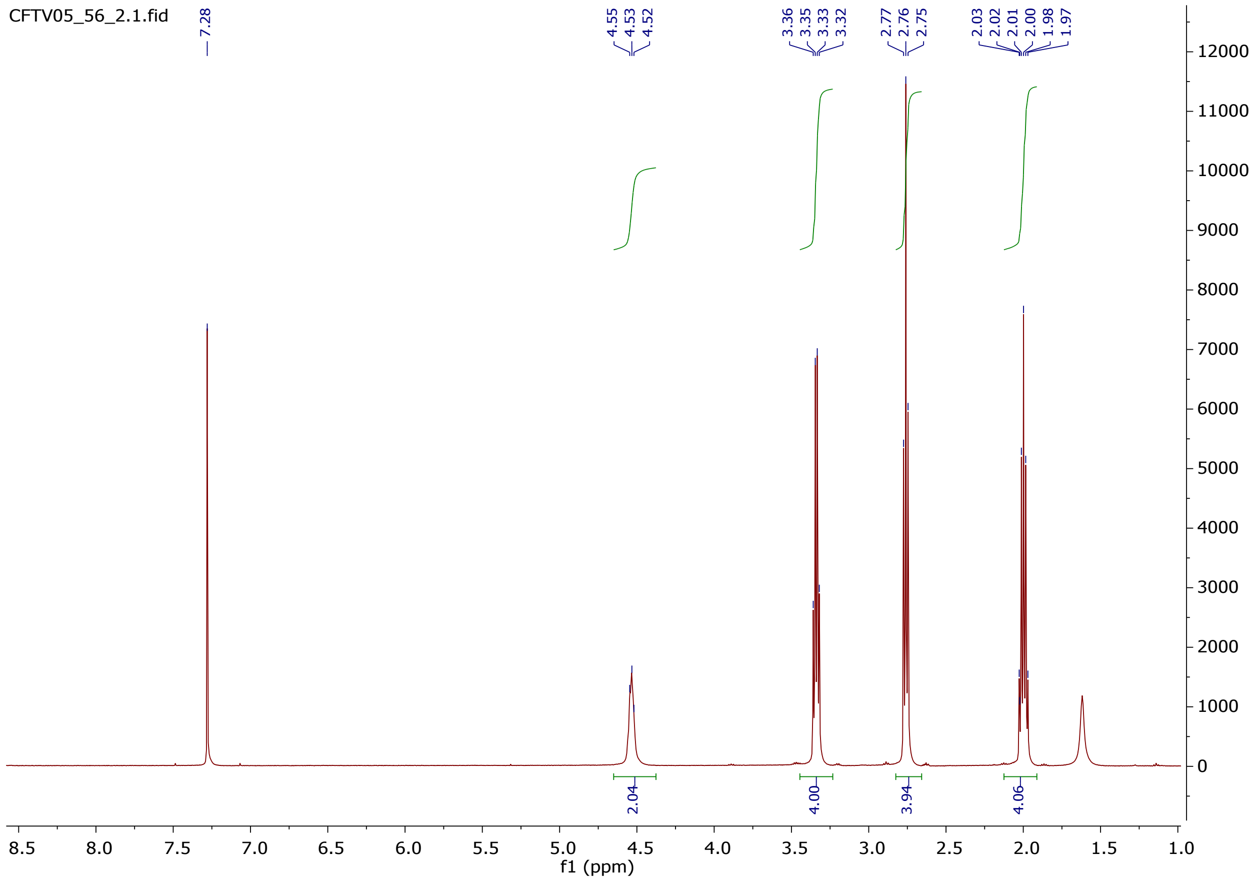

Figure S2. ^{1}H NMR (500 MHz, $CDCl_3$, 25 °C) δ 1.98 (ap p, J = 7.0 Hz, 4H), 2.74 (t, J = 7.2 Hz, 4H), 3.32 (q, J = 6.6 Hz, 4H), 4.52 (d, J = 7.0 Hz, 2H).

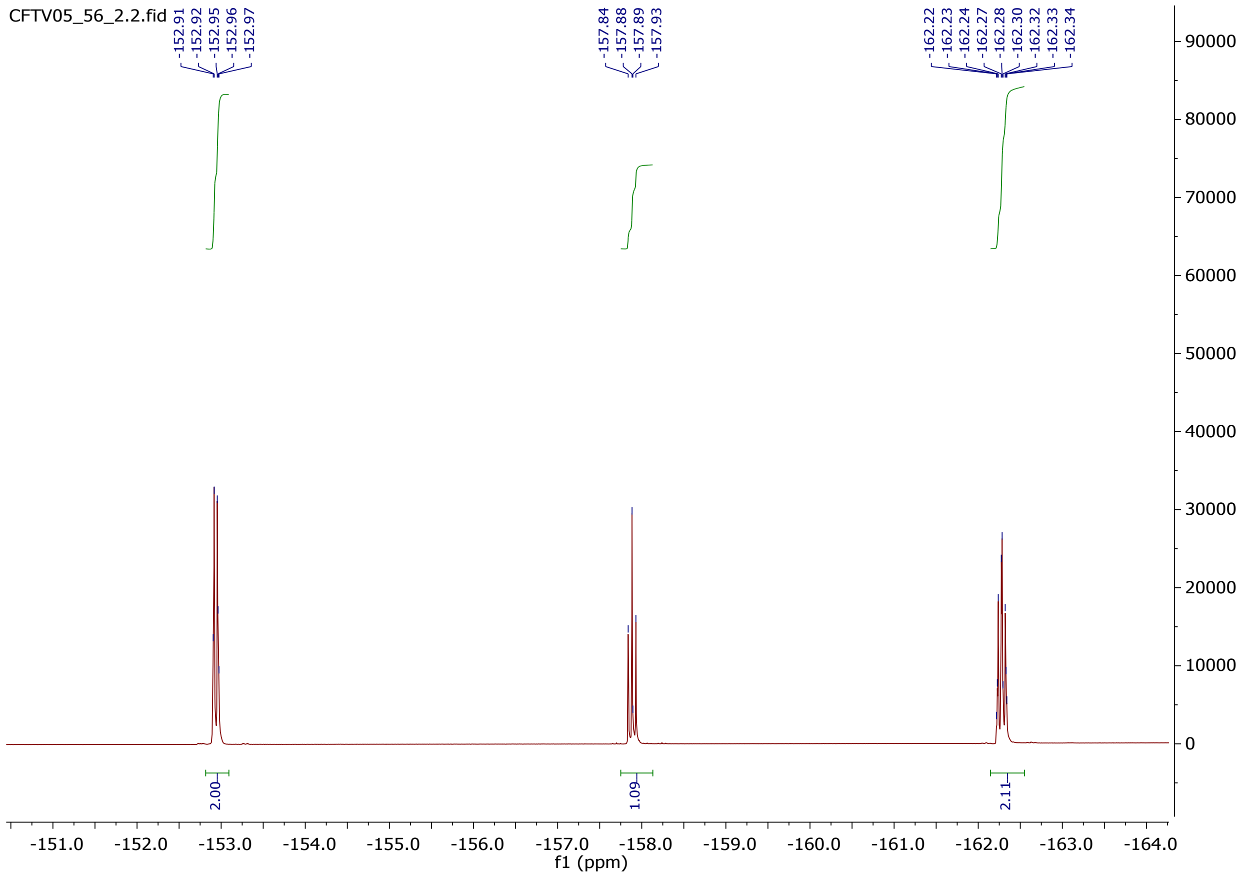

Figure S3. ^{19}F NMR (471 MHz, $CDCl_3$, 25 °C) δ -152.94 (m, 4F), -157.88 (t, *J* = 21.6 Hz, 2F), -162.28 (m, 4F).

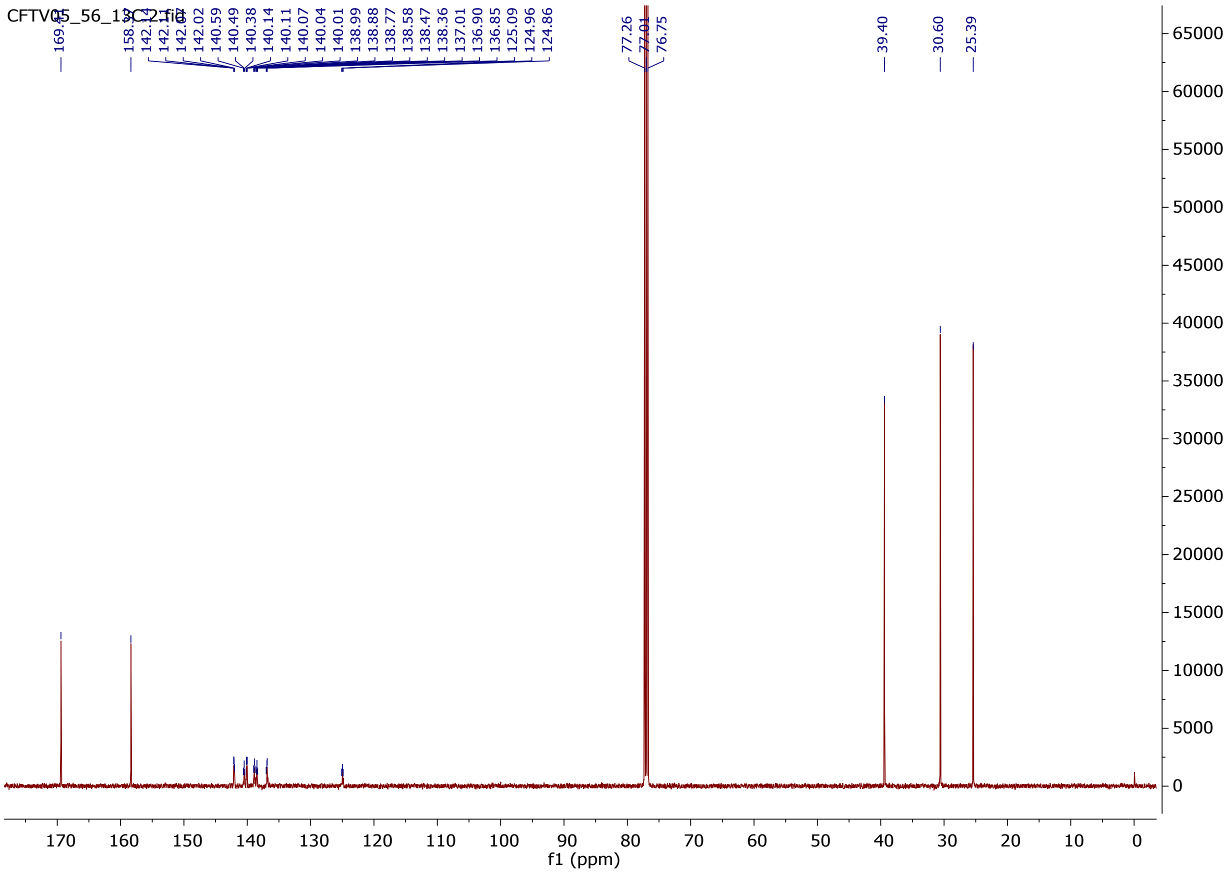

Figure S4. ^{13}C NMR (126 MHz, $CDCl_3$, 25 °C) δ 169.41 (s), 158.37 (s), 141.09 (dm, J = 252.4 Hz), 139.45 (dm, J = 254.3 Hz), 137.80 (dm, J = 255.3 Hz), 125.03 (m), 39.40, 30.60, 25.39

Table S1. OD_{600} values of DPFU solubility in the presence of DOC. DPFU concentrations ranged between 0 to 2 mM (rows A to H), DOC concentrations ranged between 0 to 1 mM (columns 1 to 6 and 7 to 12). Samples were measured in four independent replicates. MV (mean values) and SD (standard deviations) are presented.

OD_{600}	1	2	3	4	5	6	7	8	9	10	11	12
A	0.08	0.097	0.121	0.174	0.209	0.301	0.082	0.116	0.135	0.144	0.186	0.278
B	0.081	0.106	0.11	0.134	0.187	0.249	0.072	0.099	0.106	0.139	0.22	0.259
C	0.076	0.084	0.111	0.173	0.206	0.224	0.074	0.101	0.108	0.133	0.191	0.27
D	0.077	0.099	0.098	0.136	0.189	0.214	0.078	0.088	0.101	0.165	0.172	0.233
E	0.078	0.085	0.101	0.142	0.158	0.188	0.078	0.092	0.109	0.145	0.179	0.255
F	0.078	0.086	0.099	0.139	0.161	0.217	0.072	0.087	0.108	0.141	0.171	0.212
G	0.076	0.087	0.1	0.134	0.169	0.207	0.078	0.105	0.116	0.144	0.185	0.222
H	0.093	0.091	0.097	0.143	0.177	0.217	0.086	0.102	0.106	0.191	0.168	0.274

OD_{600}	1	2	3	4	5	6	7	8	9	10	11	12
A	0.08	0.103	0.112	0.163	0.179	0.236	0.086	0.155	0.128	0.138	0.269	0.261
B	0.068	0.09	0.1	0.142	0.183	0.245	0.074	0.093	0.101	0.133	0.19	0.241
C	0.077	0.082	0.103	0.196	0.163	0.21	0.073	0.094	0.103	0.132	0.188	0.309
D	0.075	0.09	0.102	0.135	0.162	0.212	0.075	0.091	0.1	0.13	0.182	0.223
E	0.071	0.086	0.1	0.139	0.163	0.207	0.072	0.087	0.098	0.135	0.158	0.207
F	0.07	0.084	0.101	0.147	0.175	0.208	0.074	0.096	0.101	0.137	0.167	0.222
G	0.076	0.083	0.103	0.16	0.166	0.221	0.099	0.093	0.112	0.145	0.247	0.208
H	0.098	0.08	0.102	0.126	0.172	0.215	0.086	0.097	0.108	0.129	0.169	0.183

MV	1	2	3	4	5	6
A	0.082	0.118	0.124	0.155	0.211	0.269
B	0.074	0.097	0.104	0.137	0.195	0.249
C	0.075	0.090	0.106	0.159	0.187	0.253
D	0.076	0.092	0.100	0.142	0.176	0.221
E	0.075	0.088	0.102	0.140	0.165	0.214
F	0.074	0.088	0.102	0.141	0.169	0.215
G	0.082	0.092	0.108	0.146	0.192	0.215
H	0.091	0.093	0.103	0.147	0.172	0.222

SD	1	2	3	4	5	6
A	0.003	0.026	0.010	0.017	0.041	0.027
B	0.005	0.007	0.005	0.004	0.017	0.008
C	0.002	0.009	0.004	0.031	0.018	0.045
D	0.002	0.005	0.002	0.016	0.012	0.010
E	0.004	0.003	0.005	0.004	0.010	0.029
F	0.003	0.005	0.004	0.004	0.006	0.006
G	0.011	0.010	0.008	0.011	0.038	0.008
H	0.006	0.009	0.005	0.030	0.004	0.038

Table S2. OD_{600} values of DPFU solubility in the presence of DDM. DPFU concentrations ranged between 0 to 2 mM (rows A to H), DDM concentrations ranged between 0 to 1 mM (columns 1 to 6 and 7 to 12). Samples were measured in four independent replicates. MV (mean values) and SD (standard deviations) are presented.

OD_{600}	1	2	3	4	5	6	7	8	9	10	11	12
A	0.084	0.109	0.198	0.273	0.292	0.354	0.075	0.116	0.232	0.323	0.353	0.359
B	0.08	0.093	0.118	0.158	0.249	0.32	0.072	0.13	0.146	0.188	0.256	0.299
C	0.075	0.114	0.13	0.202	0.208	0.251	0.07	0.147	0.154	0.204	0.235	0.299
D	0.07	0.105	0.131	0.207	0.234	0.268	0.077	0.099	0.141	0.2	0.222	0.268
E	0.076	0.105	0.141	0.237	0.289	0.318	0.084	0.151	0.163	0.174	0.221	0.272
F	0.073	0.101	0.126	0.225	0.304	0.321	0.071	0.123	0.132	0.175	0.21	0.237
G	0.078	0.103	0.118	0.189	0.255	0.316	0.072	0.131	0.131	0.188	0.204	0.29
H	0.096	0.115	0.133	0.218	0.345	0.39	0.085	0.155	0.214	0.25	0.27	0.349
**OD_{600}**	**1**	**2**	**3**	**4**	**5**	**6**	**7**	**8**	**9**	**10**	**11**	**12**
A	0.093	0.091	0.117	0.118	0.258	0.328	0.087	0.092	0.119	0.159	0.217	0.323
B	0.073	0.098	0.131	0.224	0.278	0.341	0.073	0.114	0.124	0.211	0.259	0.343
C	0.079	0.089	0.098	0.134	0.214	0.283	0.074	0.088	0.105	0.134	0.207	0.266
D	0.088	0.095	0.101	0.112	0.252	0.272	0.08	0.098	0.11	0.205	0.238	0.294
E	0.077	0.136	0.105	0.164	0.26	0.269	0.087	0.105	0.105	0.141	0.22	0.254
F	0.074	0.151	0.139	0.185	0.274	0.317	0.073	0.121	0.13	0.206	0.296	0.337
G	0.073	0.115	0.143	0.185	0.296	0.37	0.071	0.115	0.13	0.213	0.319	0.328
H	0.083	0.095	0.111	0.164	0.342	0.368	0.087	0.094	0.178	0.188	0.204	0.247

MV	1	2	3	4	5	6
A	0.085	0.102	0.167	0.218	0.280	0.341
B	0.075	0.109	0.130	0.195	0.261	0.326
C	0.075	0.110	0.122	0.169	0.216	0.275
D	0.079	0.099	0.121	0.181	0.237	0.276
E	0.081	0.124	0.129	0.179	0.248	0.278
F	0.073	0.124	0.132	0.198	0.271	0.303
G	0.074	0.116	0.131	0.194	0.269	0.326
H	0.088	0.115	0.159	0.205	0.290	0.339
**SD**	**1**	**2**	**3**	**4**	**5**	**6**
A	0.008	0.012	0.058	0.096	0.058	0.018
B	0.004	0.017	0.012	0.029	0.012	0.021
C	0.004	0.028	0.026	0.040	0.013	0.021
D	0.007	0.004	0.018	0.046	0.012	0.012
E	0.005	0.023	0.029	0.041	0.033	0.028
F	0.001	0.021	0.005	0.022	0.043	0.045
G	0.003	0.011	0.010	0.013	0.050	0.033
H	0.006	0.029	0.046	0.037	0.067	0.063

Table S3. OD_{600} values of DPFU solubility in the presence of SDS. DPFU concentrations ranged between 0 to 2 mM (rows A to H), SDS concentrations ranged between 0 to 1 mM (columns 1 to 6 and 7 to 12). Samples were measured in four independent replicates. MV (mean values) and SD (standard deviations) are presented.

OD_{600}	1	2	3	4	5	6	7	8	9	10	11	12
A	0.087	0.152	0.179	0.257	0.302	0.315	0.09	0.124	0.269	0.298	0.299	0.353
B	0.072	0.115	0.189	0.257	0.268	0.304	0.072	0.096	0.175	0.186	0.266	0.32
C	0.121	0.148	0.219	0.234	0.264	0.291	0.068	0.171	0.248	0.25	0.268	0.292
D	0.091	0.121	0.142	0.174	0.283	0.267	0.081	0.148	0.156	0.249	0.27	0.313
E	0.094	0.087	0.118	0.135	0.136	0.197	0.083	0.121	0.125	0.128	0.168	0.171
F	0.07	0.092	0.098	0.099	0.111	0.131	0.081	0.099	0.111	0.121	0.144	0.197
G	0.076	0.091	0.11	0.115	0.117	0.138	0.075	0.107	0.113	0.124	0.137	0.181
H	0.085	0.087	0.095	0.099	0.114	0.174	0.092	0.102	0.105	0.115	0.13	0.162
OD_{600}	1	2	3	4	5	6	7	8	9	10	11	12
A	0.078	0.109	0.251	0.172	0.196	0.328	0.083	0.083	0.165	0.205	0.246	0.247
B	0.073	0.106	0.14	0.165	0.183	0.299	0.073	0.097	0.139	0.163	0.216	0.239
C	0.073	0.091	0.099	0.137	0.191	0.241	0.077	0.078	0.108	0.115	0.162	0.235
D	0.085	0.093	0.117	0.176	0.199	0.219	0.071	0.088	0.138	0.167	0.195	0.223
E	0.073	0.101	0.107	0.124	0.143	0.212	0.07	0.12	0.13	0.139	0.157	0.218
F	0.071	0.085	0.088	0.099	0.207	0.19	0.07	0.09	0.092	0.12	0.128	0.148
G	0.083	0.107	0.116	0.125	0.132	0.139	0.075	0.108	0.118	0.13	0.135	0.142
H	0.09	0.1	0.106	0.111	0.116	0.157	0.091	0.093	0.102	0.117	0.124	0.15

MV	1	2	3	4	5	6
A	0.085	0.117	0.216	0.233	0.261	0.311
B	0.073	0.104	0.161	0.193	0.233	0.291
C	0.085	0.122	0.169	0.184	0.221	0.265
D	0.082	0.113	0.138	0.192	0.237	0.256
E	0.080	0.107	0.120	0.132	0.151	0.200
F	0.073	0.092	0.097	0.110	0.148	0.167
G	0.077	0.103	0.114	0.124	0.130	0.150
H	0.090	0.096	0.102	0.111	0.121	0.161
SD	1	2	3	4	5	6
A	0.005	0.029	0.052	0.056	0.050	0.045
B	0.001	0.009	0.025	0.044	0.041	0.035
C	0.024	0.045	0.076	0.068	0.053	0.031
D	0.008	0.028	0.016	0.039	0.046	0.044
E	0.011	0.016	0.010	0.007	0.014	0.021
F	0.005	0.006	0.010	0.012	0.042	0.032
G	0.004	0.008	0.004	0.006	0.009	0.021
H	0.003	0.007	0.005	0.008	0.007	0.010

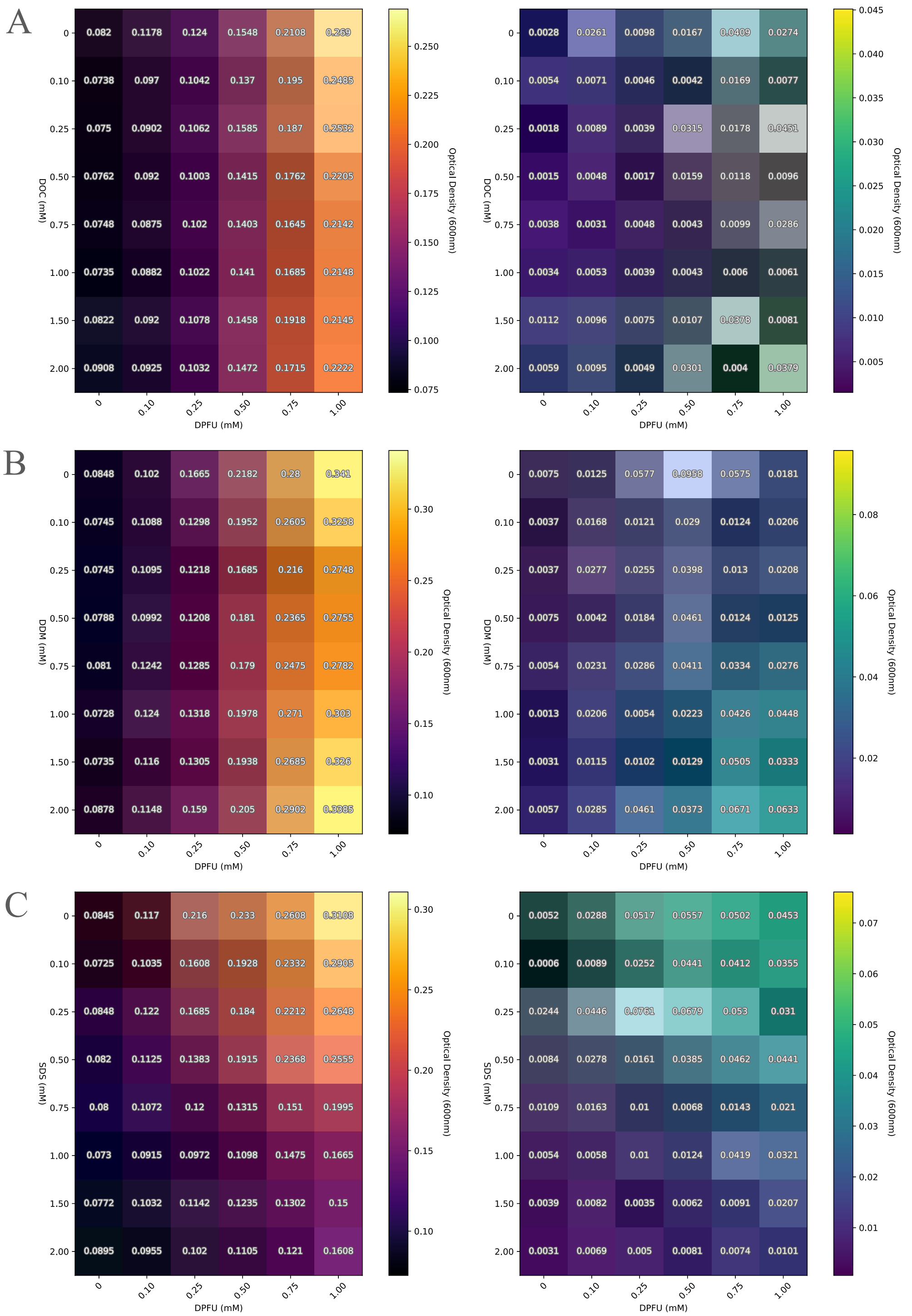

Figure S5. Gradient heatmaps showing the median values (left) and standard deviations (right) of precipitation intensities for DPFU in the presence of the detergents DOC (A), DDM (B), and SDS (C) in 50 mM HEPES (pH 7.5) buffer. All datapoints are based on triplicate measurements. Scale bars are adjusted based on minimum and maximum values of each heatmap.

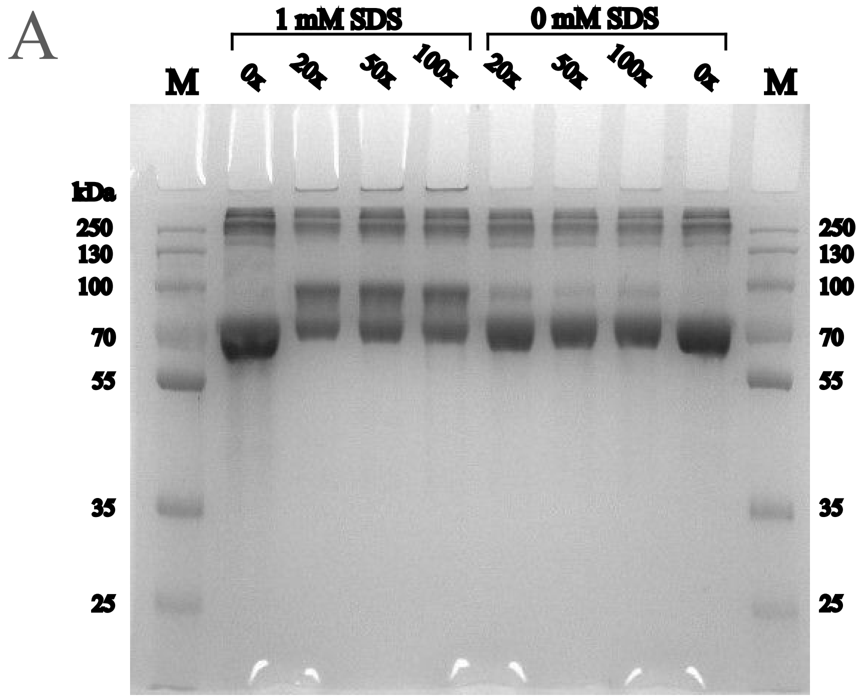

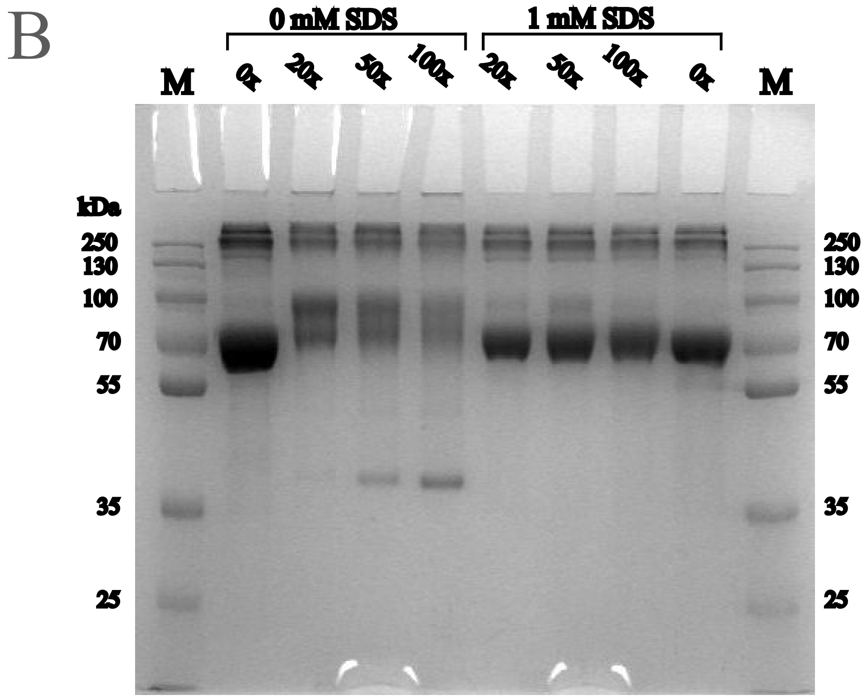

Figure S6. SDS-PAGE analysis (10% resolving gel) of BSA cross-linking in the presence of different (20-, 50-, and 100-fold) molar excess of (A) DPFU and (B) DSBU, with and without 1 mM SDS. Controls were performed without the addition of cross-linker and are marked with "0x". M – PageRuler Prestained Protein Ladder (Thermo Scientific).

Table S4. The distributions of unique DSBU and DPFU cross-links in BSA are presented across three replicates for four conditions (30 and 90 min at 20°C; 90 and 120 min at 4°C) in the presence and absence of 1 mM SDS. The numbers of unique cross-links identified in one, two or three replicates are shown.

t (min)	T (°C)	SDS (mM)	Linker	Fold Access	XLs in 1 repl.	XLs in 2 repl.	XLs in 3 repl.	Total XLs
30	20	0	DSBU	20x	85	46	139	270
				50x	81	53	126	260
				100x	79	60	136	275
30	20	1	DSBU	20x	32	14	30	76
				50x	32	16	38	86
				100x	34	25	53	112
30	20	1	DPFU	20x	13	6	21	40
				50x	15	11	17	43
				100x	13	11	16	40
90	20	0	DSBU	20x	88	56	146	290
				50x	99	86	104	289
				100x	72	60	142	274
90	20	1	DSBU	20x	24	19	42	85
				50x	36	26	52	114
				100x	56	30	60	146
90	20	1	DPFU	20x	18	19	19	56
				50x	24	10	30	64
				100x	20	20	21	61
90	4	0	DSBU	20x	69	48	105	222
				50x	81	57	118	256
				100x	116	71	36	223
90	4	1	DSBU	20x	9	2	11	22
				50x	6	10	15	31
				100x	20	19	27	66
90	4	1	DPFU	20x	0	3	7	10
				50x	3	1	8	12
				100x	5	1	8	14
120	4	0	DSBU	20x	60	46	99	205
				50x	66	51	127	244
				100x	73	59	145	277
120	4	1	DSBU	20x	6	12	16	34
				50x	13	7	26	46
				100x	15	16	31	62
120	4	1	DPFU	20x	4	3	6	13
				50x	8	1	9	18
				100x	3	2	9	14

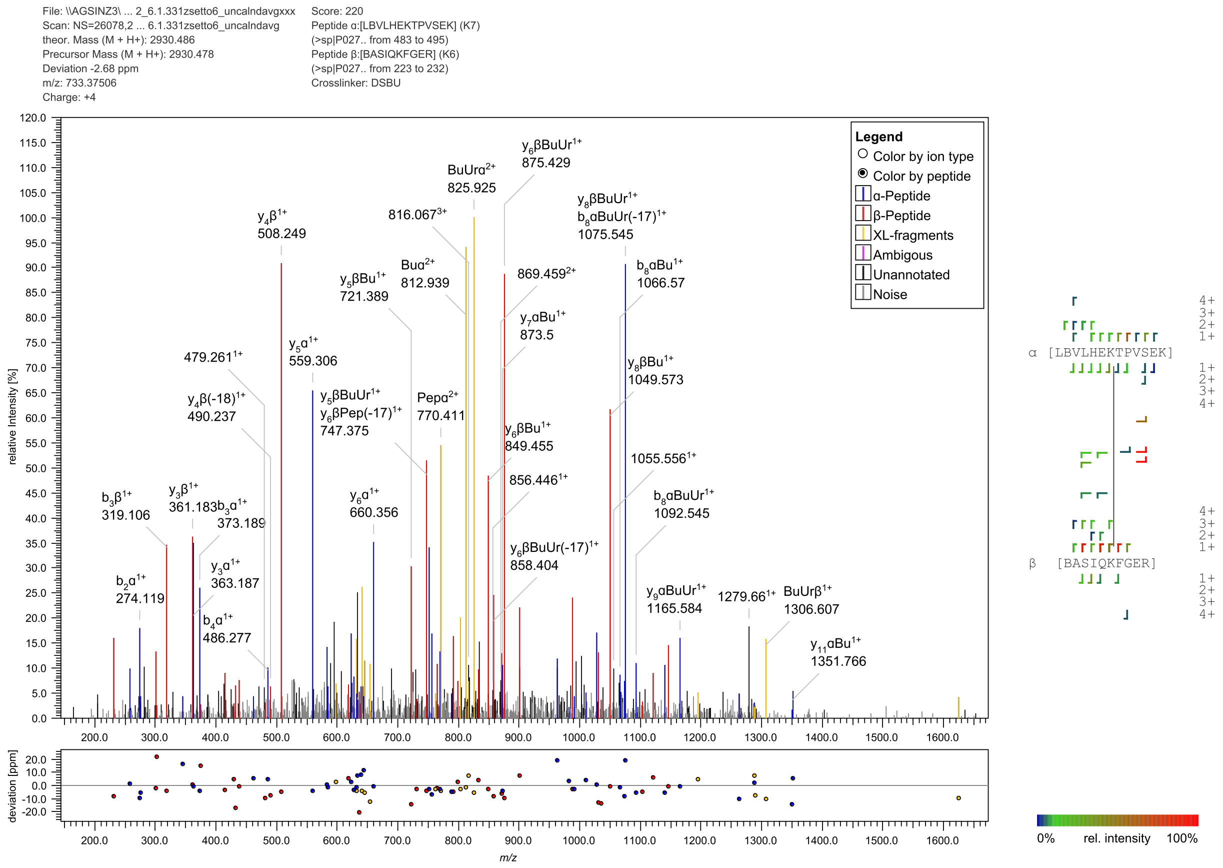

Figure S7. Fragment ion mass spectrum of DPFU cross-link analyzed by MeroX; b- and y-type ions are shown in blue and red; fragment ions of the cross-linker are shown in yellow. B in the amino acid sequence indicates carbamidomethylated Cys.

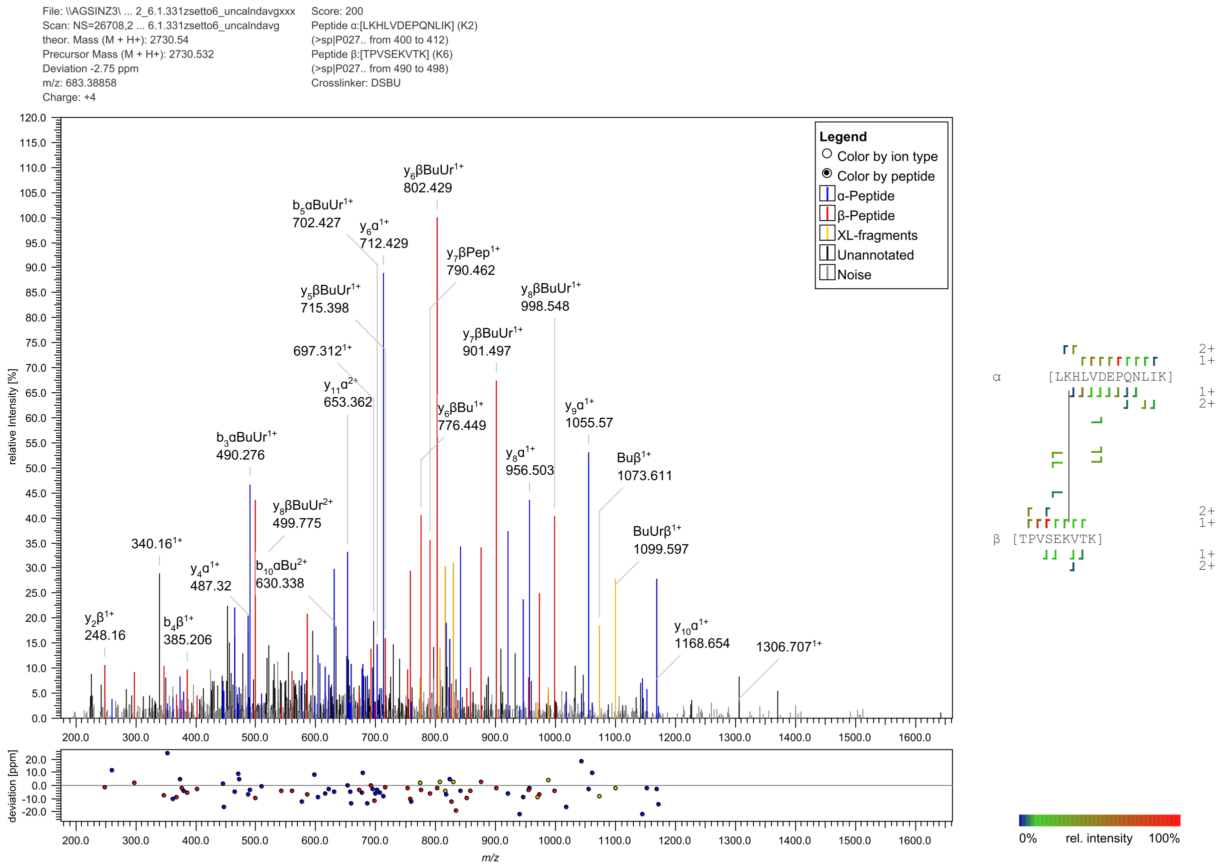

Figure S8. Fragment ion mass spectrum of DPFU cross-link analyzed by MeroX; b- and y-type ions are shown in blue and red; fragment ions of the cross-linker are shown in yellow.

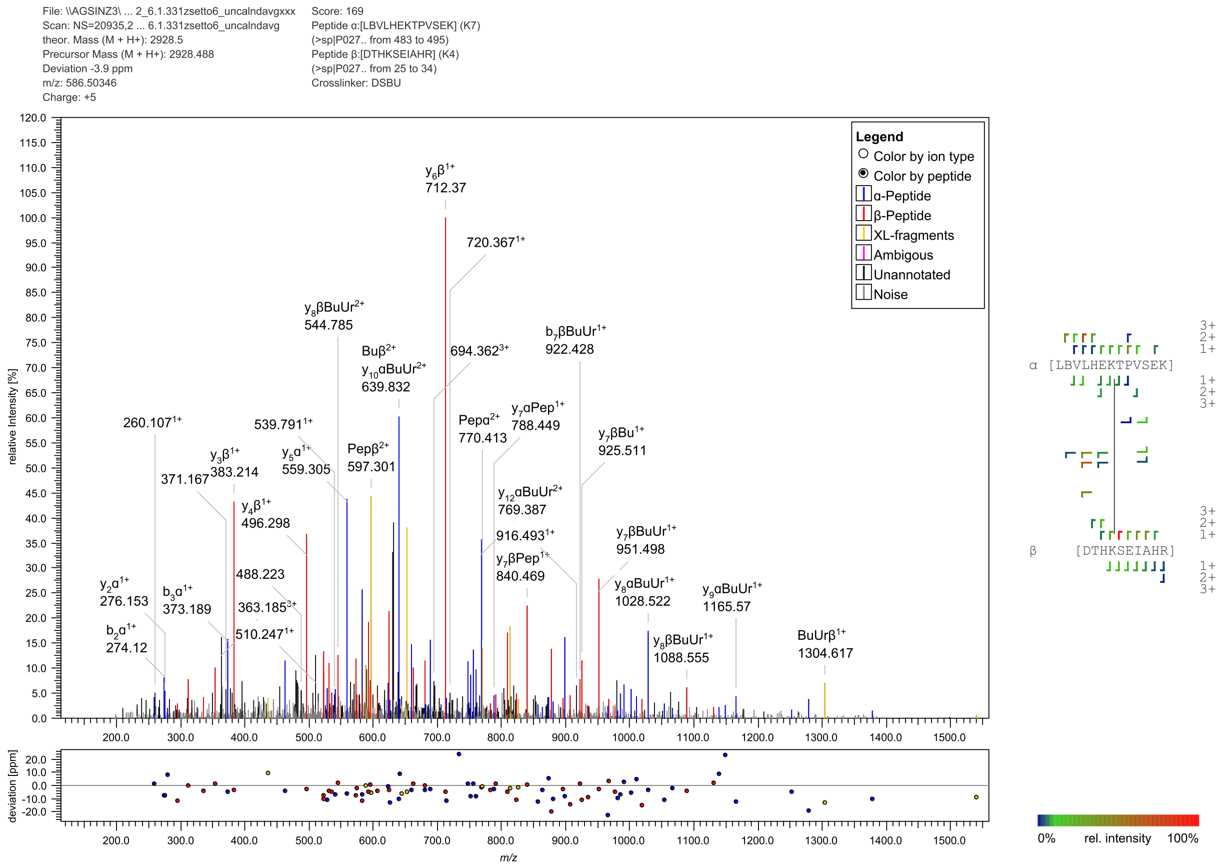

Figure S9. Fragment ion mass spectrum of DPFU cross-link analyzed by MeroX; b- and y-type ions are shown in blue and red; fragment ions of the cross-linker are shown in yellow. B in the amino acid sequence indicates carbamidomethylated Cys.

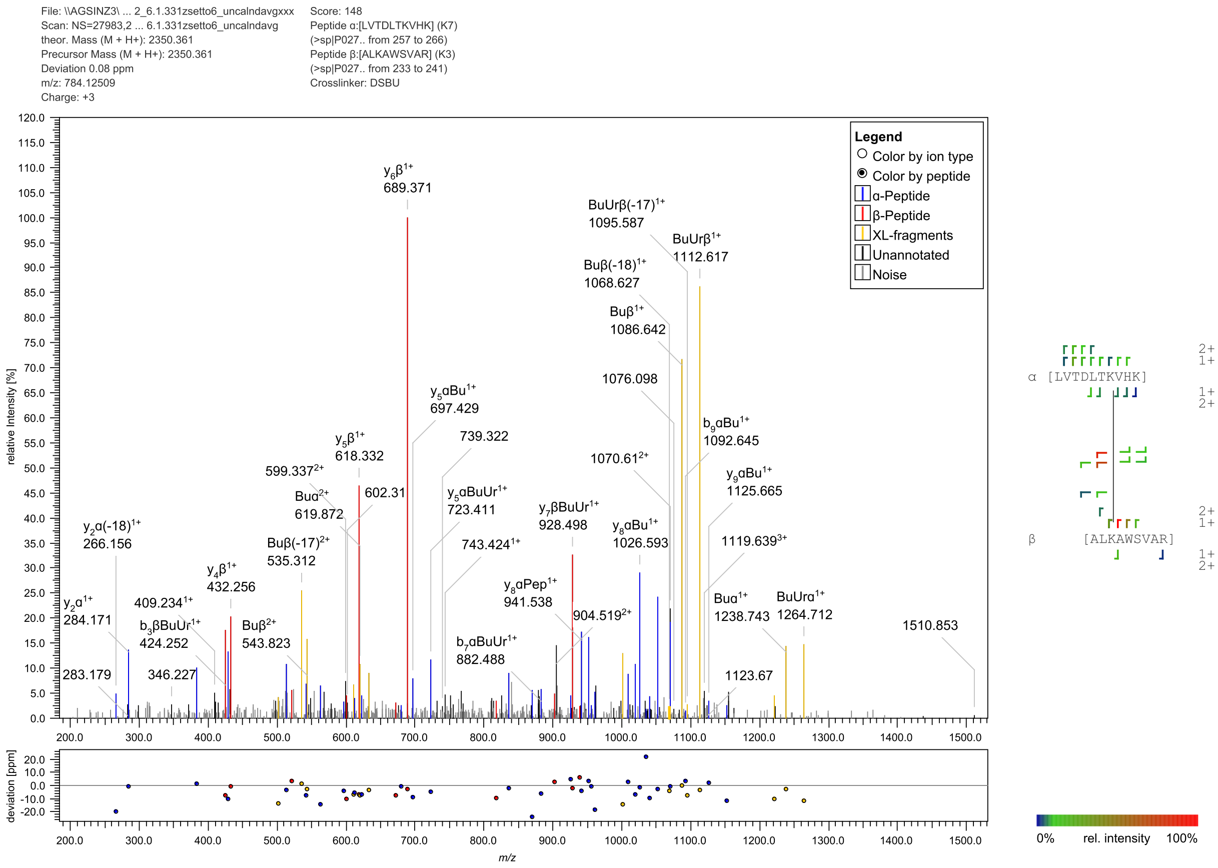

Figure S10. Fragment ion mass spectrum of DPFU cross-link analyzed by MeroX; b- and y-type ions are shown in blue and red; fragment ions of the cross-linker are shown in yellow.

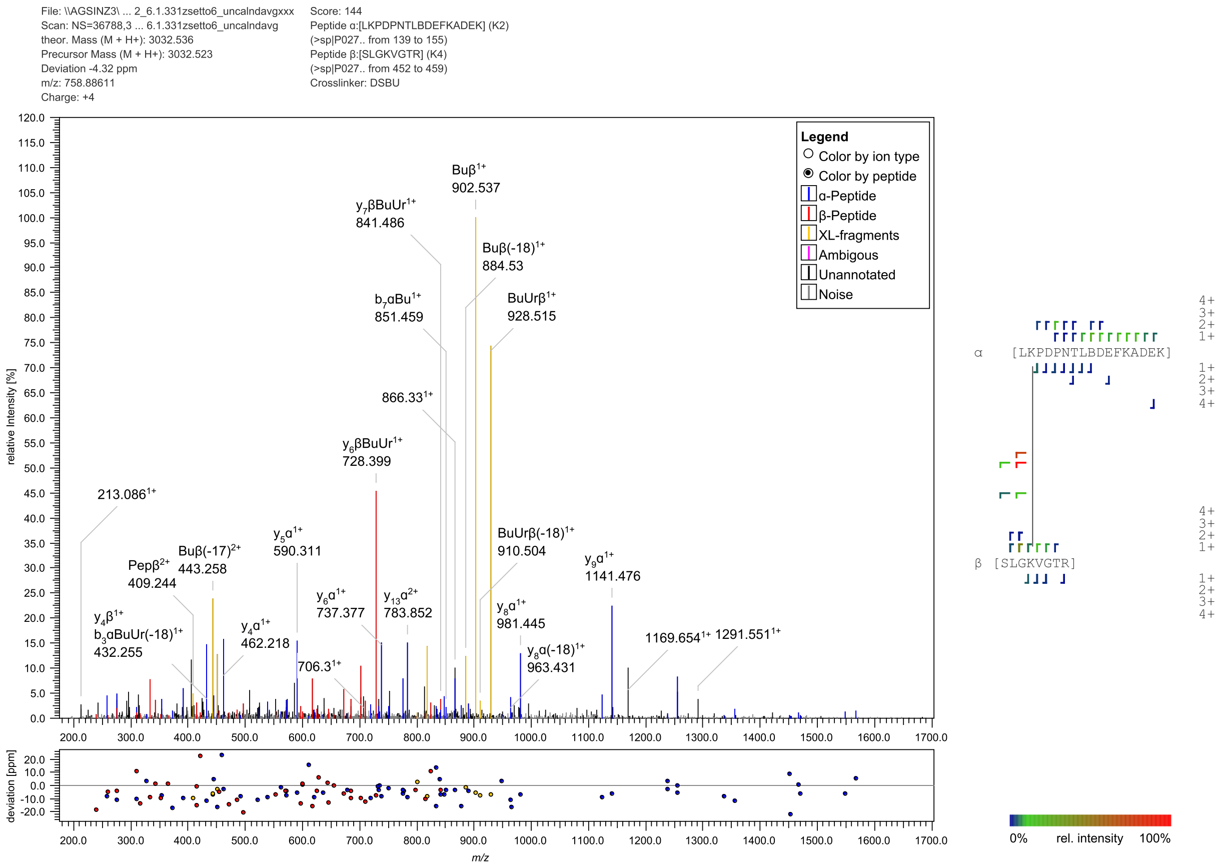

Figure S11. Fragment ion mass spectrum of DPFU cross-link analyzed by MeroX; b- and y-type ions are shown in blue and red; fragment ions of the cross-linker are shown in yellow. B in the amino acid sequence indicates carbamidomethylated Cys.

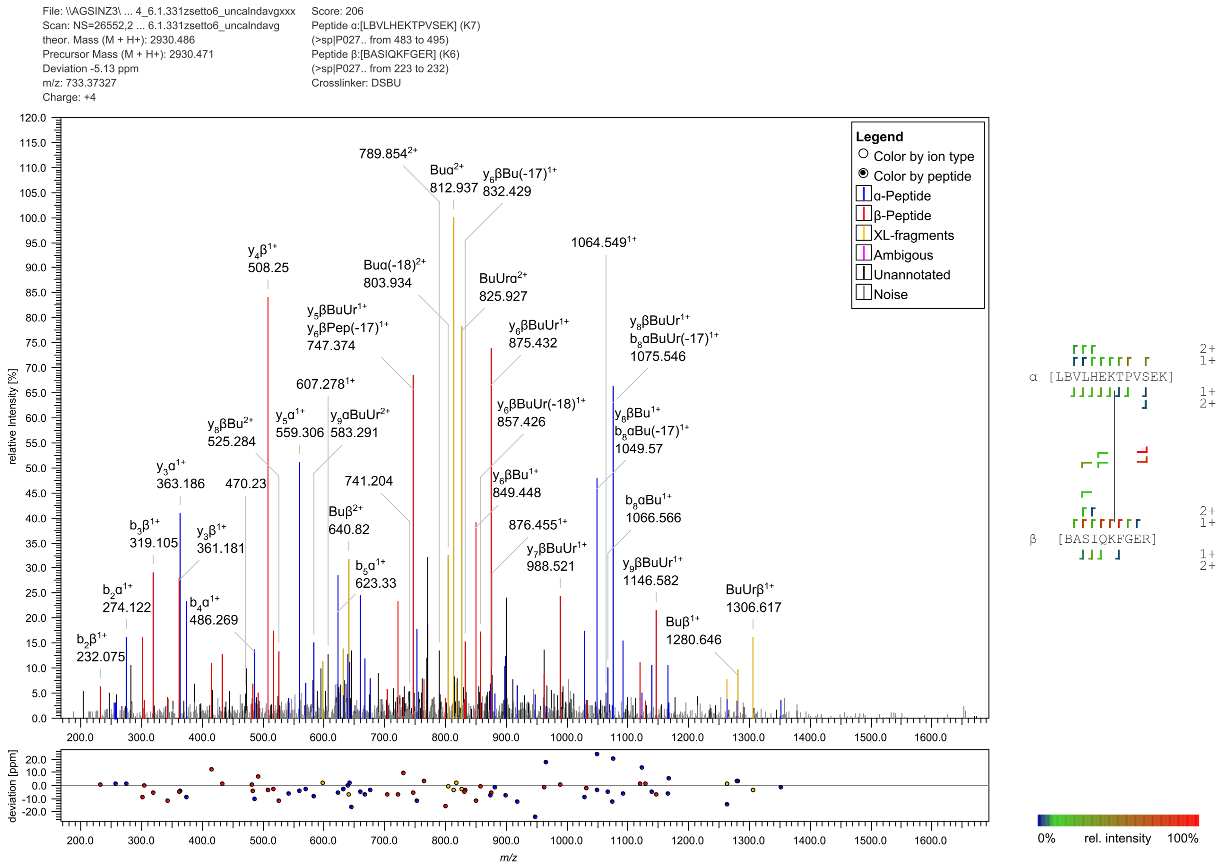

Figure S12. Fragment ion mass spectrum of DPFU cross-link analyzed by MeroX; b- and y-type ions are shown in blue and red; fragment ions of the cross-linker are shown in yellow. B in the amino acid sequence indicates carbamidomethylated Cys.

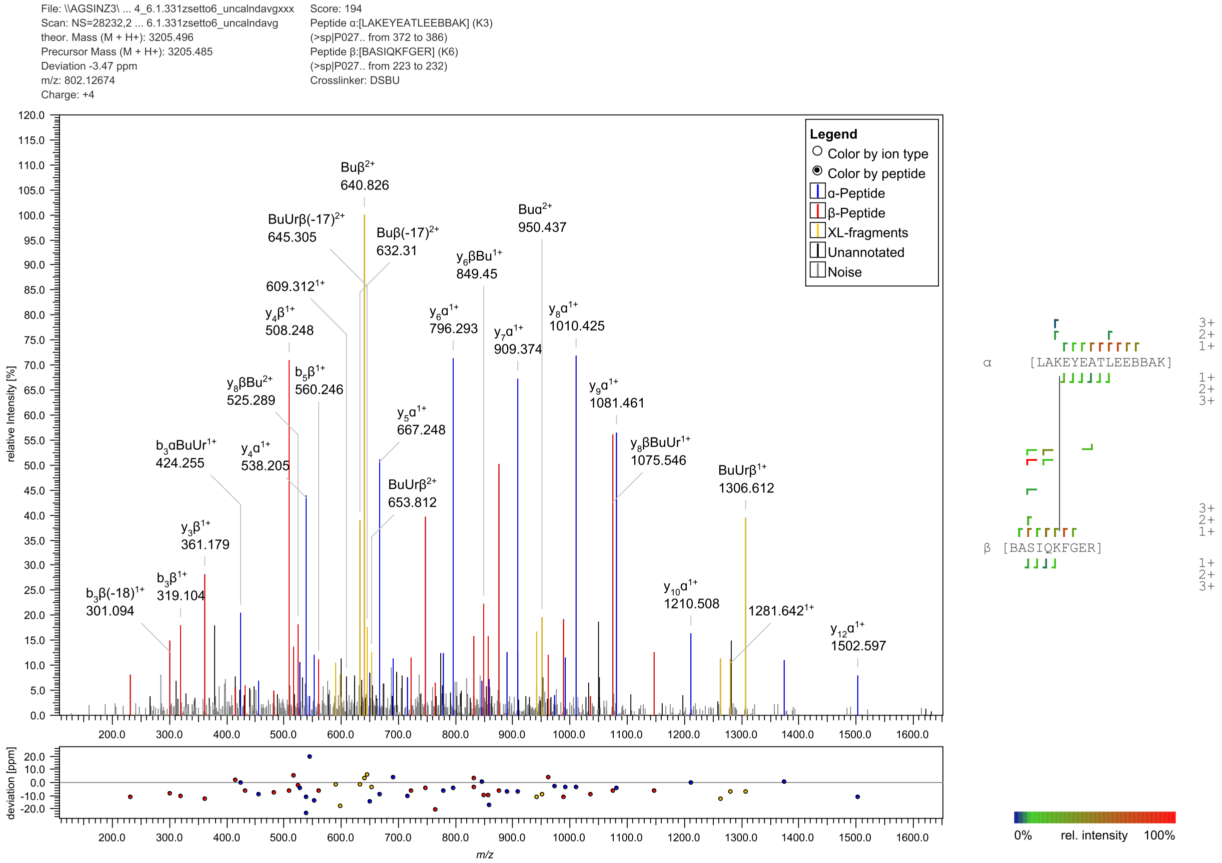

Figure S13. Fragment ion mass spectrum of DPFU cross-link analyzed by MeroX; b- and y-type ions are shown in blue and red; fragment ions of the cross-linker are shown in yellow. B in the amino acid sequence indicates carbamidomethylated Cys.

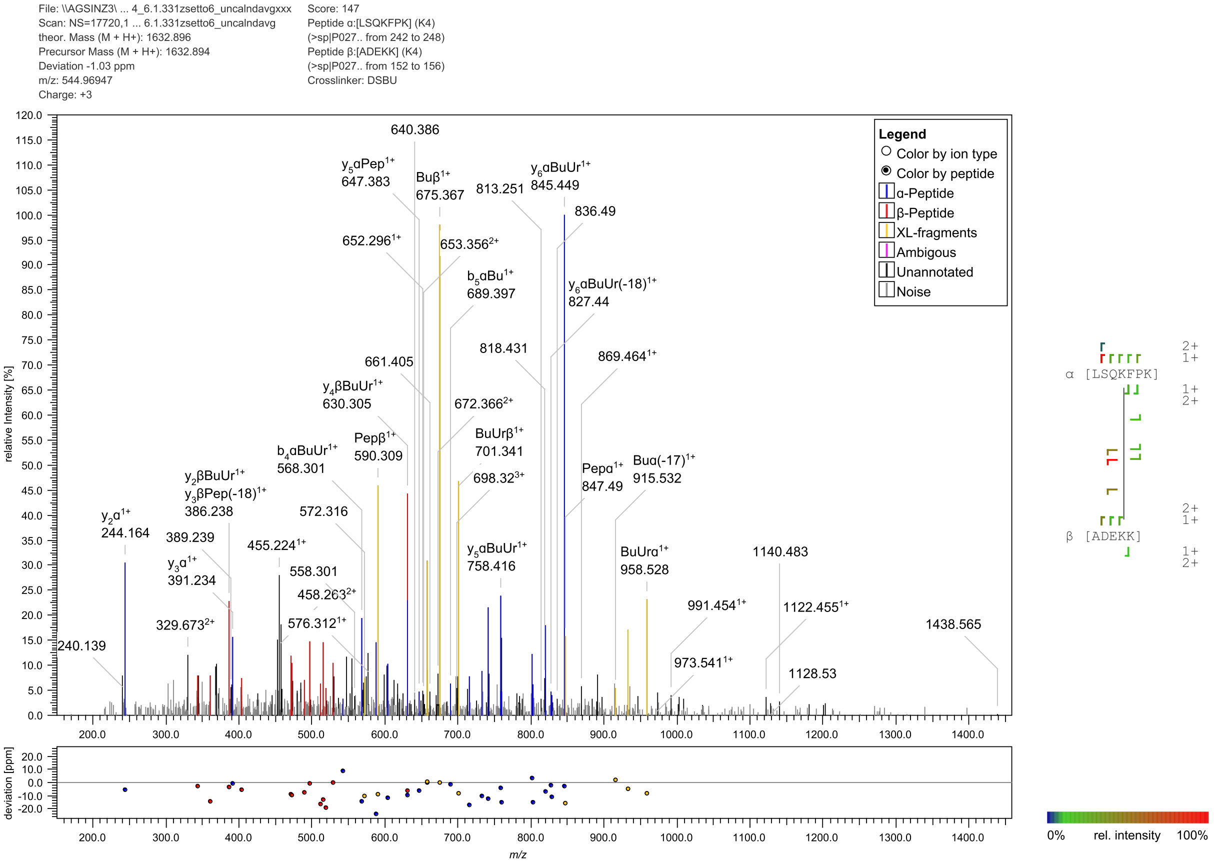

Figure S14. Fragment ion mass spectrum of DPFU cross-link analyzed by MeroX; b- and y-type ions are shown in blue and red; fragment ions of the cross-linker are shown in yellow.

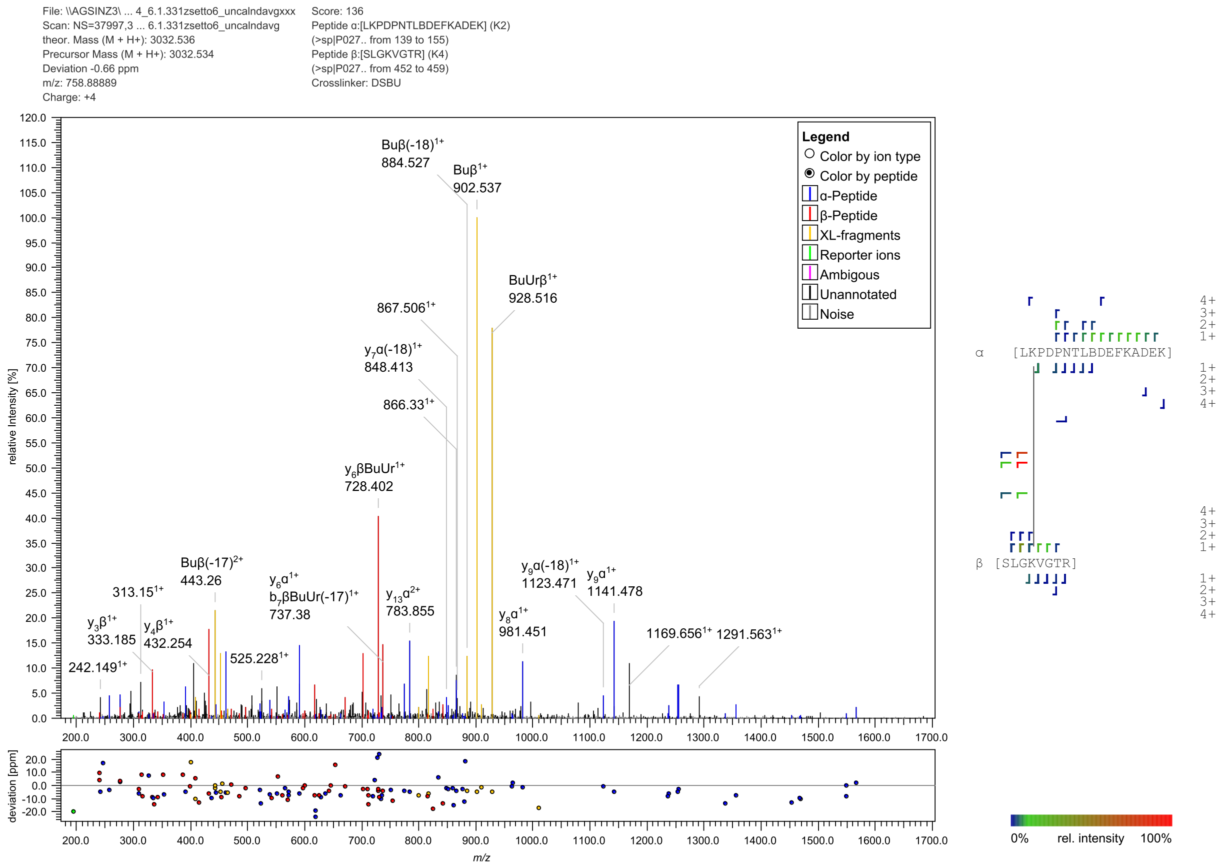

Figure S15. Fragment ion mass spectrum of DPFU cross-link analyzed by MeroX; b- and y-type ions are shown in blue and red; fragment ions of the cross-linker are shown in yellow. B in the amino acid sequence indicates carbamidomethylated Cys.

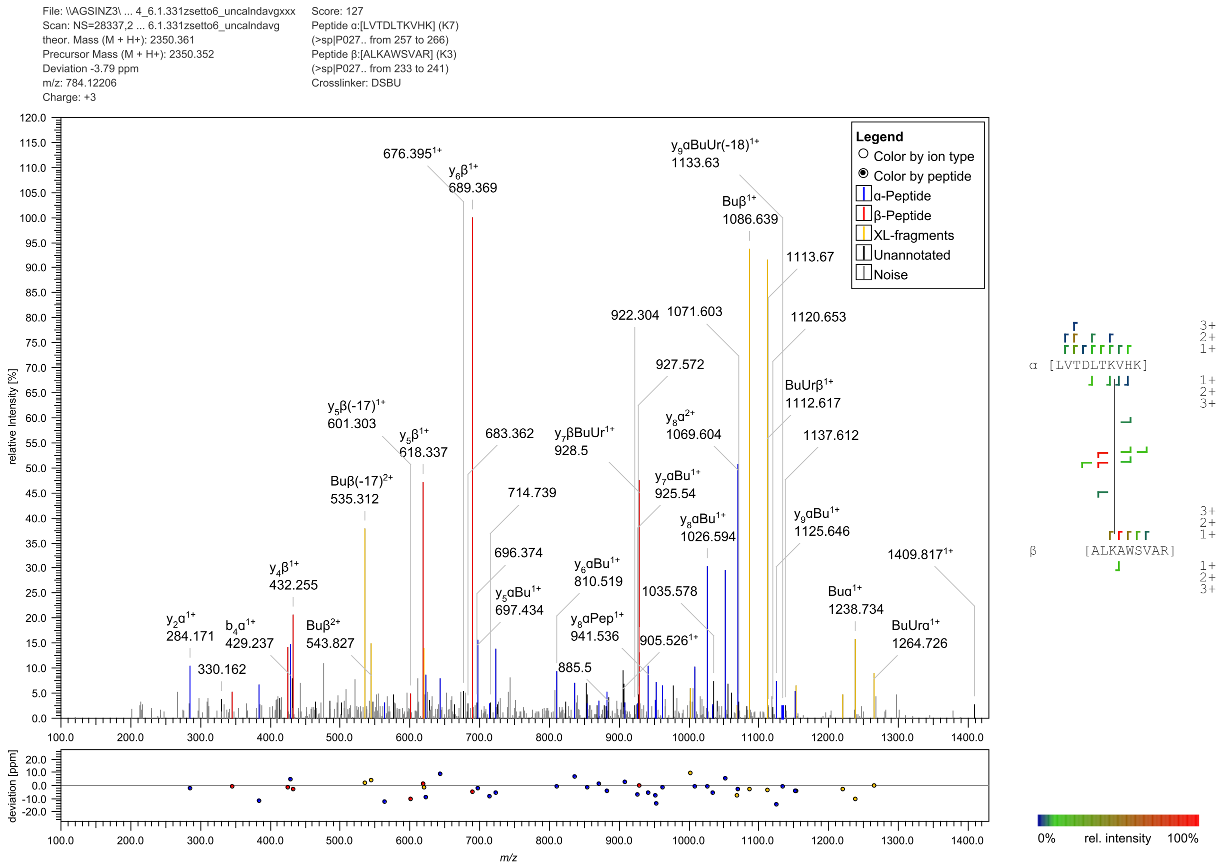

Figure S16. Fragment ion mass spectrum of DPFU cross-link analyzed by MeroX; b- and y-type ions are shown in blue and red; fragment ions of the cross-linker are shown in yellow.

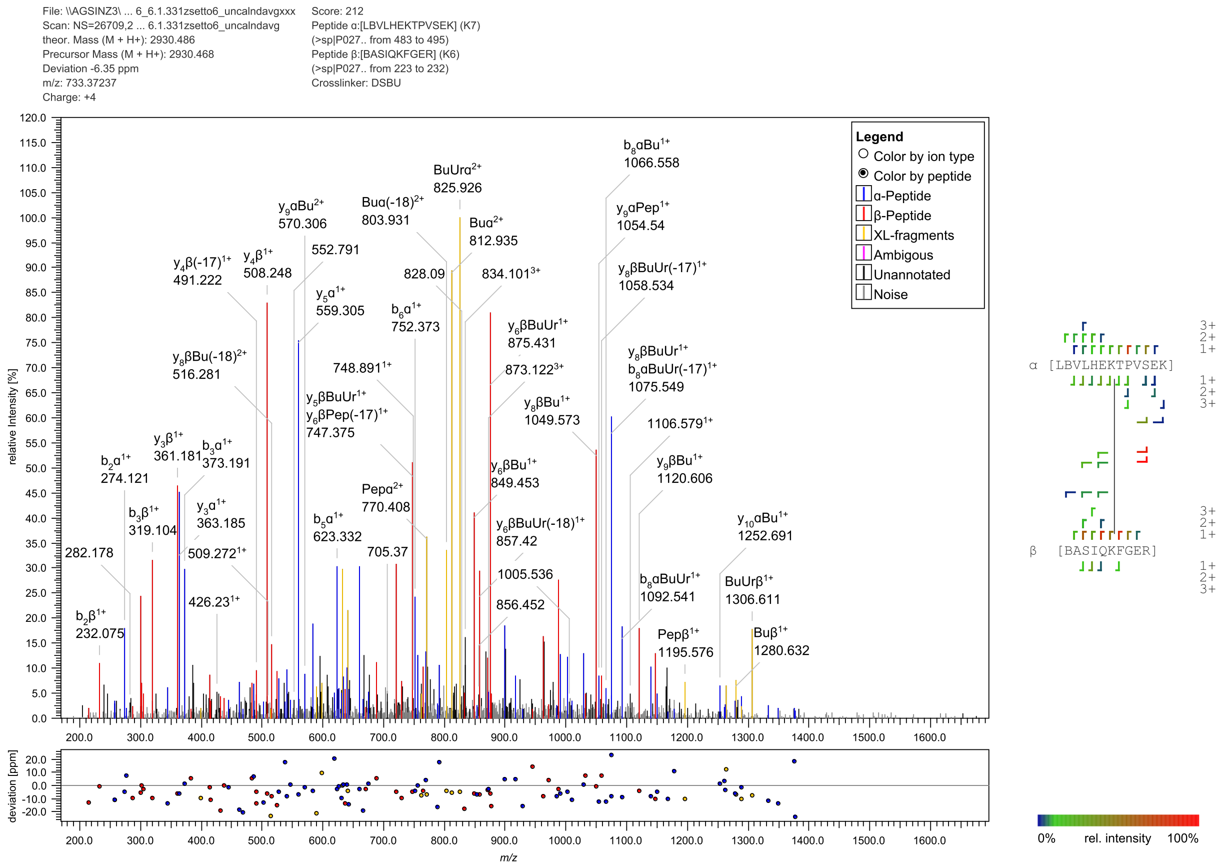

Figure S17. Fragment ion mass spectrum of DPFU cross-link analyzed by MeroX; b- and y-type ions are shown in blue and red; fragment ions of the cross-linker are shown in yellow. B in the amino acid sequence indicates carbamidomethylated Cys.

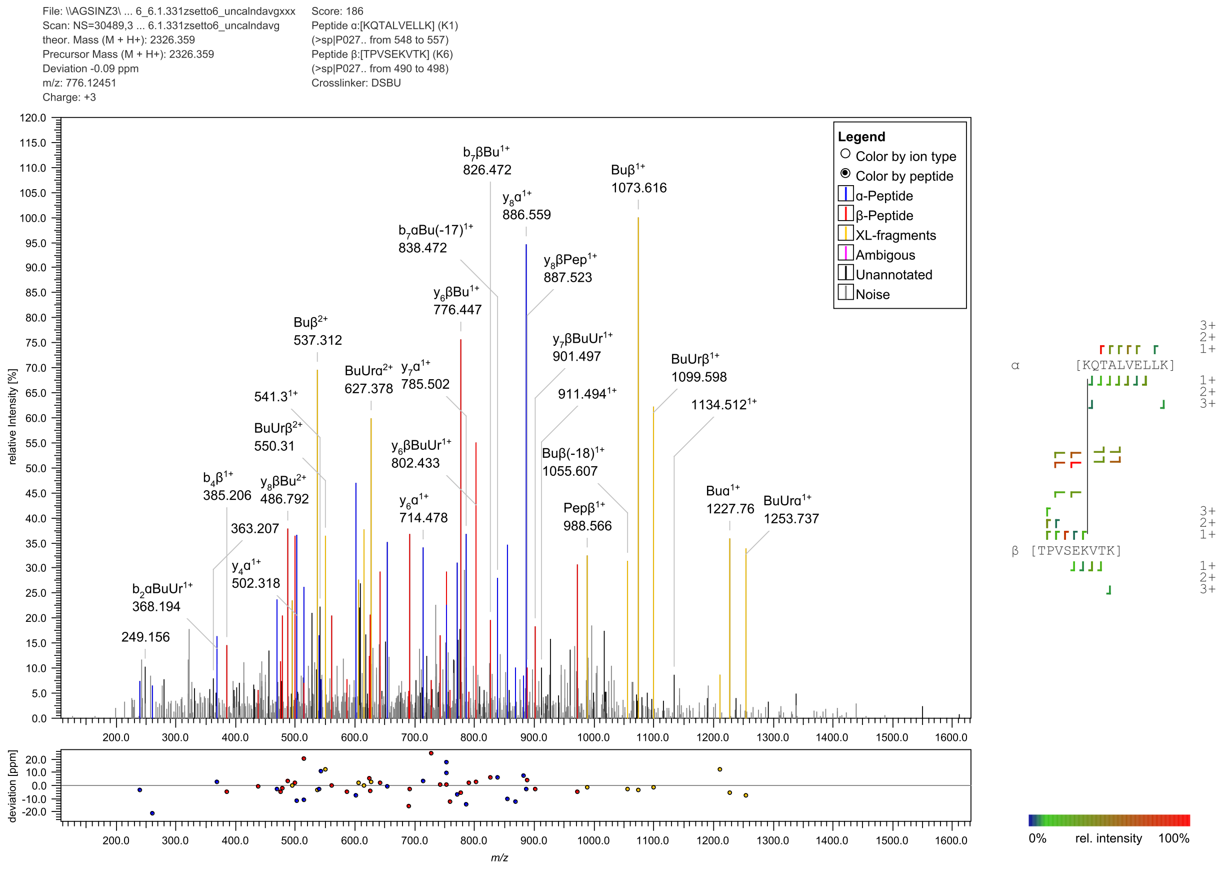

Figure S18. Fragment ion mass spectrum of DPFU cross-link analyzed by MeroX; b- and y-type ions are shown in blue and red; fragment ions of the cross-linker are shown in yellow.

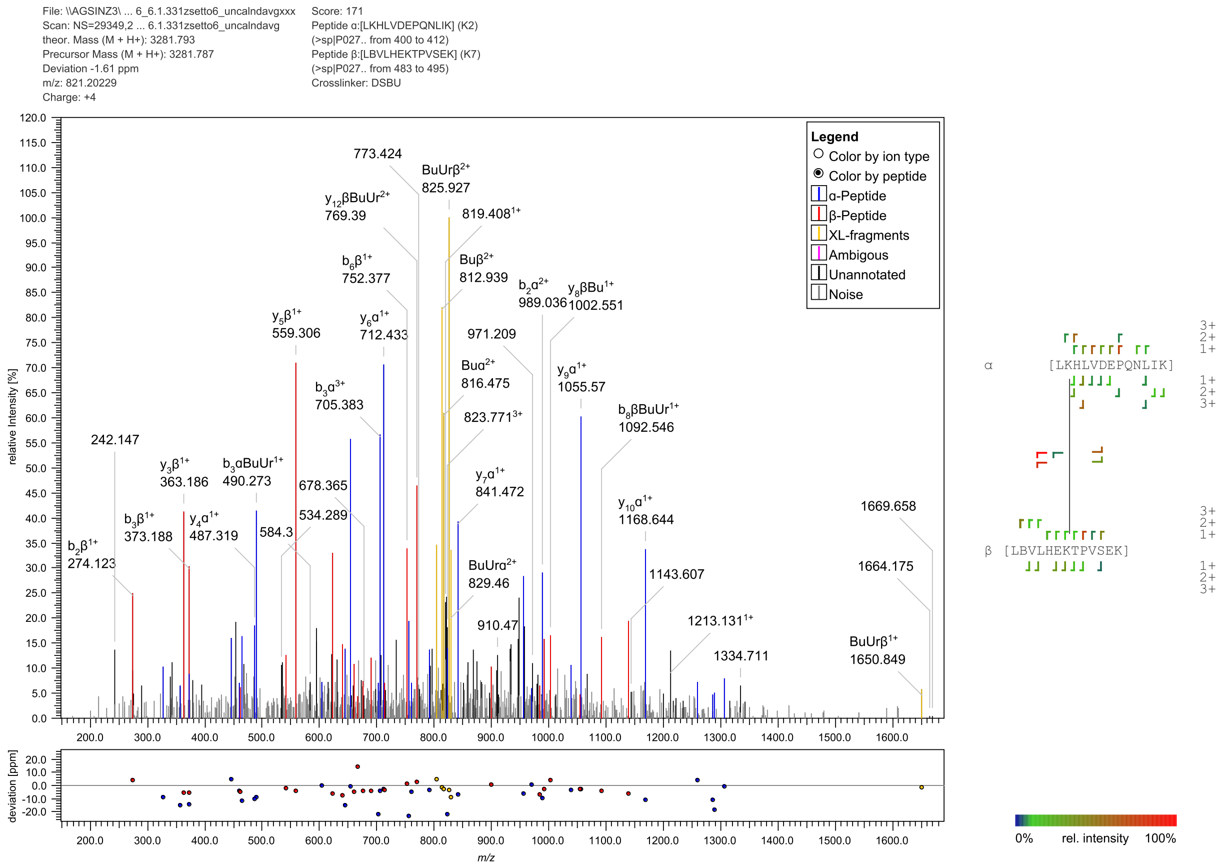

Figure S19. Fragment ion mass spectrum of DPFU cross-link analyzed by MeroX; b- and y-type ions are shown in blue and red; fragment ions of the cross-linker are shown in yellow. B in the amino acid sequence indicates carbamidomethylated Cys.

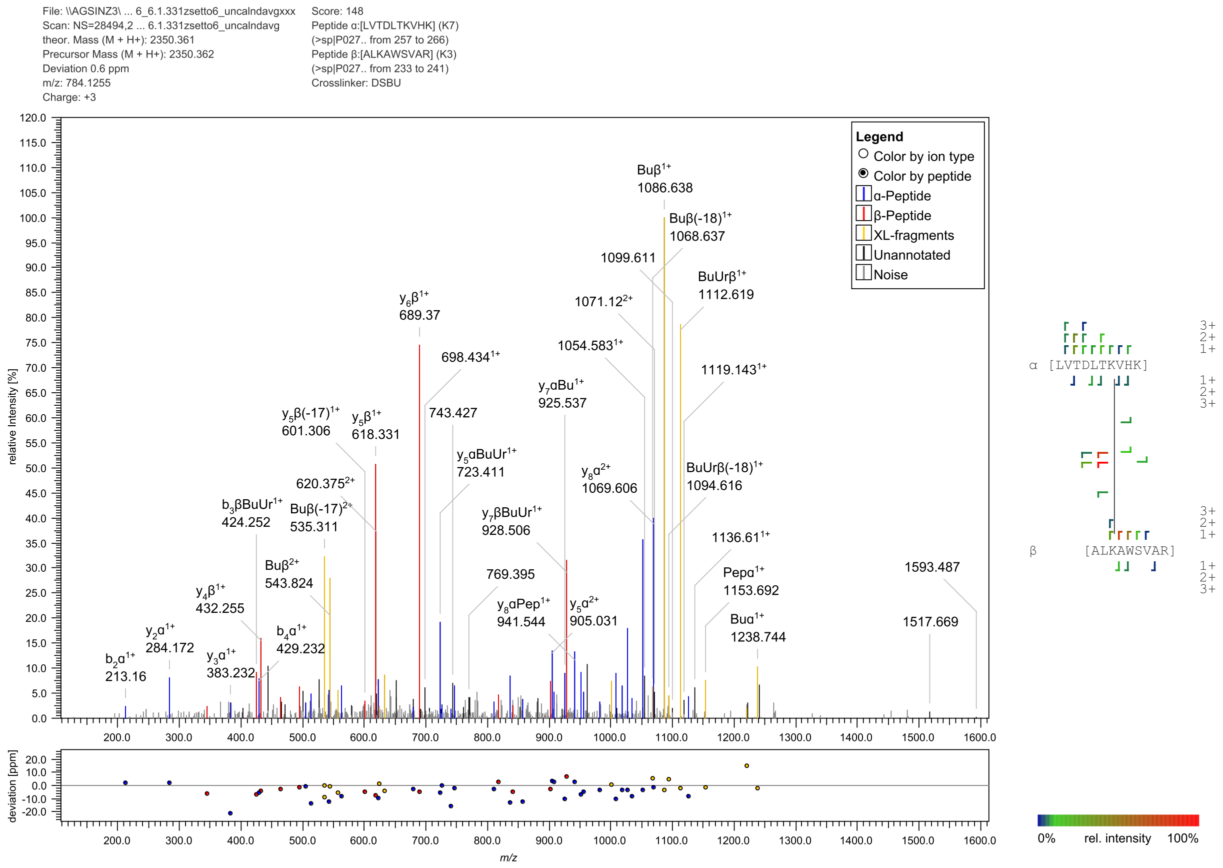

Figure S20. Fragment ion mass spectrum of DPFU cross-link analyzed by MeroX; b- and y-type ions are shown in blue and red; fragment ions of the cross-linker are shown in yellow.

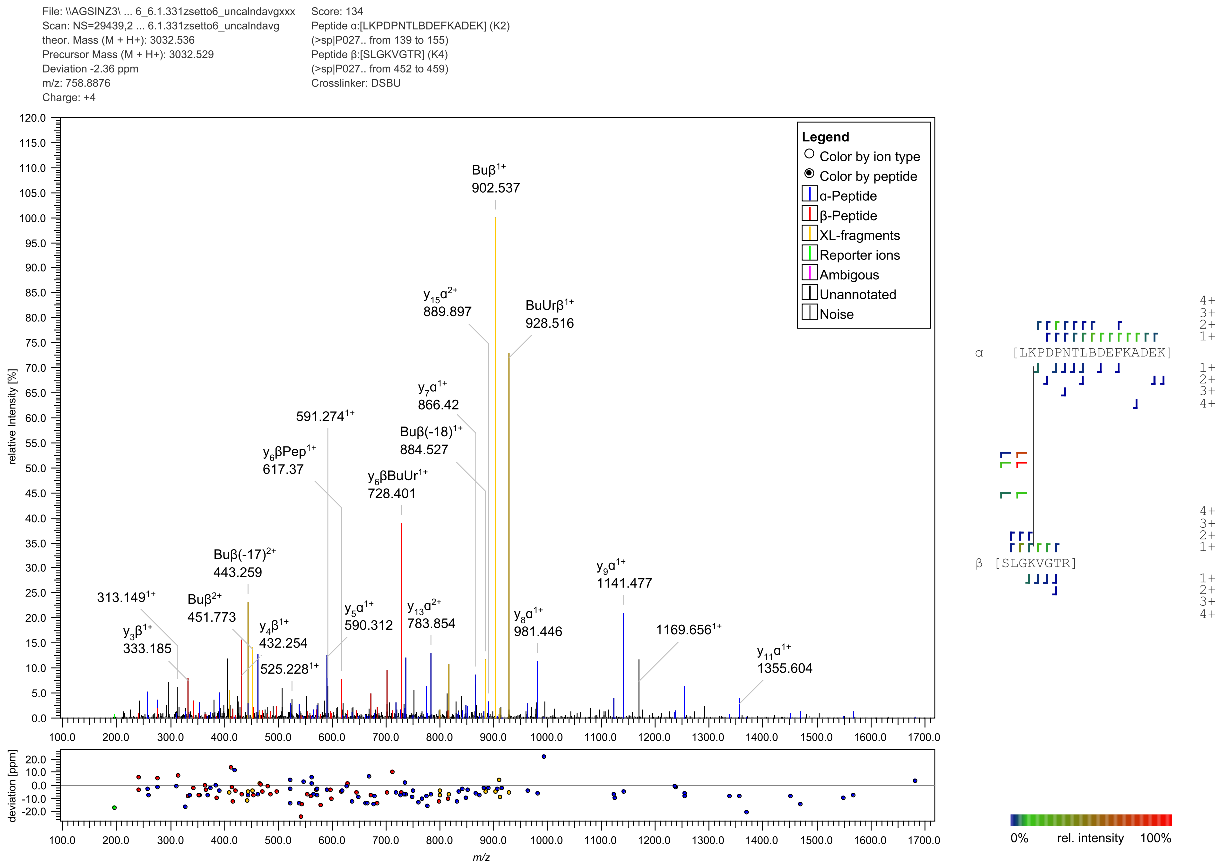

Figure S21. Fragment ion mass spectrum of DPFU cross-link analyzed by MeroX; b- and y-type ions are shown in blue and red; fragment ions of the cross-linker are shown in yellow. B in the amino acid sequence indicates carbamidomethylated Cys.

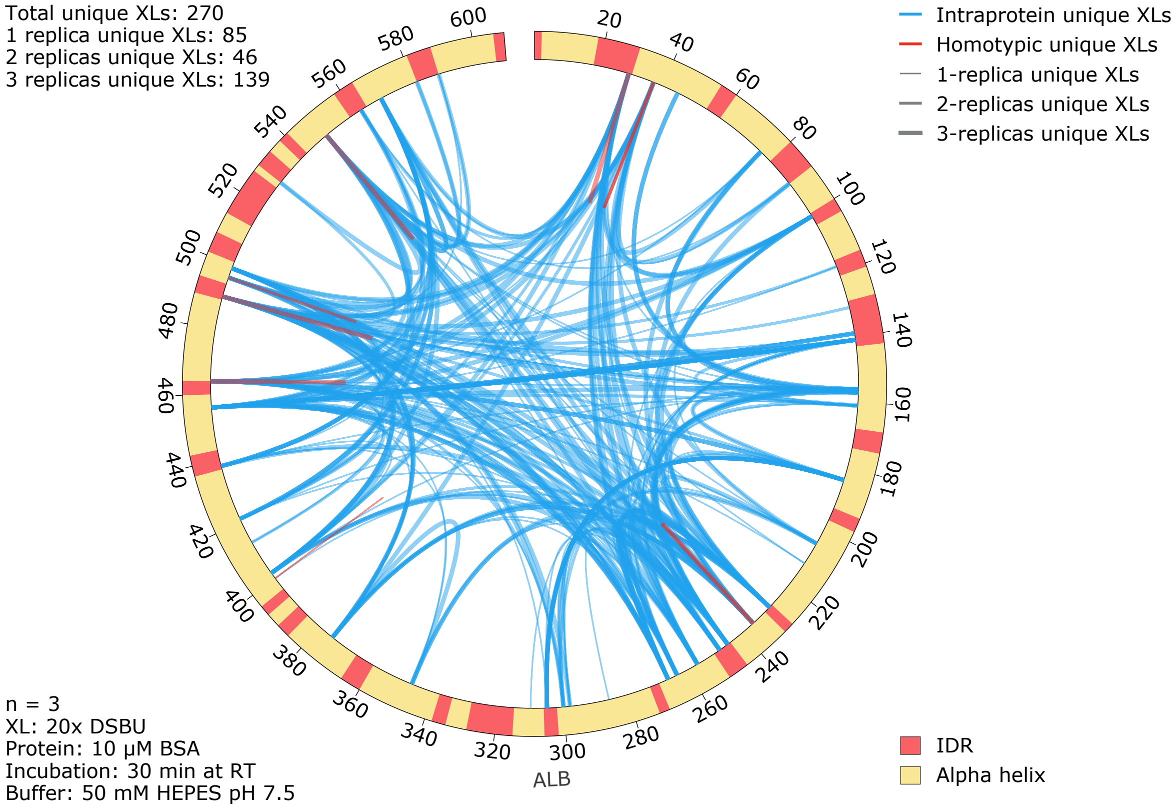

Figure S22. Circos plot of DSBU cross-links (20-fold molar excess) identified for BSA (30 min, room temperature, no SDS). Cross-links were identified using MeroX. Thickness of lines represents how often a specific cross-link was identified (1/3, 2/3 or 3/3 experiments). Intraprotein cross-links in BSA are shown in sky blue; interprotein (homotypic) cross-links occurred due to dimerization of BSA and are shown in red. In the schematic representation of BSA, α-helices are colored Naples yellow, intrinsically disordered regions (IDRs) are shown in coral red.

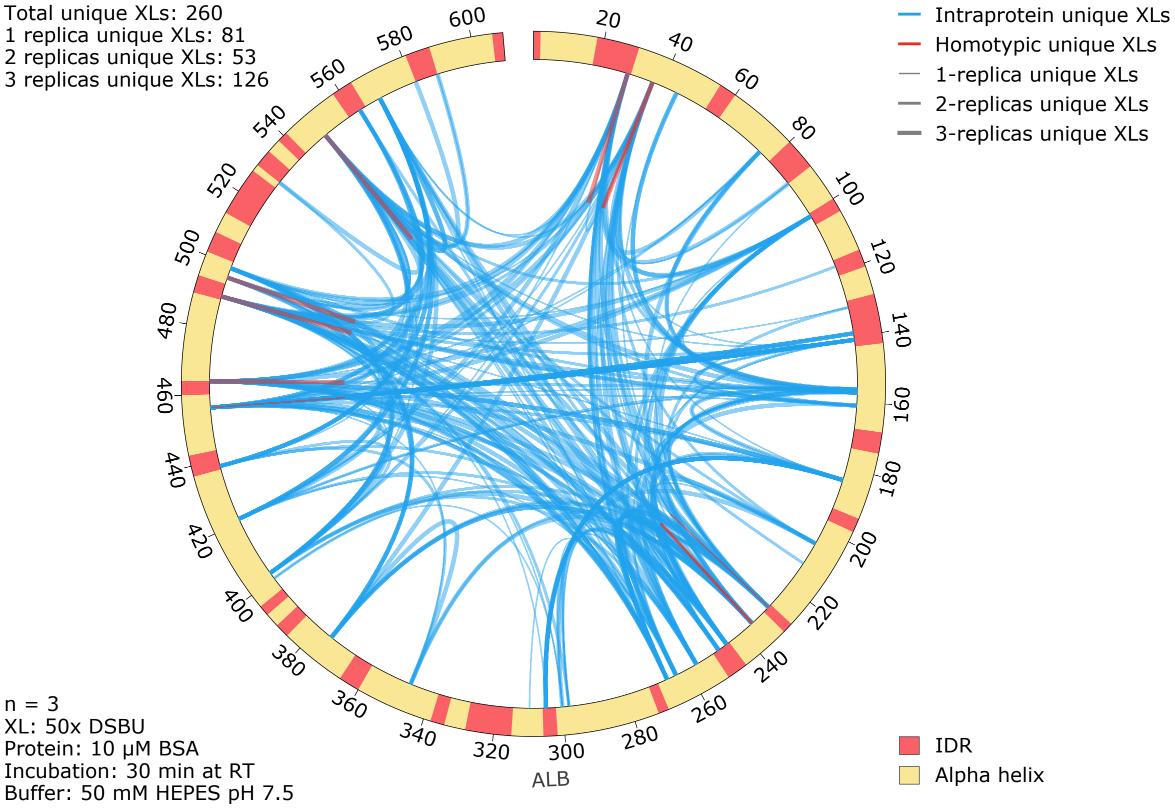

Figure S23. Circos plot of DSBU cross-links (50-fold molar excess) identified for BSA (30 min, room temperature, no SDS). Cross-links were identified using MeroX. Thickness of lines represents how often a specific cross-link was identified (1/3, 2/3 or 3/3 experiments). Intraprotein cross-links in BSA are shown in sky blue; interprotein (homotypic) cross-links occurred due to dimerization of BSA and are shown in red. In the schematic representation of BSA, α-helices are colored Naples yellow, intrinsically disordered regions (IDRs) are shown in coral red.

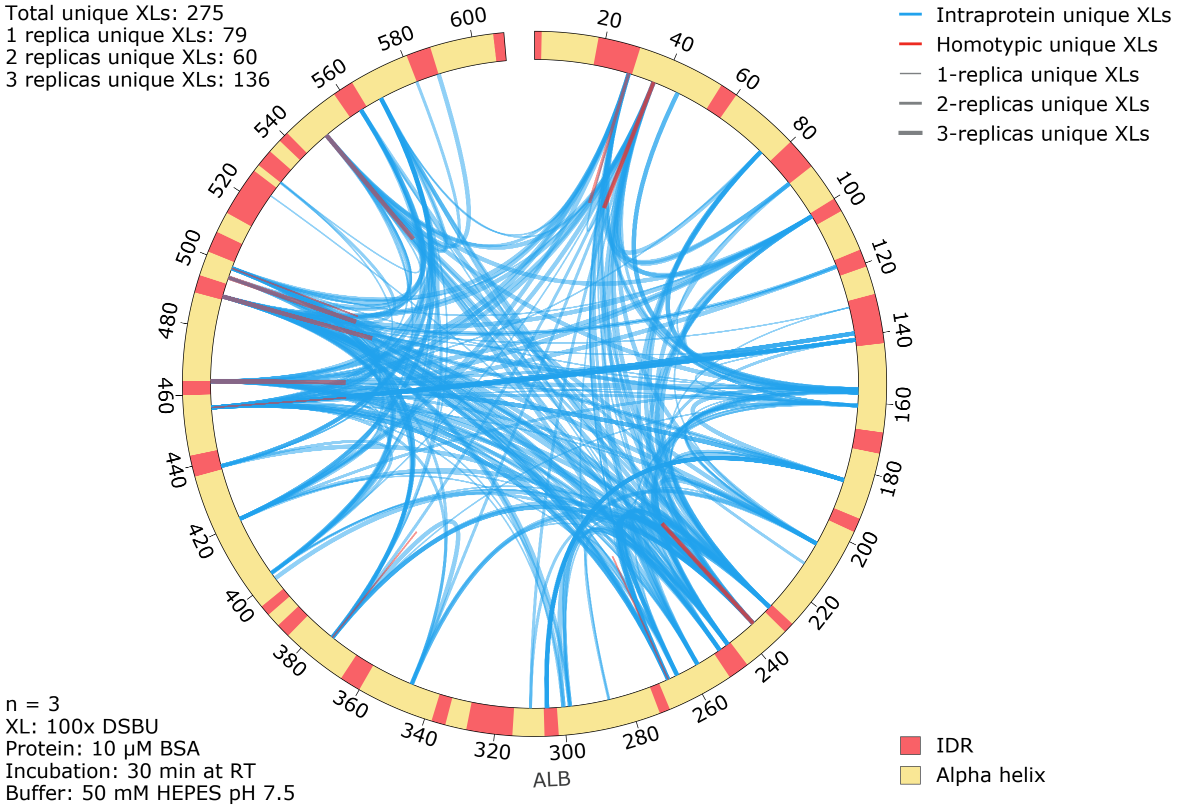

Figure S24. Circos plot of DSBU cross-links (100-fold molar excess) identified for BSA (30 min, room temperature, no SDS). Cross-links were identified using MeroX. Thickness of lines represents how often a specific cross-link was identified (1/3, 2/3 or 3/3 experiments). Intraprotein cross-links in BSA are shown in sky blue; interprotein (homotypic) cross-links occurred due to dimerization of BSA and are shown in red. In the schematic representation of BSA, α-helices are colored Naples yellow, intrinsically disordered regions (IDRs) are shown in coral red.

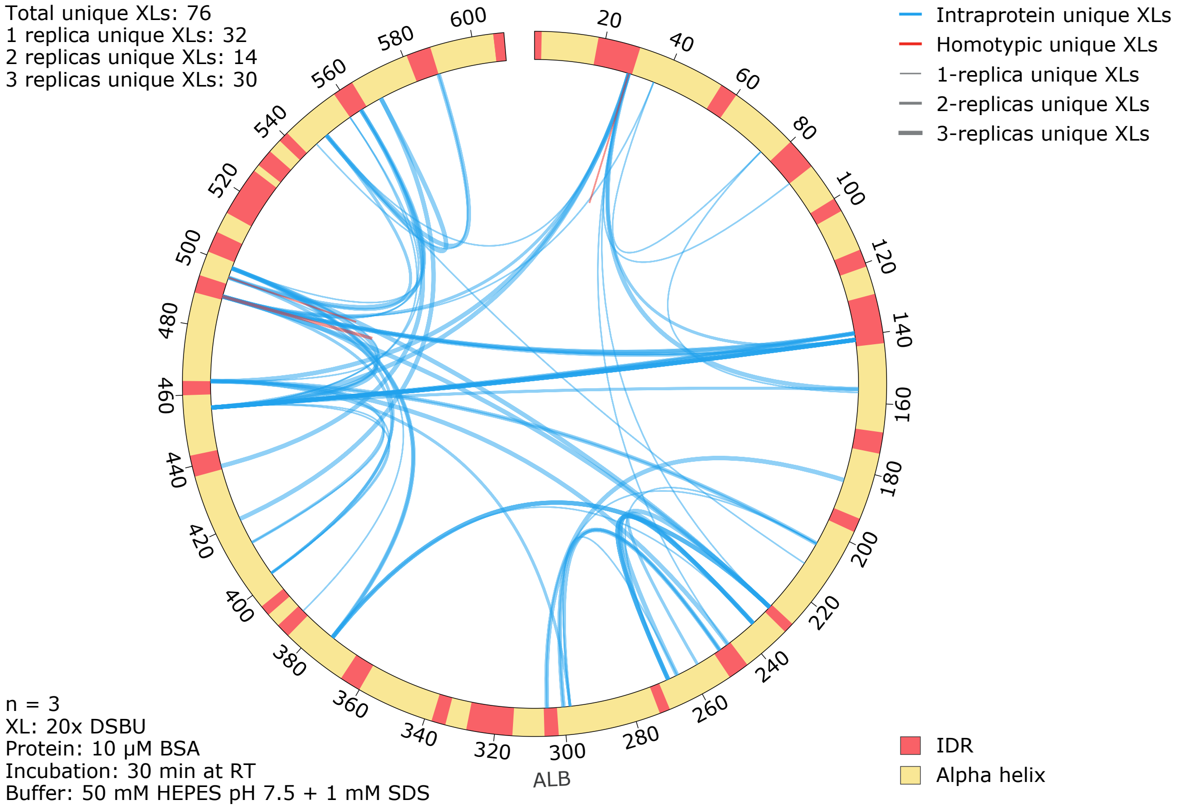

Figure S25. Circos plot of DSBU cross-links (20-fold molar excess) identified for BSA (30 min, room temperature, 1 mM SDS). Cross-links were identified using MeroX. Thickness of lines represents how often a specific cross-link was identified (1/3, 2/3 or 3/3 experiments). Intraprotein cross-links in BSA are shown in sky blue; interprotein (homotypic) cross-links occurred due to dimerization of BSA and are shown in red. In the schematic representation of BSA, α-helices are colored Naples yellow, intrinsically disordered regions (IDRs) are shown in coral red.

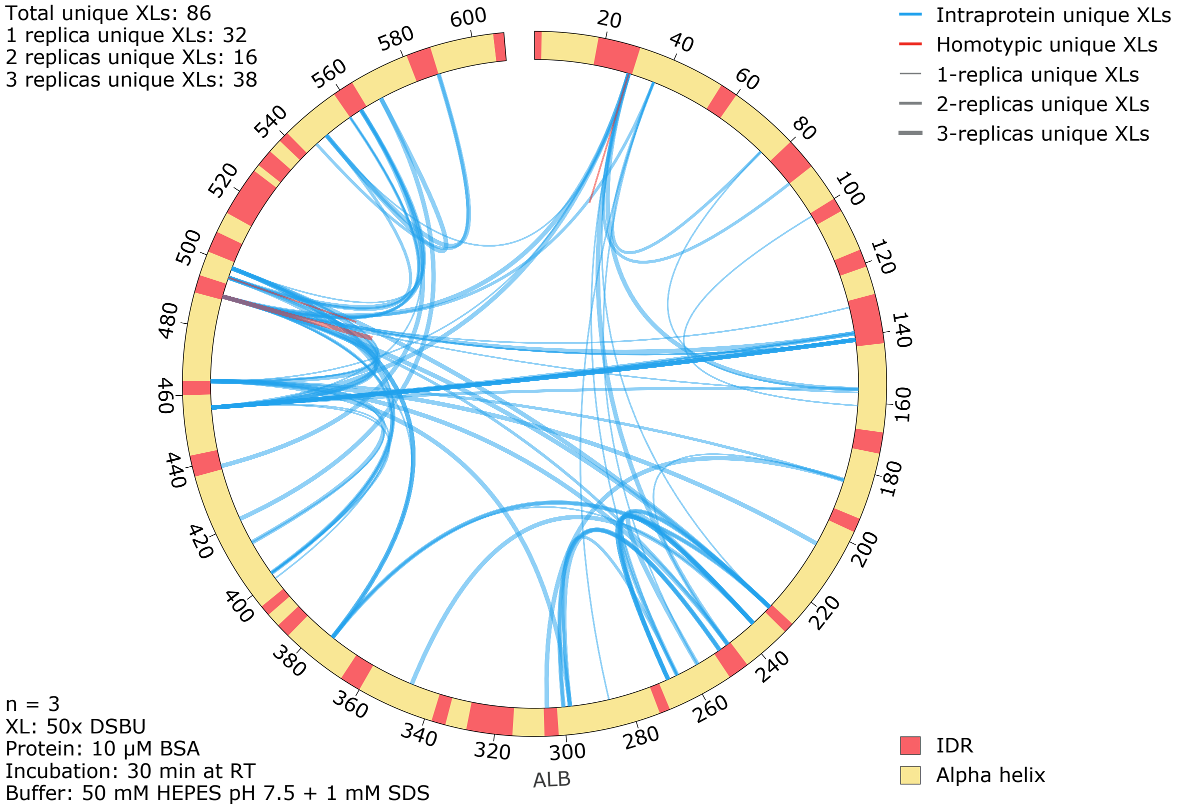

Figure S26. Circos plot of DSBU cross-links (50-fold molar excess) identified for BSA (30 min, room temperature, 1 mM SDS). Cross-links were identified using MeroX. Thickness of lines represents how often a specific cross-link was identified (1/3, 2/3 or 3/3 experiments). Intraprotein cross-links in BSA are shown in sky blue; interprotein (homotypic) cross-links occurred due to dimerization of BSA and are shown in red. In the schematic representation of BSA, α-helices are colored Naples yellow, intrinsically disordered regions (IDRs) are shown in coral red.

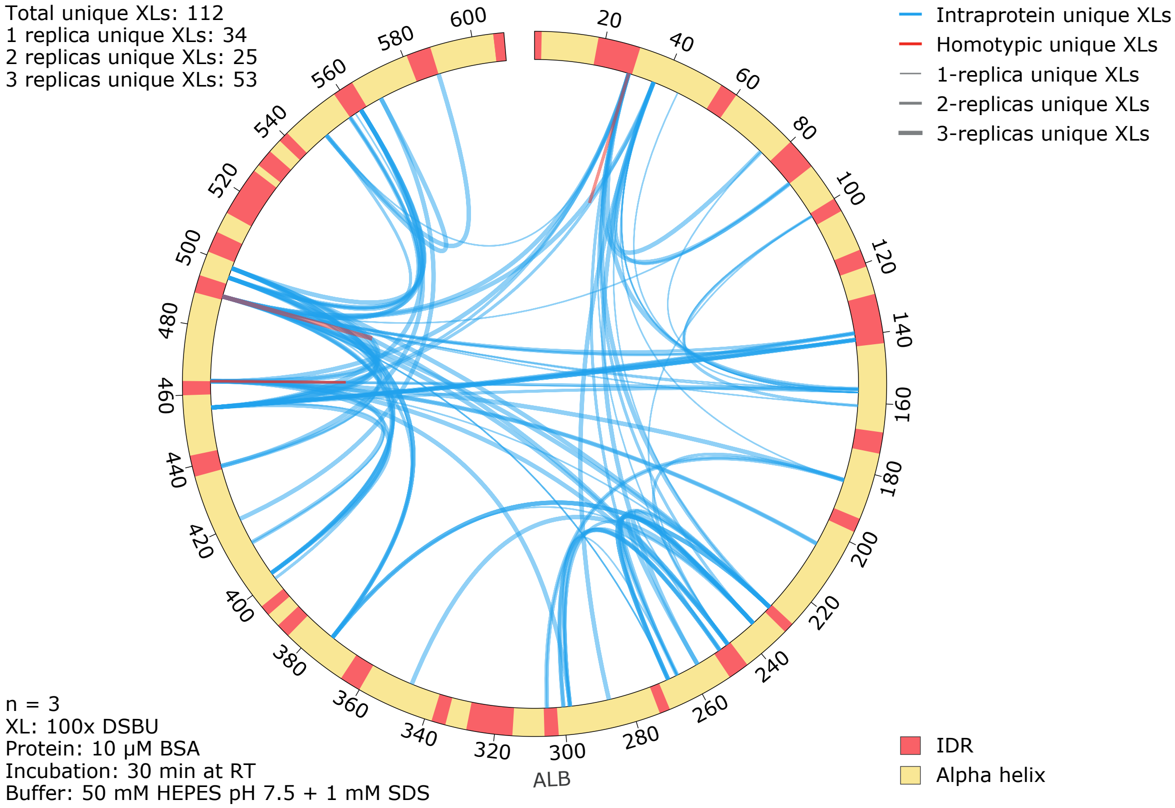

Figure S27. Circos plot of DSBU cross-links (100-fold molar excess) identified for BSA (30 min, room temperature, 1 mM SDS). Cross-links were identified using MeroX. Thickness of lines represents how often a specific cross-link was identified (1/3, 2/3 or 3/3 experiments). Intraprotein cross-links in BSA are shown in sky blue; interprotein (homotypic) cross-links occurred due to dimerization of BSA and are shown in red. In the schematic representation of BSA, α-helices are colored Naples yellow, intrinsically disordered regions (IDRs) are shown in coral red.

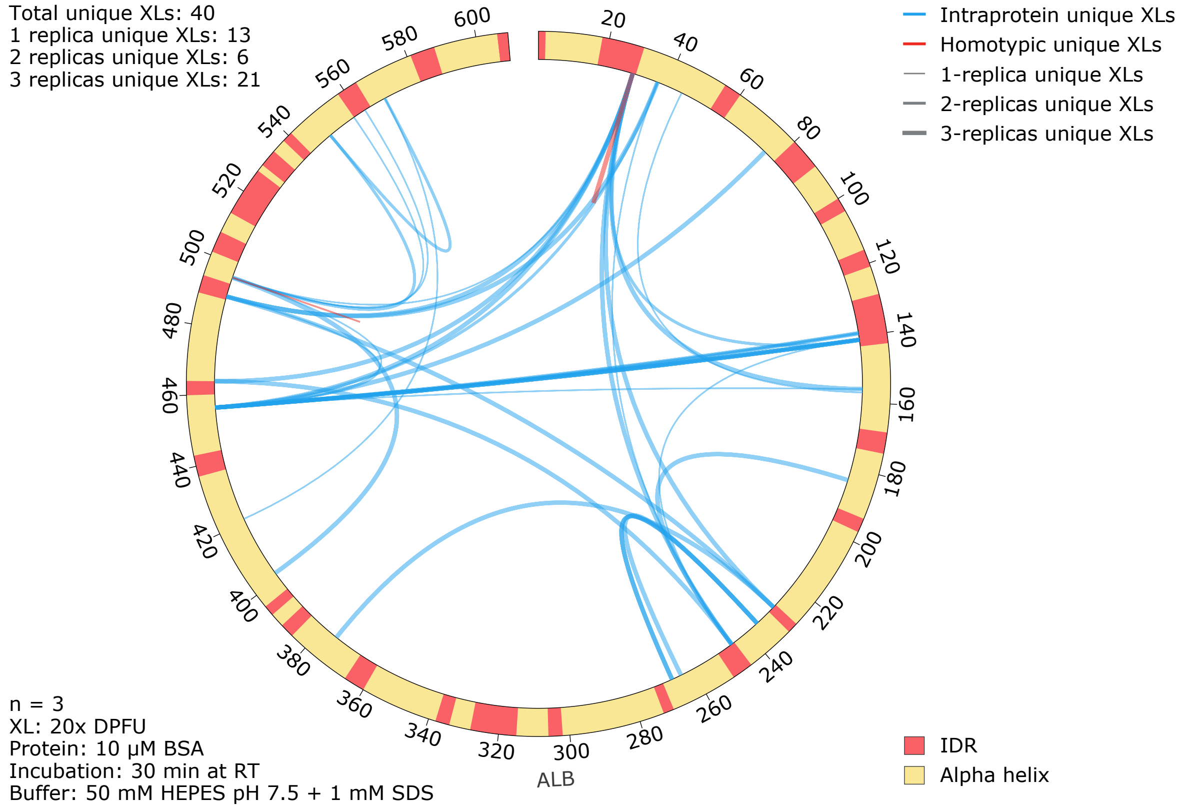

Figure S28. Circos plot of DPFU cross-links (20-fold molar excess) identified for BSA (30 min, room temperature, 1 mM SDS). Cross-links were identified using MeroX. Thickness of lines represents how often a specific cross-link was identified (1/3, 2/3 or 3/3 experiments). Intraprotein cross-links in BSA are shown in sky blue; interprotein (homotypic) cross-links occurred due to dimerization of BSA and are shown in red. In the schematic representation of BSA, α-helices are colored Naples yellow, intrinsically disordered regions (IDRs) are shown in coral red.

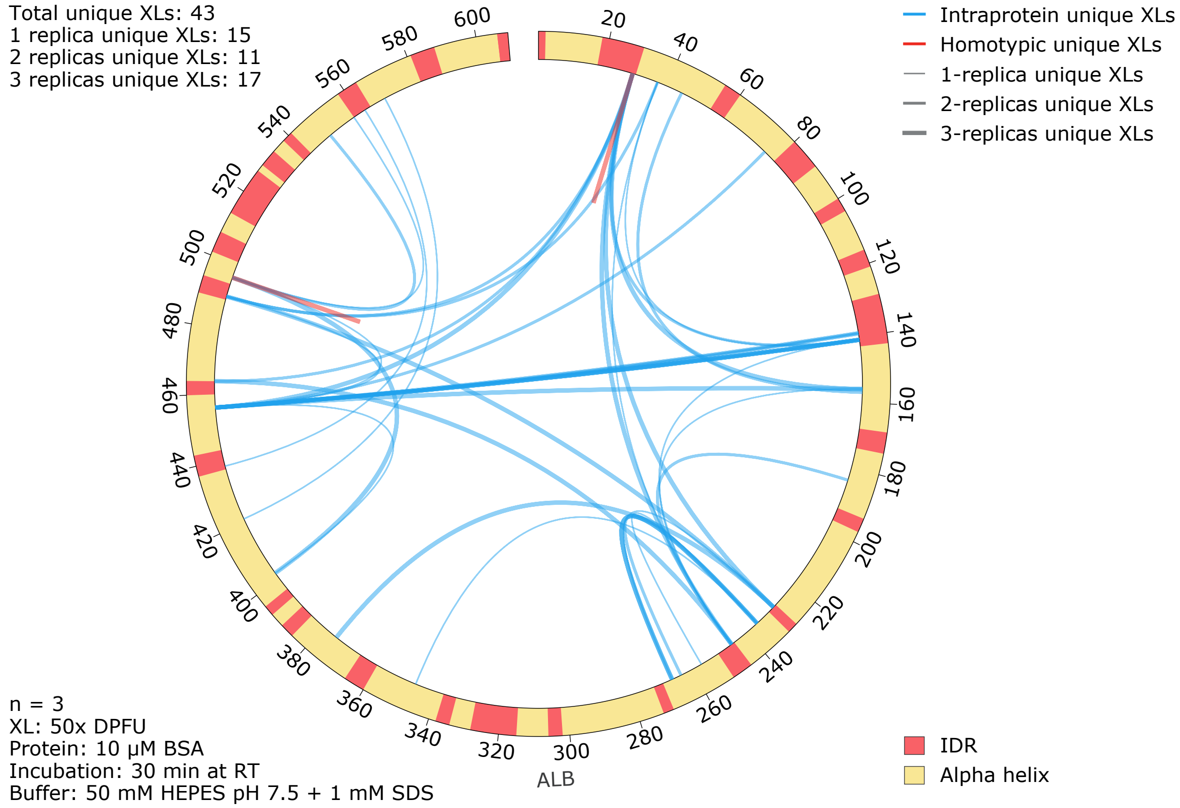

Figure S29. Circos plot of DPFU cross-links (50-fold molar excess) identified for BSA (30 min, room temperature, 1 mM SDS). Cross-links were identified using MeroX. Thickness of lines represents how often a specific cross-link was identified (1/3, 2/3 or 3/3 experiments). Intraprotein cross-links in BSA are shown in sky blue; interprotein (homotypic) cross-links occurred due to dimerization of BSA and are shown in red. In the schematic representation of BSA, α-helices are colored Naples yellow, intrinsically disordered regions (IDRs) are shown in coral red.

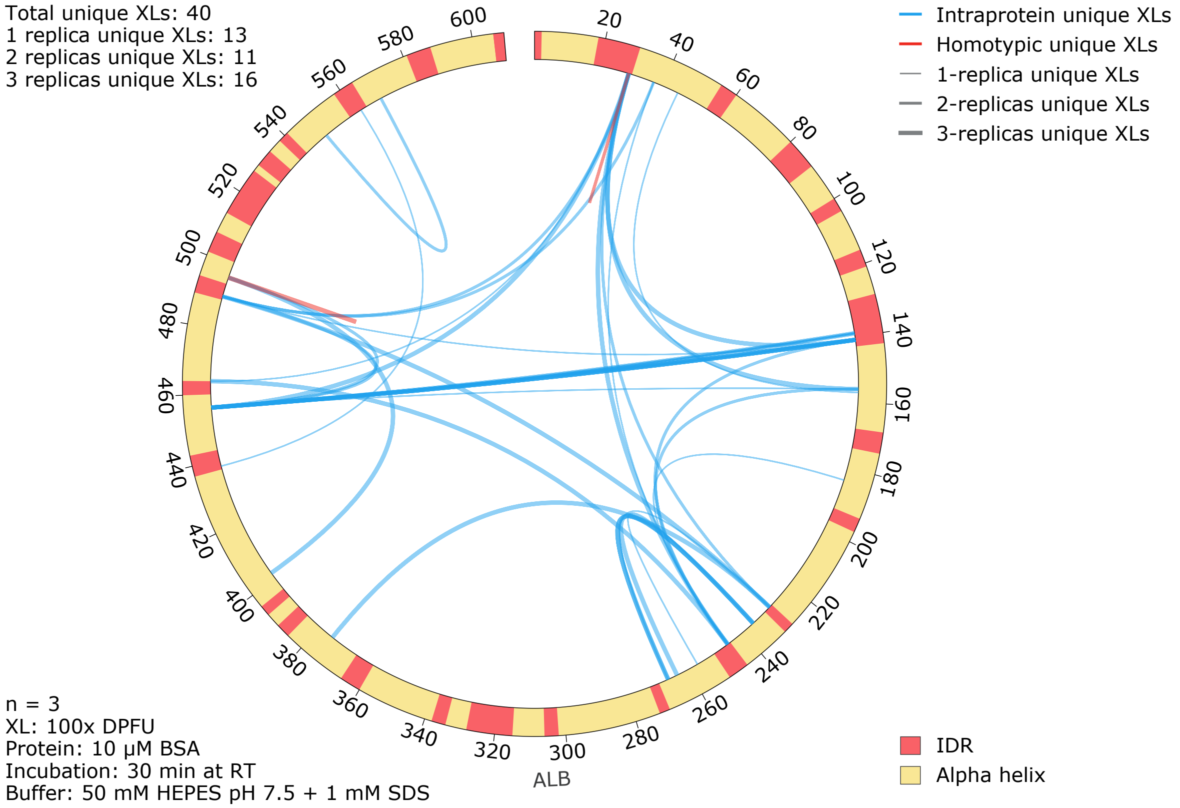

Figure S30. Circos plot of DPFU cross-links (100-fold molar excess) identified for BSA (30 min, room temperature, 1 mM SDS). Cross-links were identified using MeroX. Thickness of lines represents how often a specific cross-link was identified (1/3, 2/3 or 3/3 experiments). Intraprotein cross-links in BSA are shown in sky blue; interprotein (homotypic) cross-links occurred due to dimerization of BSA and are shown in red. In the schematic representation of BSA, α-helices are colored Naples yellow, intrinsically disordered regions (IDRs) are shown in coral red.

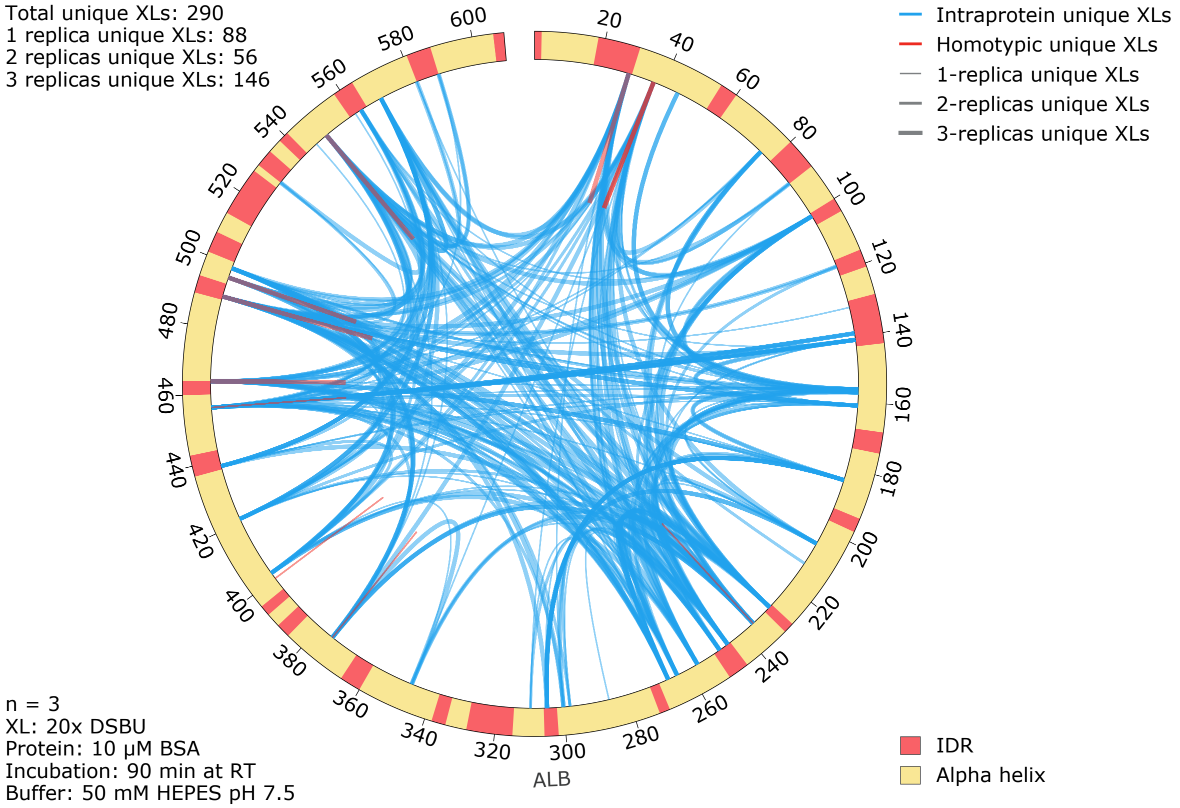

Figure S31. Circos plot of DSBU cross-links (20-fold molar excess) identified for BSA (90 min, room temperature, no SDS). Cross-links were identified using MeroX. Thickness of lines represents how often a specific cross-link was identified (1/3, 2/3 or 3/3 experiments). Intraprotein cross-links in BSA are shown in sky blue; interprotein (homotypic) cross-links occurred due to dimerization of BSA and are shown in red. In the schematic representation of BSA, α-helices are colored Naples yellow, intrinsically disordered regions (IDRs) are shown in coral red.

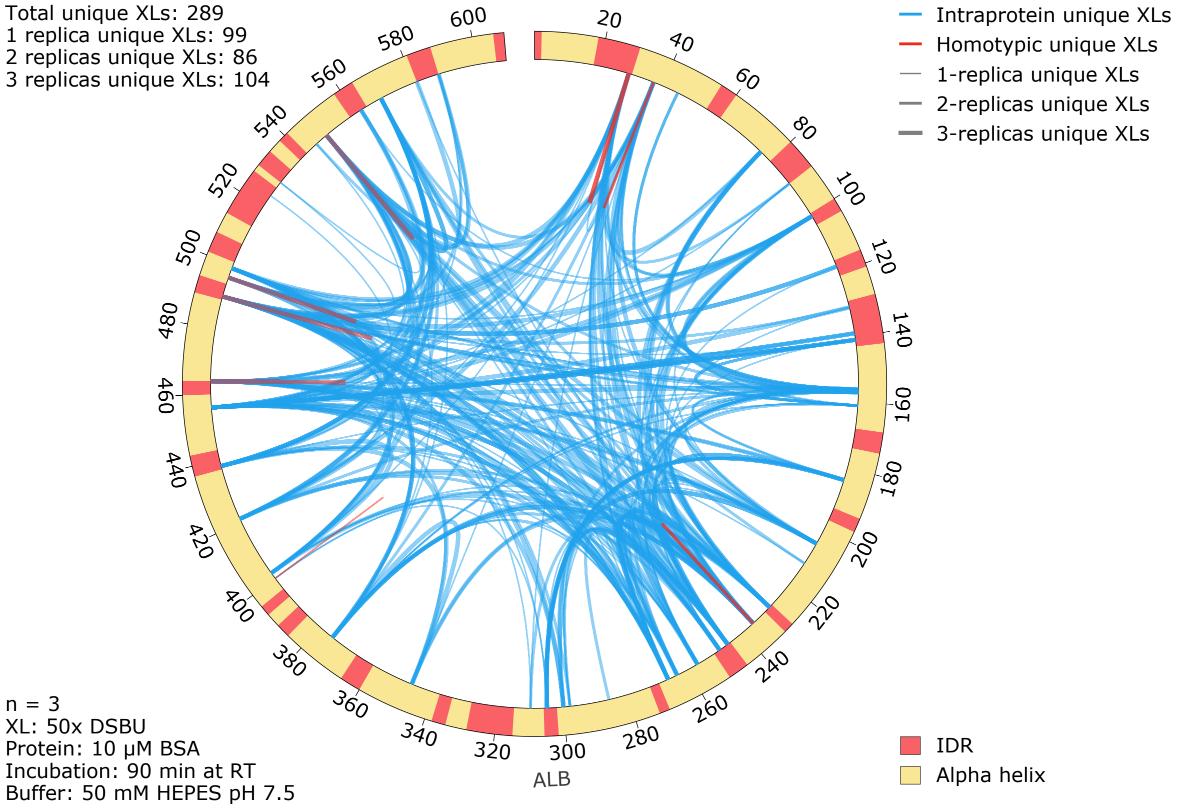

Figure S32. Circos plot of DSBU cross-links (50-fold molar excess) identified for BSA (90 min, room temperature, no SDS). Cross-links were identified using MeroX. Thickness of lines represents how often a specific cross-link was identified (1/3, 2/3 or 3/3 experiments). Intraprotein cross-links in BSA are shown in sky blue; interprotein (homotypic) cross-links occurred due to dimerization of BSA and are shown in red. In the schematic representation of BSA, α-helices are colored Naples yellow, intrinsically disordered regions (IDRs) are shown in coral red.

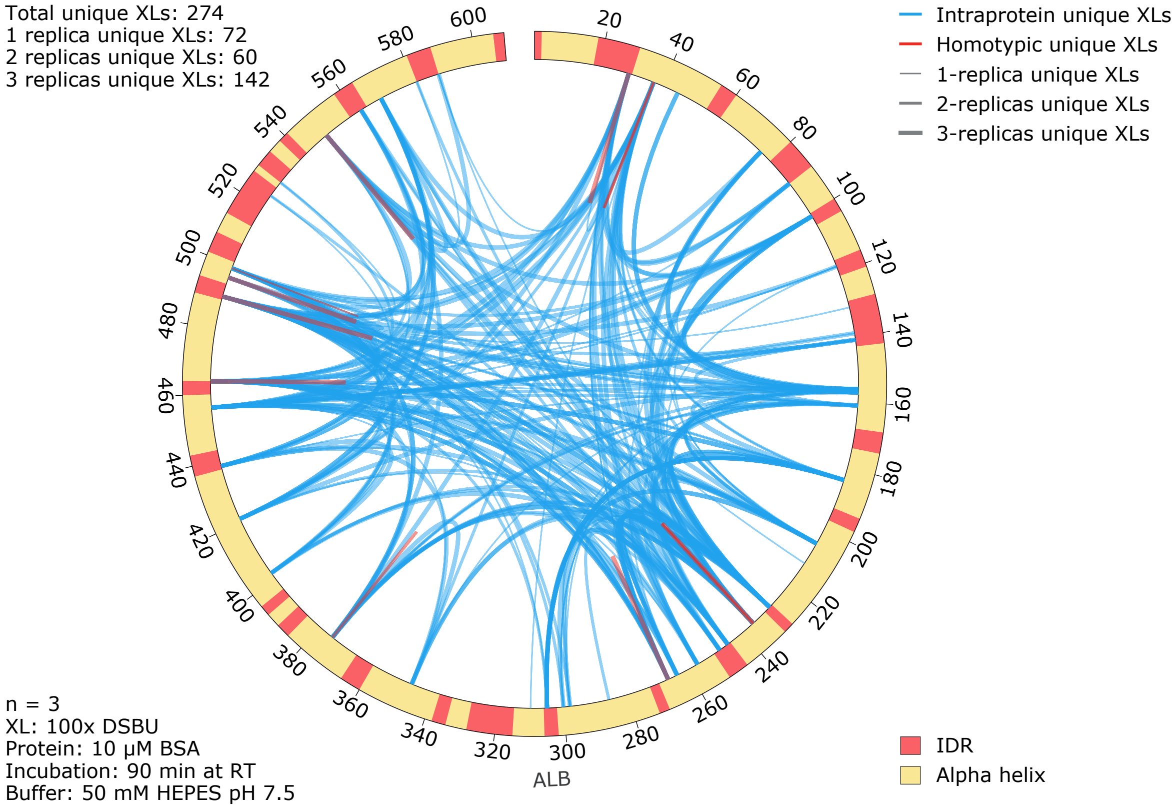

Figure S33. Circos plot of DSBU cross-links (100-fold molar excess) identified for BSA (90 min, room temperature, no SDS). Cross-links were identified using MeroX. Thickness of lines represents how often a specific cross-link was identified (1/3, 2/3 or 3/3 experiments). Intraprotein cross-links in BSA are shown in sky blue; interprotein (homotypic) cross-links occurred due to dimerization of BSA and are shown in red. In the schematic representation of BSA, α-helices are colored Naples yellow, intrinsically disordered regions (IDRs) are shown in coral red.

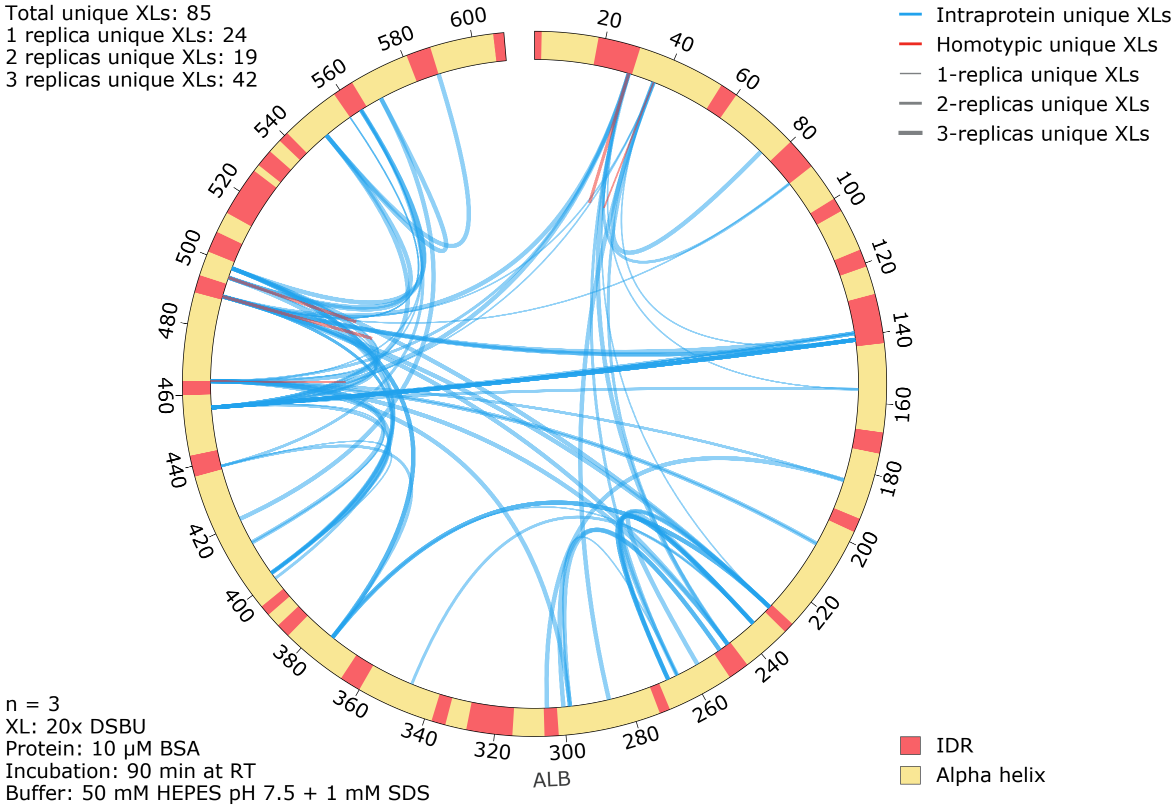

Figure S34. Circos plot of DSBU cross-links (20-fold molar excess) identified for BSA (90 min, room temperature, 1 mM SDS). Cross-links were identified using MeroX. Thickness of lines represents how often a specific cross-link was identified (1/3, 2/3 or 3/3 experiments). Intraprotein cross-links in BSA are shown in sky blue; interprotein (homotypic) cross-links occurred due to dimerization of BSA and are shown in red. In the schematic representation of BSA, α-helices are colored Naples yellow, intrinsically disordered regions (IDRs) are shown in coral red.

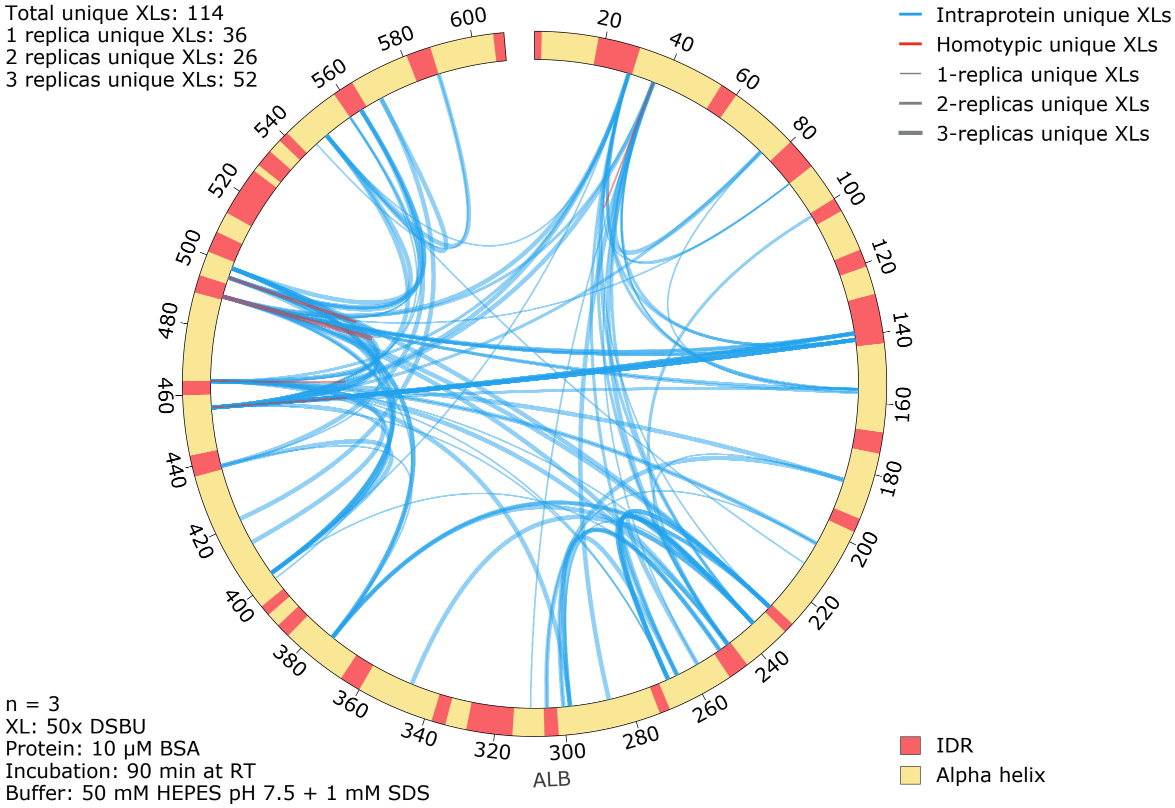

Figure S35. Circos plot of DSBU cross-links (50-fold molar excess) identified for BSA (90 min, room temperature, 1 mM SDS). Cross-links were identified using MeroX. Thickness of lines represents how often a specific cross-link was identified (1/3, 2/3 or 3/3 experiments). Intraprotein cross-links in BSA are shown in sky blue; interprotein (homotypic) cross-links occurred due to dimerization of BSA and are shown in red. In the schematic representation of BSA, α-helices are colored Naples yellow, intrinsically disordered regions (IDRs) are shown in coral red.

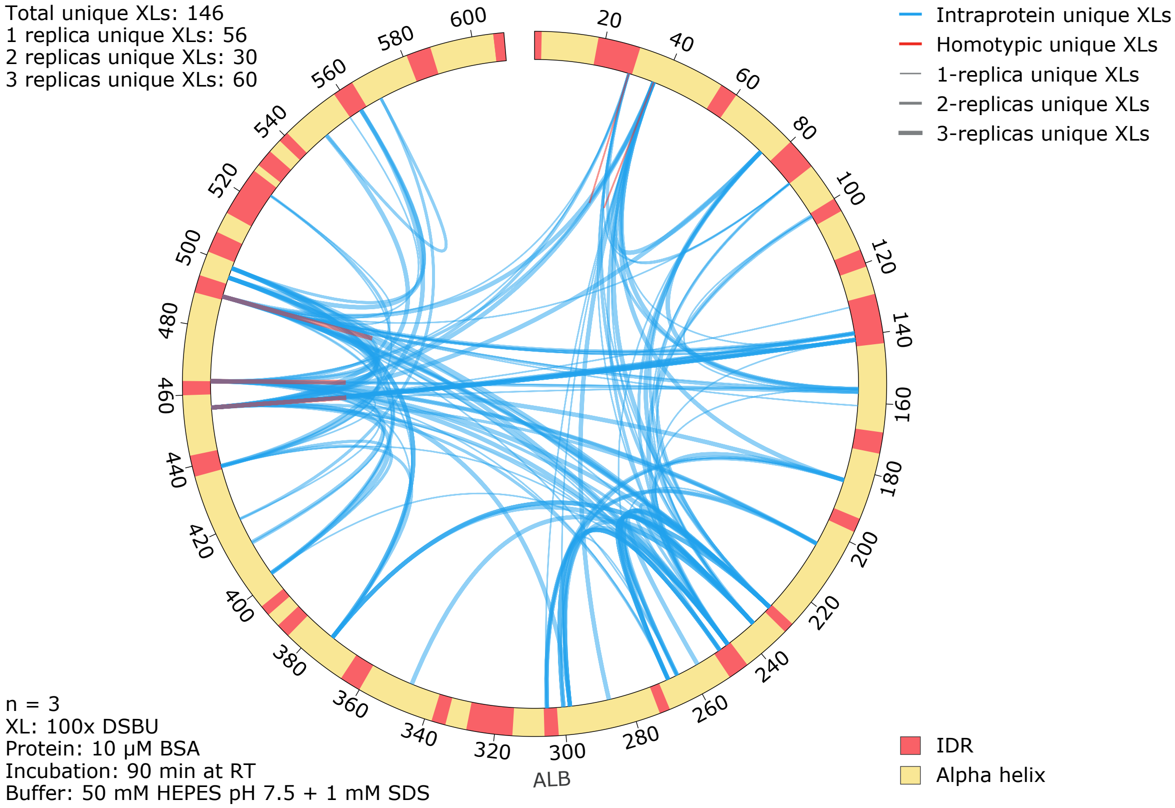

Figure S56. Circos plot of DSBU cross-links (100-fold molar excess) identified for BSA (90 min, room temperature, 1 mM SDS). Cross-links were identified using MeroX. Thickness of lines represents how often a specific cross-link was identified (1/3, 2/3 or 3/3 experiments). Intraprotein cross-links in BSA are shown in sky blue; interprotein (homotypic) cross-links occurred due to dimerization of BSA and are shown in red. In the schematic representation of BSA, α-helices are colored Naples yellow, , intrinsically disordered regions (IDRs) are shown in coral red.

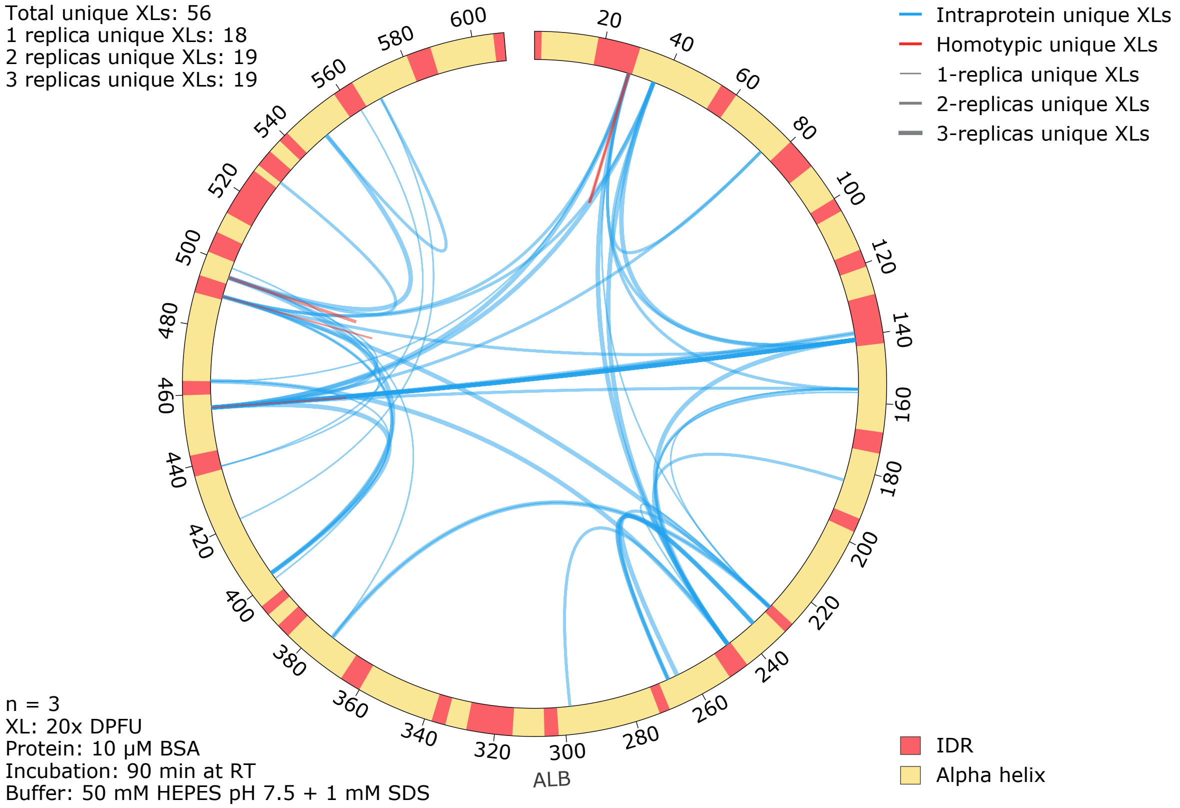

Figure S37. Circos plot of DPFU cross-links (20-fold molar excess) identified for BSA (90 min, room temperature, 1 mM SDS). Cross-links were identified using MeroX. Thickness of lines represents how often a specific cross-link was identified (1/3, 2/3 or 3/3 experiments). Intraprotein cross-links in BSA are shown in sky blue; interprotein (homotypic) cross-links occurred due to dimerization of BSA and are shown in red. In the schematic representation of BSA, α-helices are colored Naples yellow, intrinsically disordered regions (IDRs) are shown in coral red.

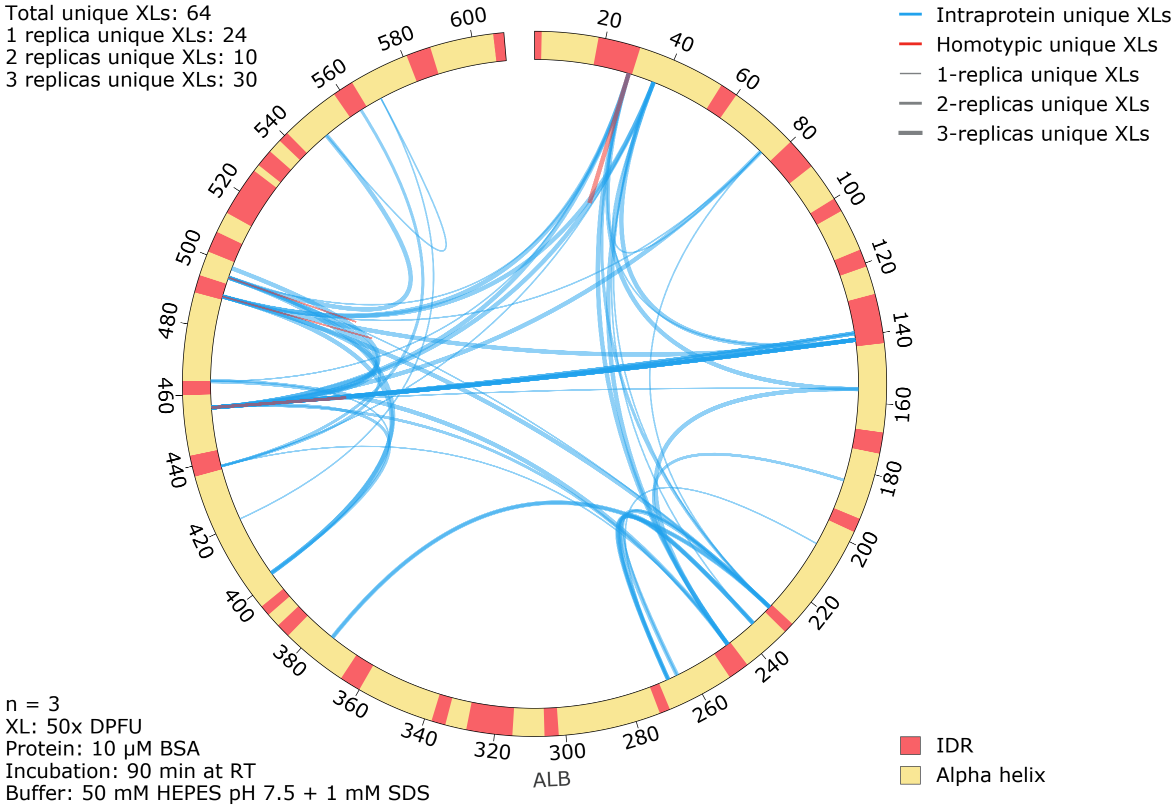

Figure S38. Circos plot of DPFU cross-links (50-fold molar excess) identified for BSA (90 min, room temperature, 1 mM SDS). Cross-links were identified using MeroX. Thickness of lines represents how often a specific cross-link was identified (1/3, 2/3 or 3/3 experiments). Intraprotein cross-links in BSA are shown in sky blue; interprotein (homotypic) cross-links occurred due to dimerization of BSA and are shown in red. In the schematic representation of BSA, α-helices are colored Naples yellow, intrinsically disordered regions (IDRs) are shown in coral red.

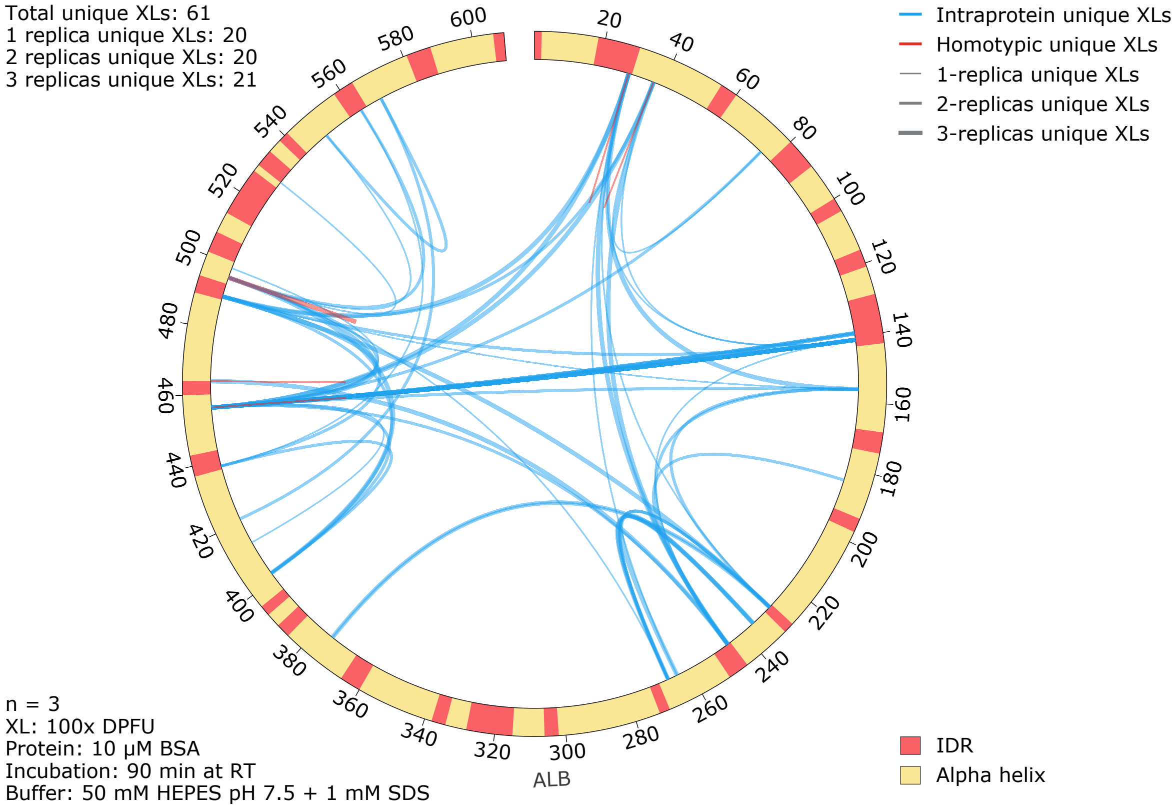

Figure S39. Circos plot of DPFU cross-links (100-fold molar excess) identified for BSA (90 min, room temperature, 1 mM SDS). Cross-links were identified using MeroX. Thickness of lines represents how often a specific cross-link was identified (1/3, 2/3 or 3/3 experiments). Intraprotein cross-links in BSA are shown in sky blue; interprotein (homotypic) cross-links occurred due to dimerization of BSA and are shown in red. In the schematic representation of BSA, α-helices are colored Naples yellow, intrinsically disordered regions (IDRs) are shown in coral red.

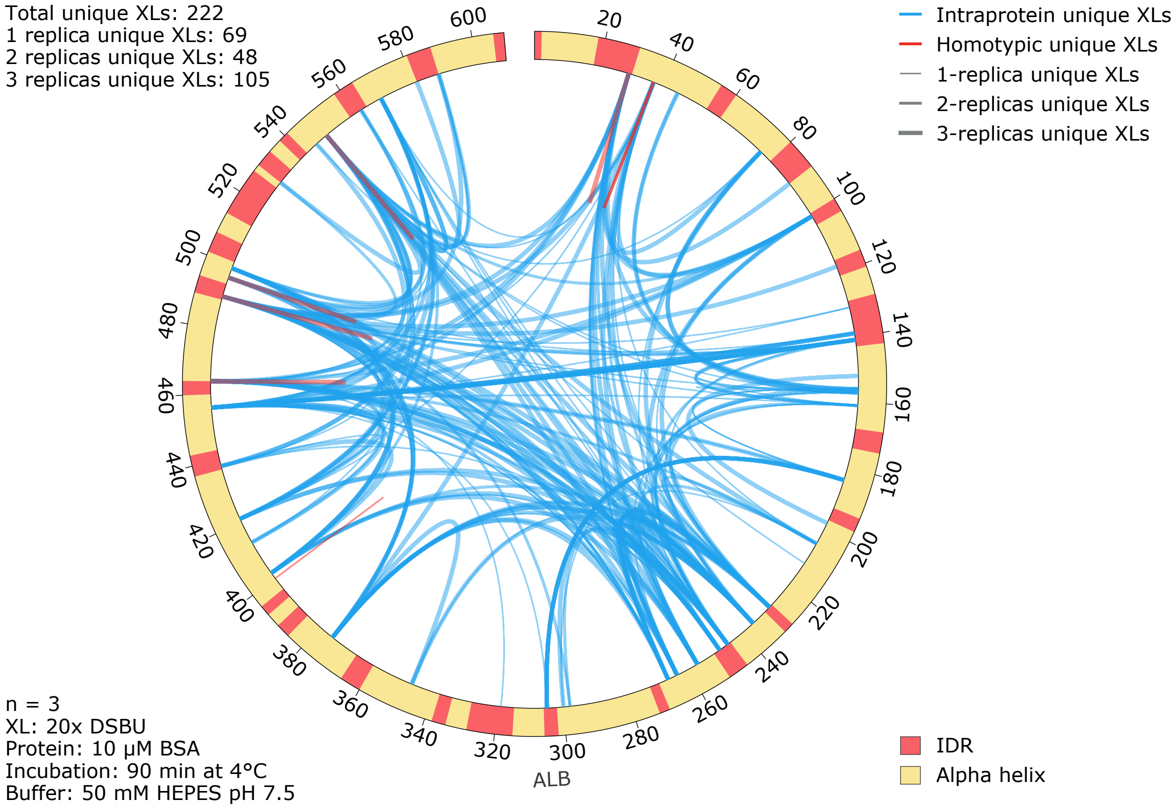

Figure S40. Circos plot of DSBU cross-links (20-fold molar excess) identified for BSA (90 min, 4°C, no SDS). Cross-links were identified using MeroX. Thickness of lines represents how often a specific cross-link was identified (1/3, 2/3 or 3/3 experiments). Intraprotein cross-links in BSA are shown in sky blue; interprotein (homotypic) cross-links occurred due to dimerization of BSA and are shown in red. In the schematic representation of BSA, α-helices are colored Naples yellow, intrinsically disordered regions (IDRs) are shown in coral red.

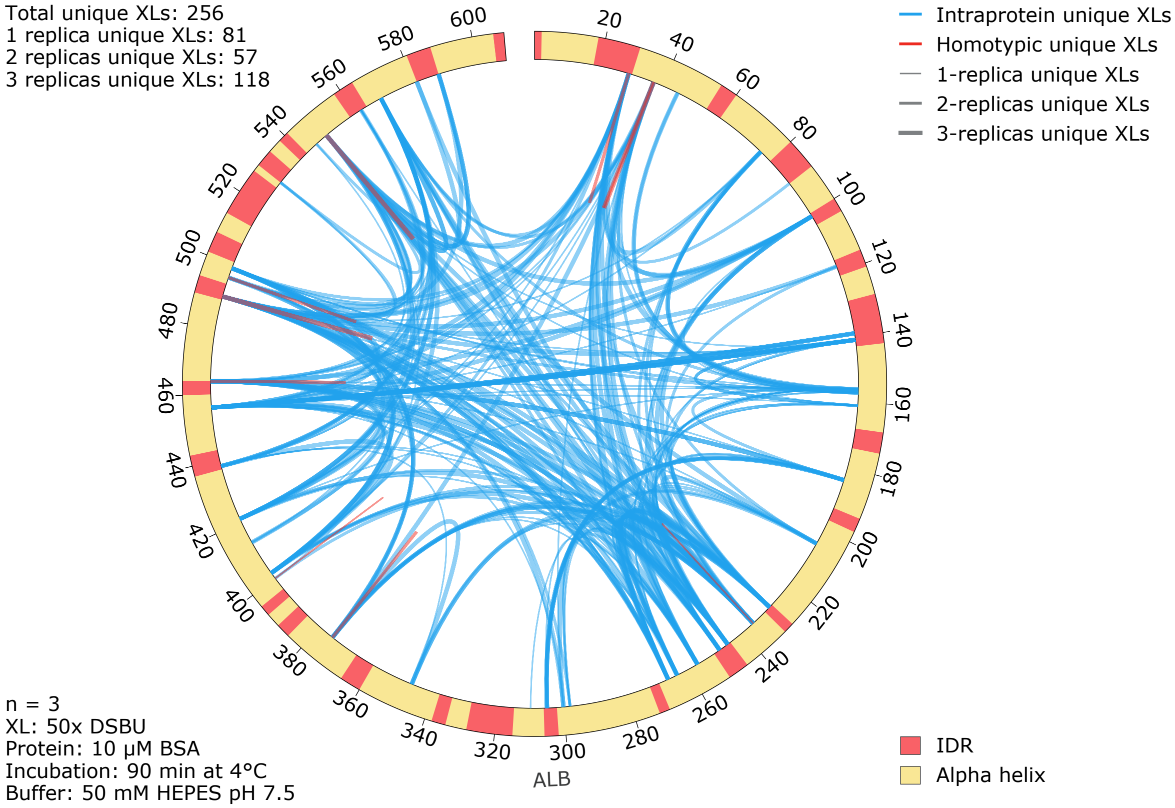

Figure S41. Circos plot of DSBU cross-links (50-fold molar excess) identified for BSA (90 min, 4°C, no SDS). Cross-links were identified using MeroX. Thickness of lines represents how often a specific cross-link was identified (1/3, 2/3 or 3/3 experiments). Intraprotein cross-links in BSA are shown in sky blue; interprotein (homotypic) cross-links occurred due to dimerization of BSA and are shown in red. In the schematic representation of BSA, α-helices are colored Naples yellow, intrinsically disordered regions (IDRs) are shown in coral red.

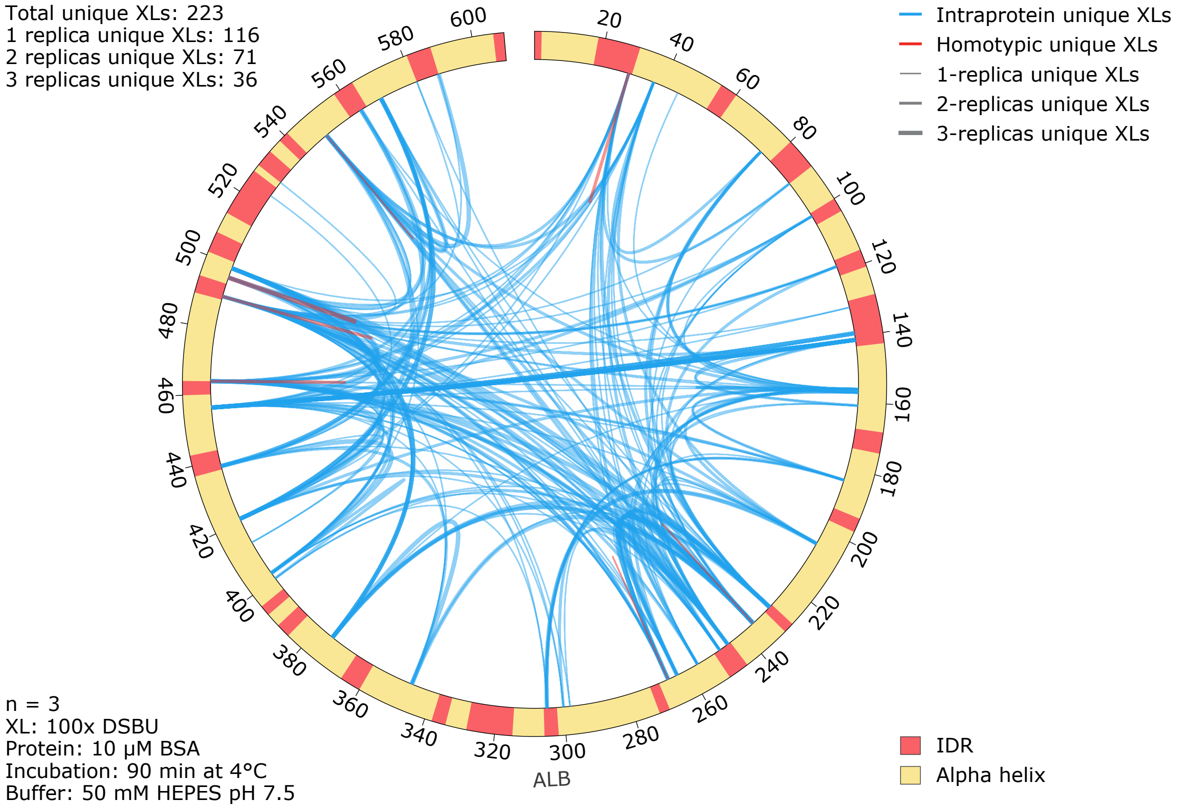

Figure S42. Circos plot of DSBU cross-links (100-fold molar excess) identified for BSA (90 min, 4°C, no SDS). Cross-links were identified using MeroX. Thickness of lines represents how often a specific cross-link was identified (1/3, 2/3 or 3/3 experiments). Intraprotein cross-links in BSA are shown in sky blue; interprotein (homotypic) cross-links occurred due to dimerization of BSA and are shown in red. In the schematic representation of BSA, α-helices are colored Naples yellow, intrinsically disordered regions (IDRs) are shown in coral red.

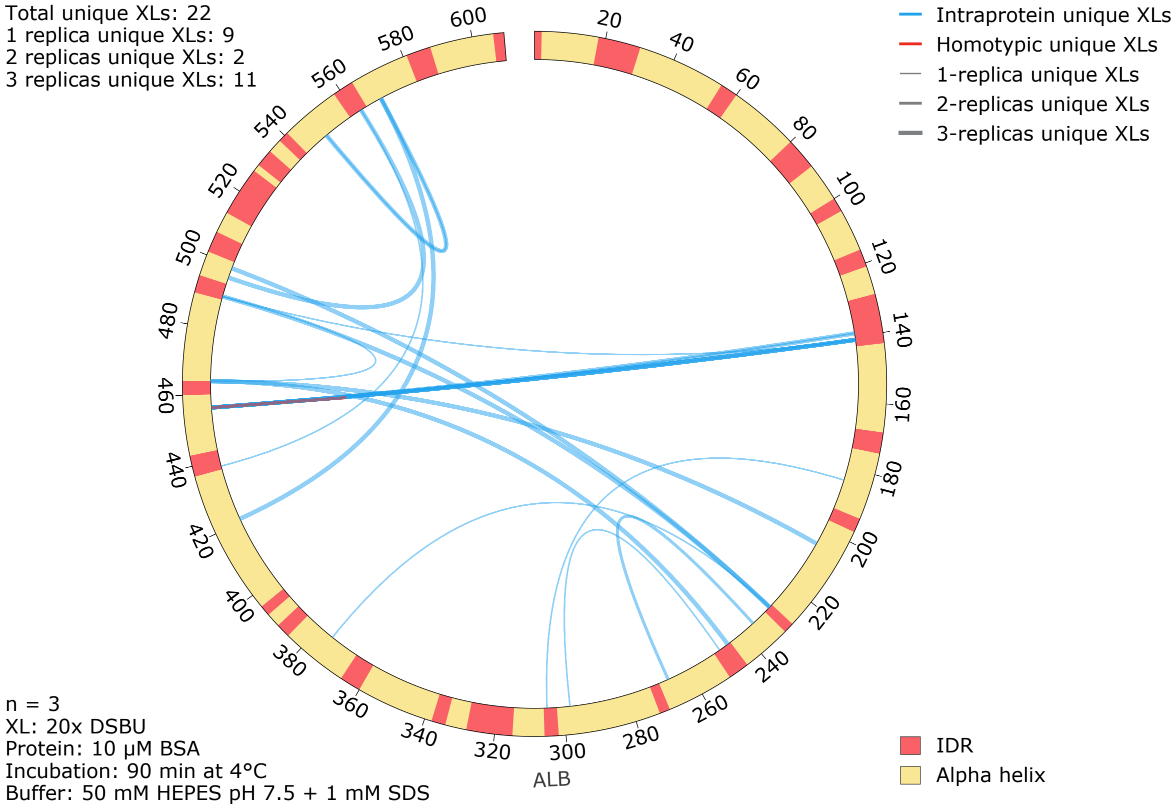

Figure S43. Circos plot of DSBU cross-links (20-fold molar excess) identified for BSA (90 min, 4°C, 1 mM SDS). Cross-links were identified using MeroX. Thickness of lines represents how often a specific cross-link was identified (1/3, 2/3 or 3/3 experiments). Intraprotein cross-links in BSA are shown in sky blue; interprotein (homotypic) cross-links occurred due to dimerization of BSA and are shown in red. In the schematic representation of BSA, α-helices are colored Naples yellow, intrinsically disordered regions (IDRs) are shown in coral red.

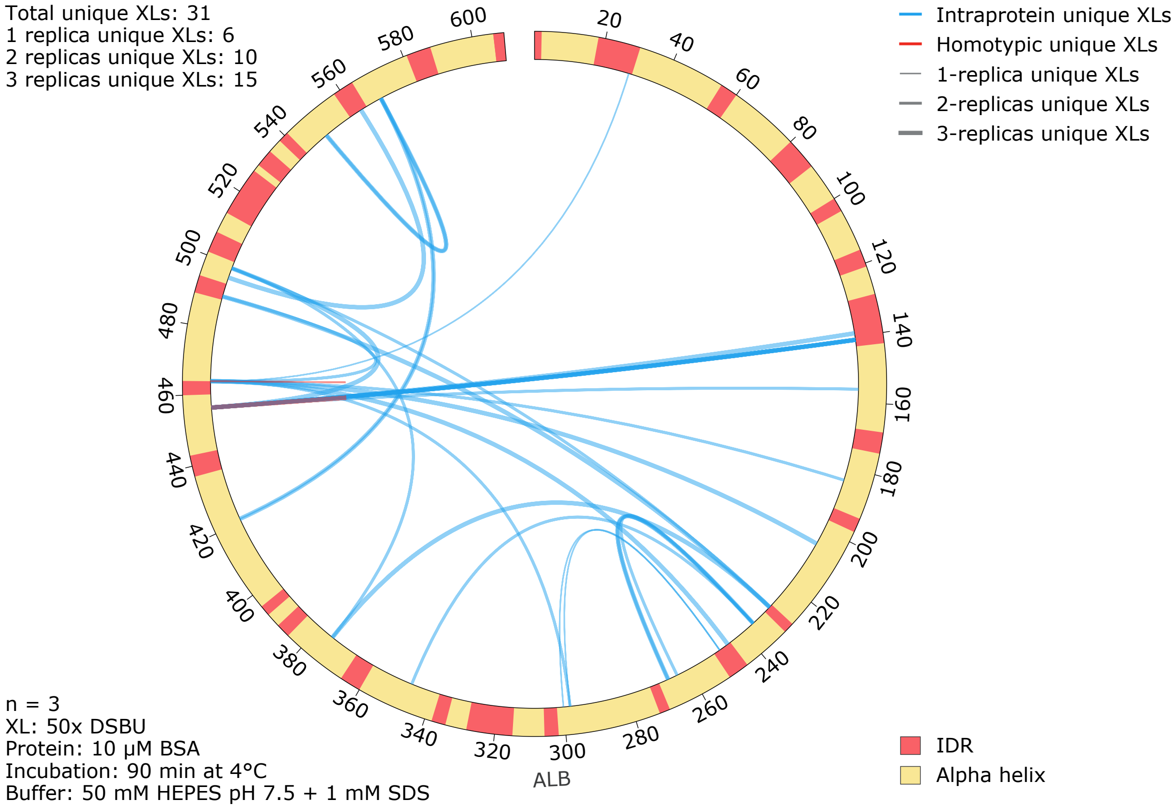

Figure S44. Circos plot of DSBU cross-links (50-fold molar excess) identified for BSA (90 min, 4°C, 1 mM SDS). Cross-links were identified using MeroX. Thickness of lines represents how often a specific cross-link was identified (1/3, 2/3 or 3/3 experiments). Intraprotein cross-links in BSA are shown in sky blue; interprotein (homotypic) cross-links occurred due to dimerization of BSA and are shown in red. In the schematic representation of BSA, α-helices are colored Naples yellow, intrinsically disordered regions (IDRs) are shown in coral red.

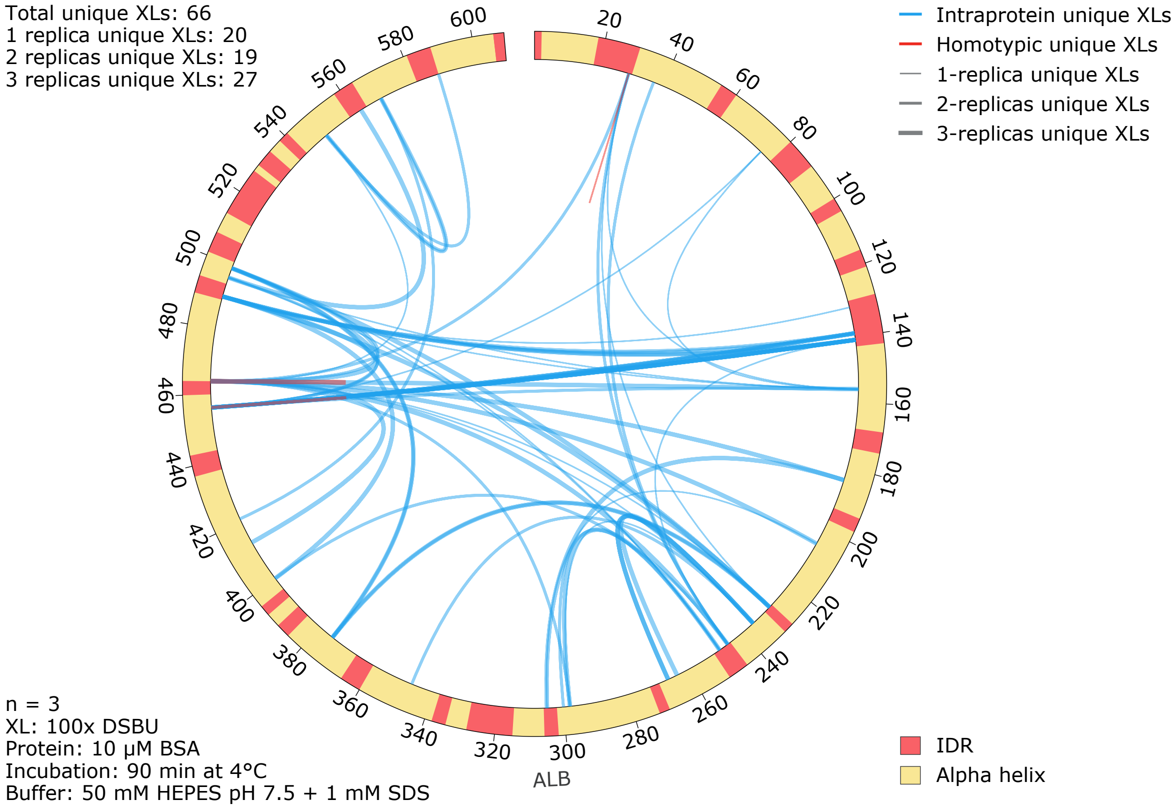

Figure S45. Circos plot of DSBU cross-links (100-fold molar excess) identified for BSA (90 min, 4°C, 1 mM SDS). Cross-links were identified using MeroX. Thickness of lines represents how often a specific cross-link was identified (1/3, 2/3 or 3/3 experiments). Intraprotein cross-links in BSA are shown in sky blue; interprotein (homotypic) cross-links occurred due to dimerization of BSA and are shown in red. In the schematic representation of BSA, α-helices are colored Naples yellow, intrinsically disordered regions (IDRs) are shown in coral red.

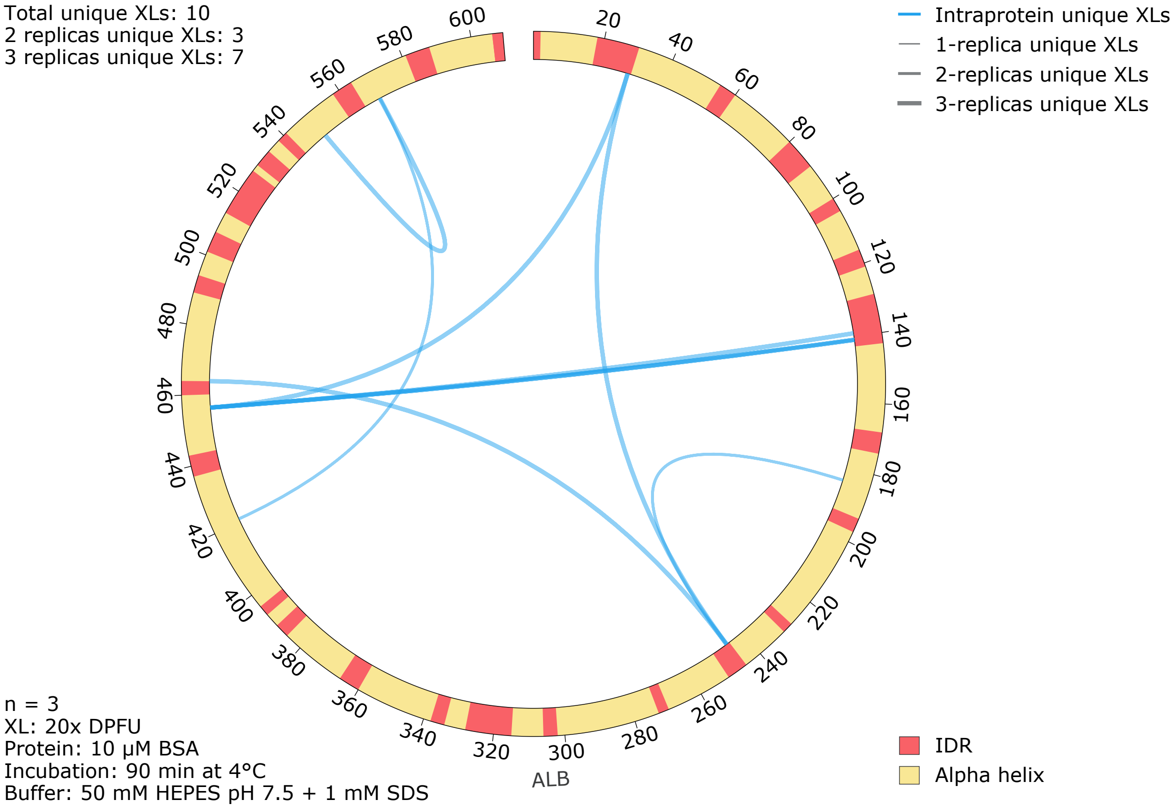

Figure S46. Circos plot of DPFU cross-links (20-fold molar excess) identified for BSA (90 min, 4°C, 1 mM SDS). Cross-links were identified using MeroX. Thickness of lines represents how often a specific cross-link was identified (1/3, 2/3 or 3/3 experiments). Intraprotein cross-links in BSA are shown in sky blue; interprotein (homotypic) cross-links occurred due to dimerization of BSA and are shown in red. In the schematic representation of BSA, α-helices are colored Naples yellow, intrinsically disordered regions (IDRs) are shown in coral red.

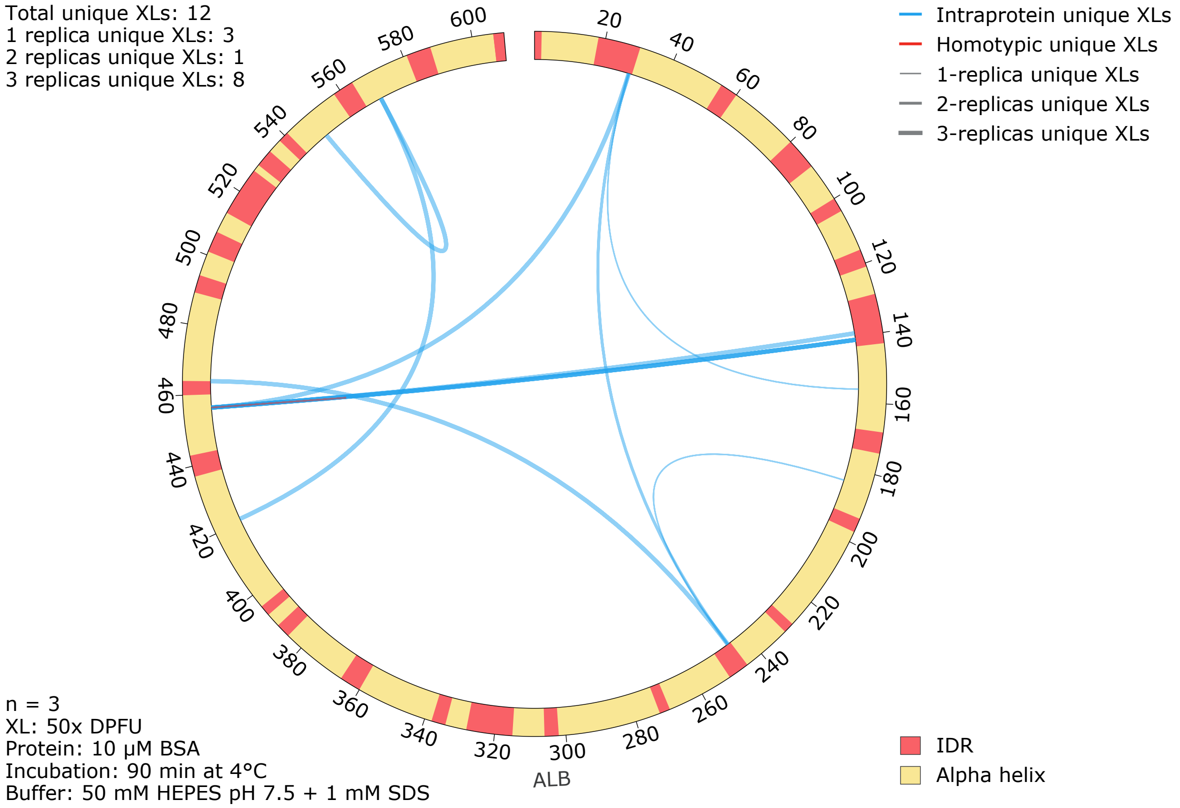

Figure S47. Circos plot of DPFU cross-links (50-fold molar excess) identified for BSA (90 min, 4°C, 1 mM SDS). Cross-links were identified using MeroX. Thickness of lines represents how often a specific cross-link was identified (1/3, 2/3 or 3/3 experiments). Intraprotein cross-links in BSA are shown in sky blue; interprotein (homotypic) cross-links occurred due to dimerization of BSA and are shown in red. In the schematic representation of BSA, α-helices are colored Naples yellow, intrinsically disordered regions (IDRs) are shown in coral red.

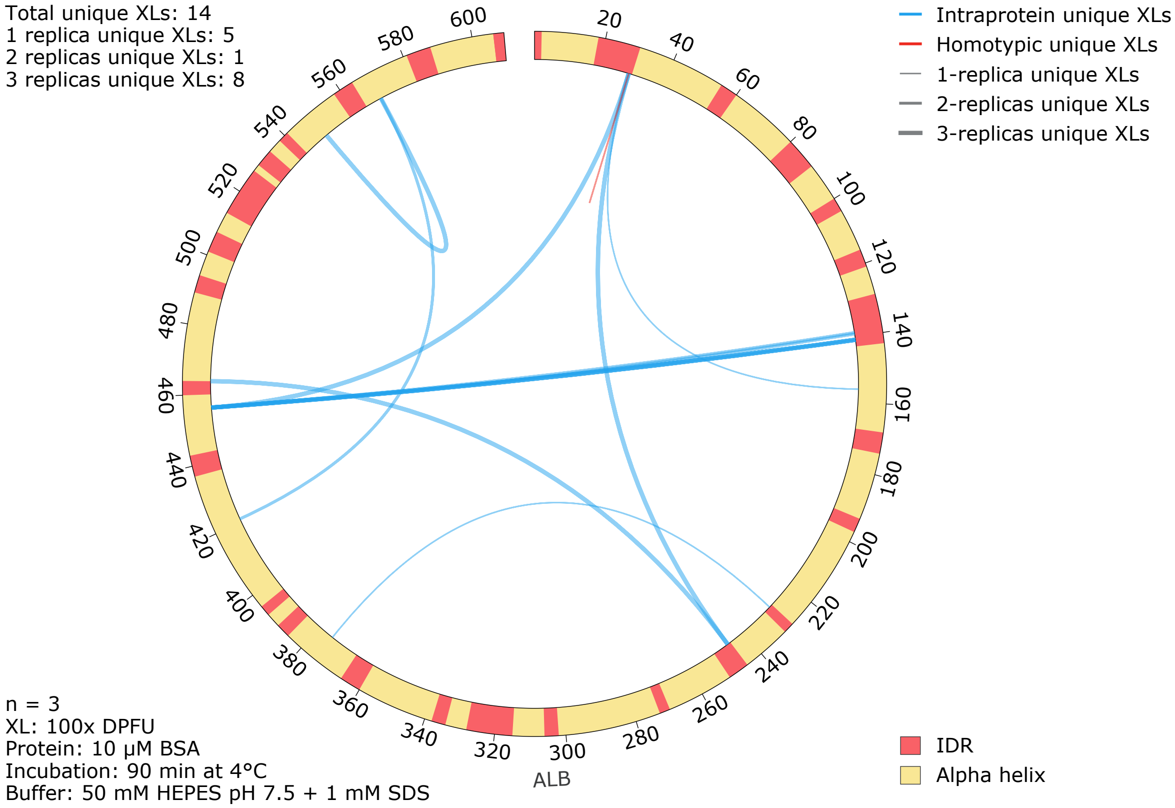

Figure S48. Circos plot of DPFU cross-links (100-fold molar excess) identified for BSA (90 min, 4°C, 1 mM SDS). Cross-links were identified using MeroX. Thickness of lines represents how often a specific cross-link was identified (1/3, 2/3 or 3/3 experiments). Intraprotein cross-links in BSA are shown in sky blue; interprotein (homotypic) cross-links occurred due to dimerization of BSA and are shown in red. In the schematic representation of BSA, α-helices are colored Naples yellow, intrinsically disordered regions (IDRs) are shown in coral red.

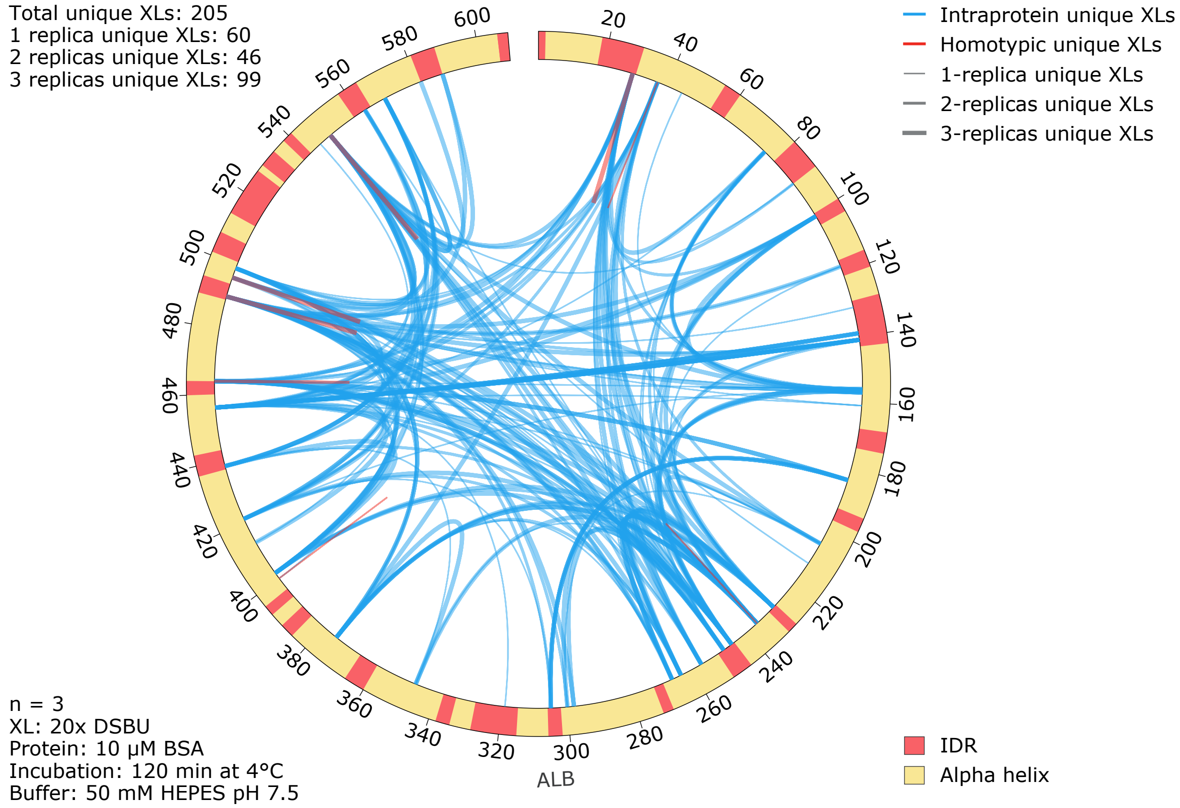

Figure S49. Circos plot of DSBU cross-links (20-fold molar excess) identified for BSA (120 min, 4°C, no SDS). Cross-links were identified using MeroX. Thickness of lines represents how often a specific cross-link was identified (1/3, 2/3 or 3/3 experiments). Intraprotein cross-links in BSA are shown in sky blue; interprotein (homotypic) cross-links occurred due to dimerization of BSA and are shown in red. In the schematic representation of BSA, α-helices are colored Naples yellow, intrinsically disordered regions (IDRs) are shown in coral red.

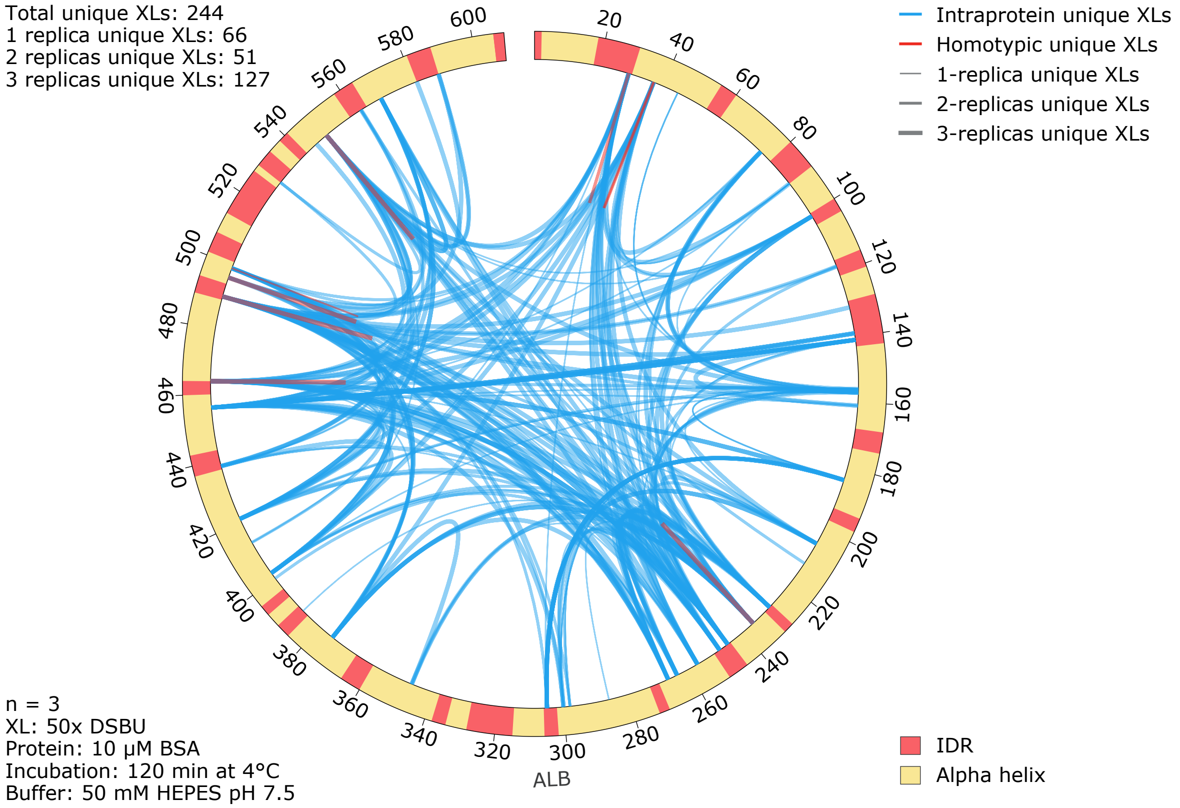

Figure S50. Circos plot of DSBU cross-links (50-fold molar excess) identified for BSA (120 min, 4°C, no SDS). Cross-links were identified using MeroX. Thickness of lines represents how often a specific cross-link was identified (1/3, 2/3 or 3/3 experiments). Intraprotein cross-links in BSA are shown in sky blue; interprotein (homotypic) cross-links occurred due to dimerization of BSA and are shown in red. In the schematic representation of BSA, α-helices are colored Naples yellow, intrinsically disordered regions (IDRs) are shown in coral red.

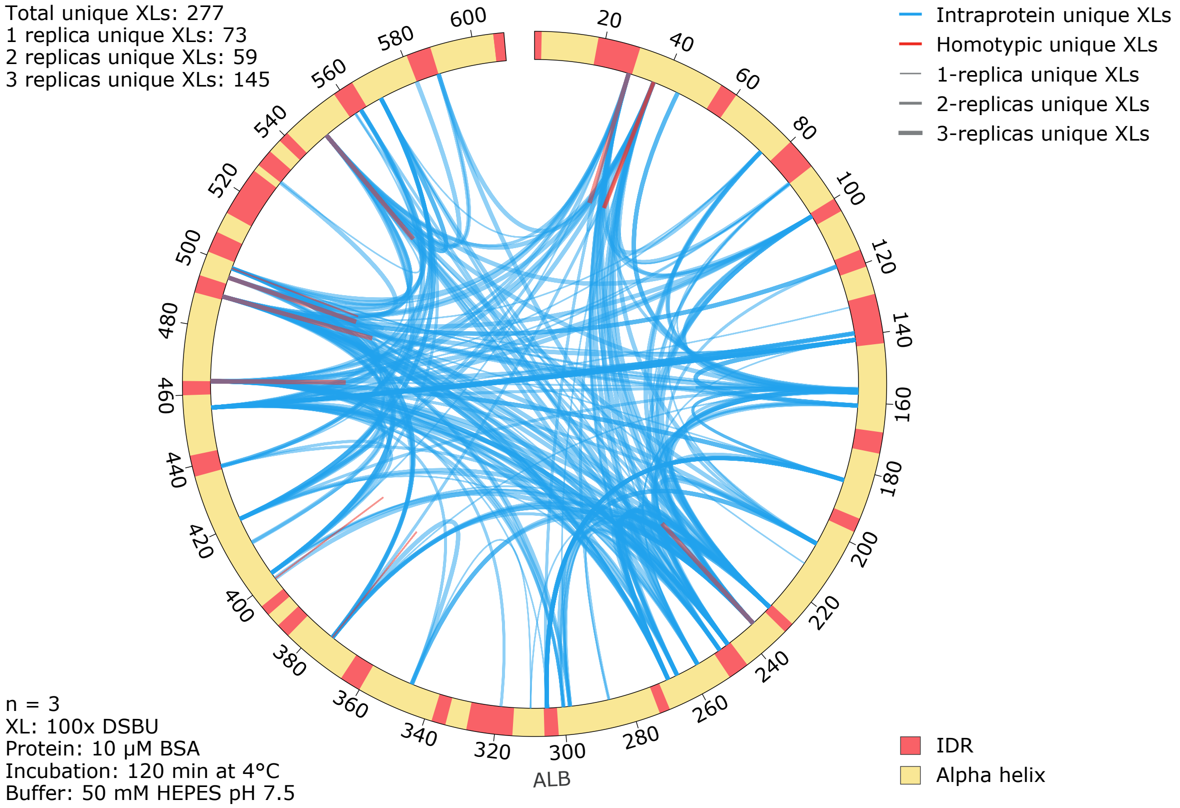

Figure S51. Circos plot of DSBU cross-links (100-fold molar excess) identified for BSA (120 min, 4°C, no SDS). Cross-links were identified using MeroX. Thickness of lines represents how often a specific cross-link was identified (1/3, 2/3 or 3/3 experiments). Intraprotein cross-links in BSA are shown in sky blue; interprotein (homotypic) cross-links occurred due to dimerization of BSA and are shown in red. In the schematic representation of BSA, α-helices are colored Naples yellow, intrinsically disordered regions (IDRs) are shown in coral red.

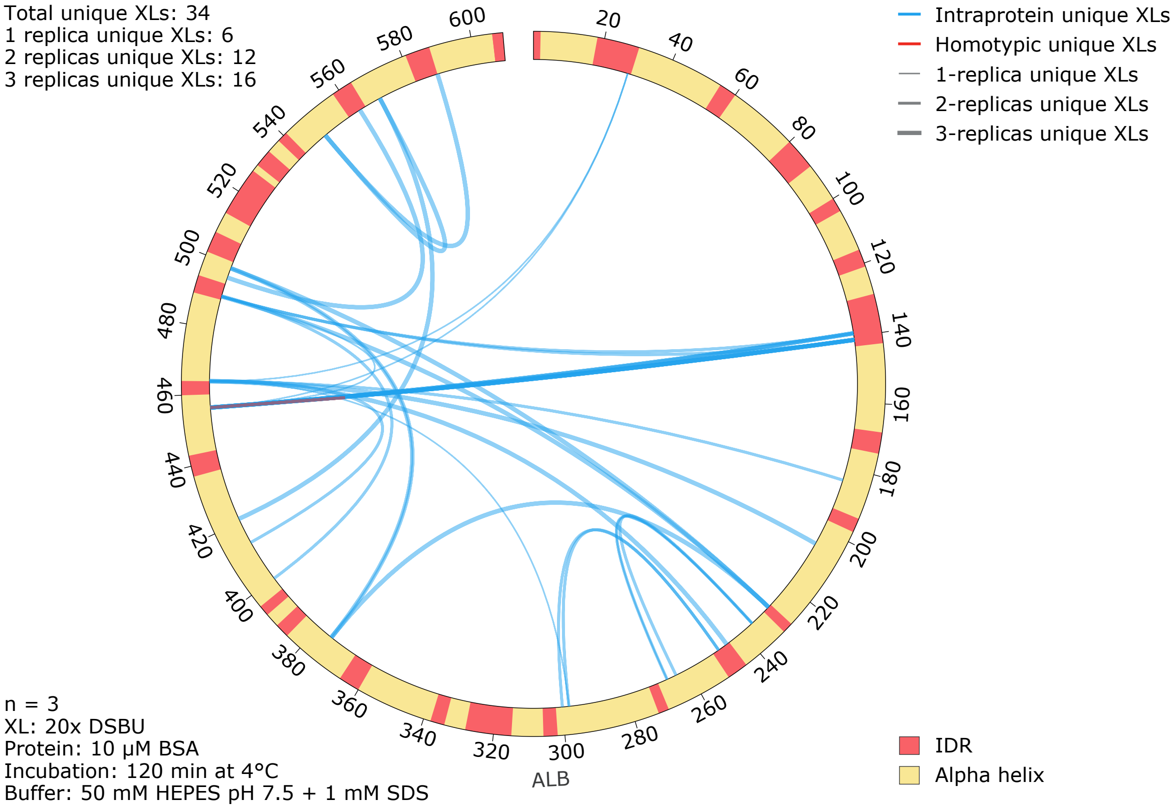

Figure S52. Circos plot of DSBU cross-links (20-fold molar excess) identified for BSA (120 min, 4°C, 1 mM SDS). Cross-links were identified using MeroX. Thickness of lines represents how often a specific cross-link was identified (1/3, 2/3 or 3/3 experiments). Intraprotein cross-links in BSA are shown in sky blue; interprotein (homotypic) cross-links occurred due to dimerization of BSA and are shown in red. In the schematic representation of BSA, α-helices are colored Naples yellow, intrinsically disordered regions (IDRs) are shown in coral red.

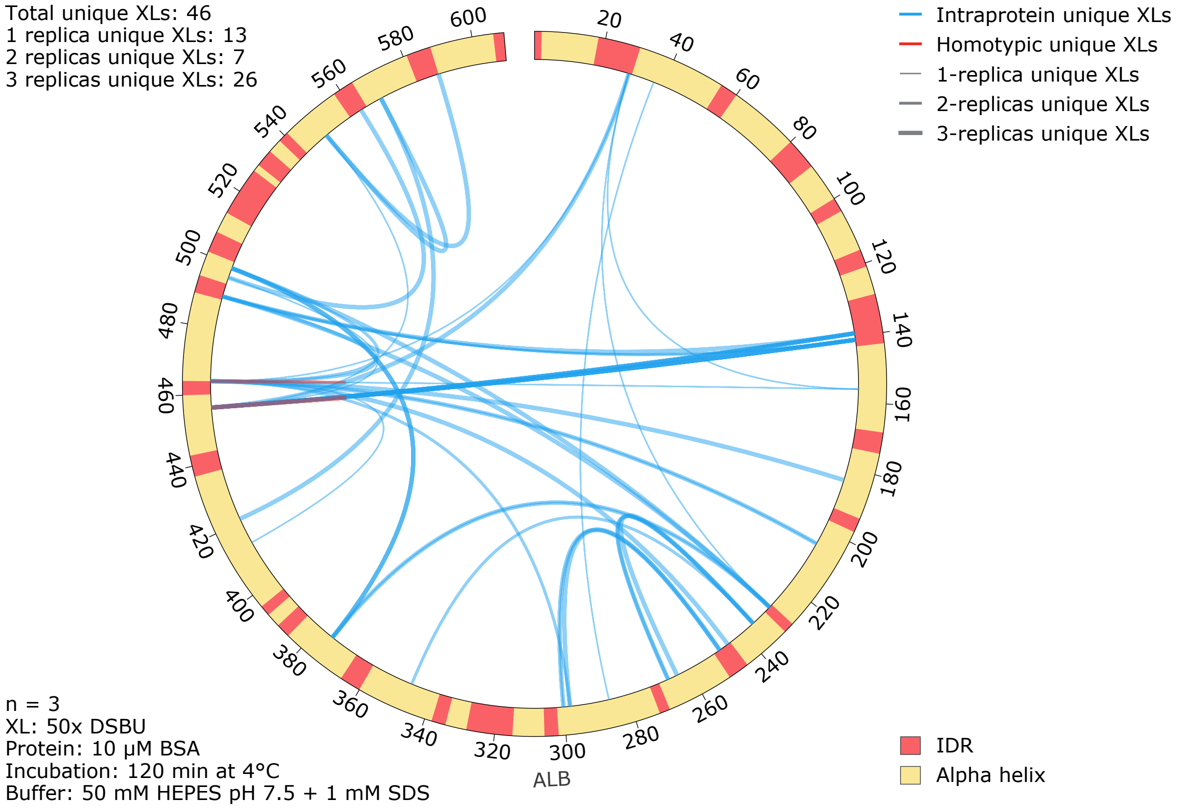

Figure S53. Circos plot of DSBU cross-links (50-fold molar excess) identified for BSA (120 min, 4°C, 1 mM SDS). Cross-links were identified using MeroX. Thickness of lines represents how often a specific cross-link was identified (1/3, 2/3 or 3/3 experiments). Intraprotein cross-links in BSA are shown in sky blue; interprotein (homotypic) cross-links occurred due to dimerization of BSA and are shown in red. In the schematic representation of BSA, α-helices are colored Naples yellow, intrinsically disordered regions (IDRs) are shown in coral red.

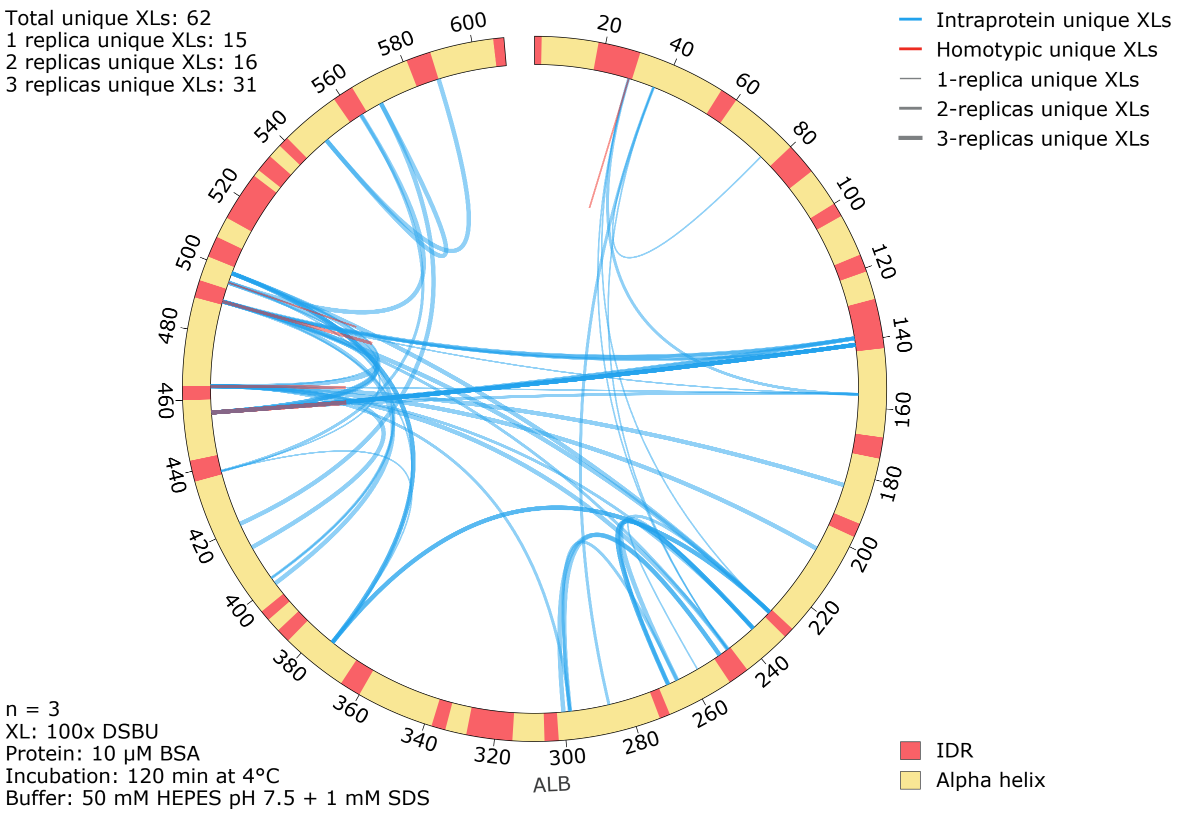

Figure S54. Circos plot of DSBU cross-links (100-fold molar excess) identified for BSA (120 min, 4°C, 1 mM SDS). Cross-links were identified using MeroX. Thickness of lines represents how often a specific cross-link was identified (1/3, 2/3 or 3/3 experiments). Intraprotein cross-links in BSA are shown in sky blue; interprotein (homotypic) cross-links occurred due to dimerization of BSA and are shown in red. In the schematic representation of BSA, α-helices are colored Naples yellow, intrinsically disordered regions (IDRs) are shown in coral red.

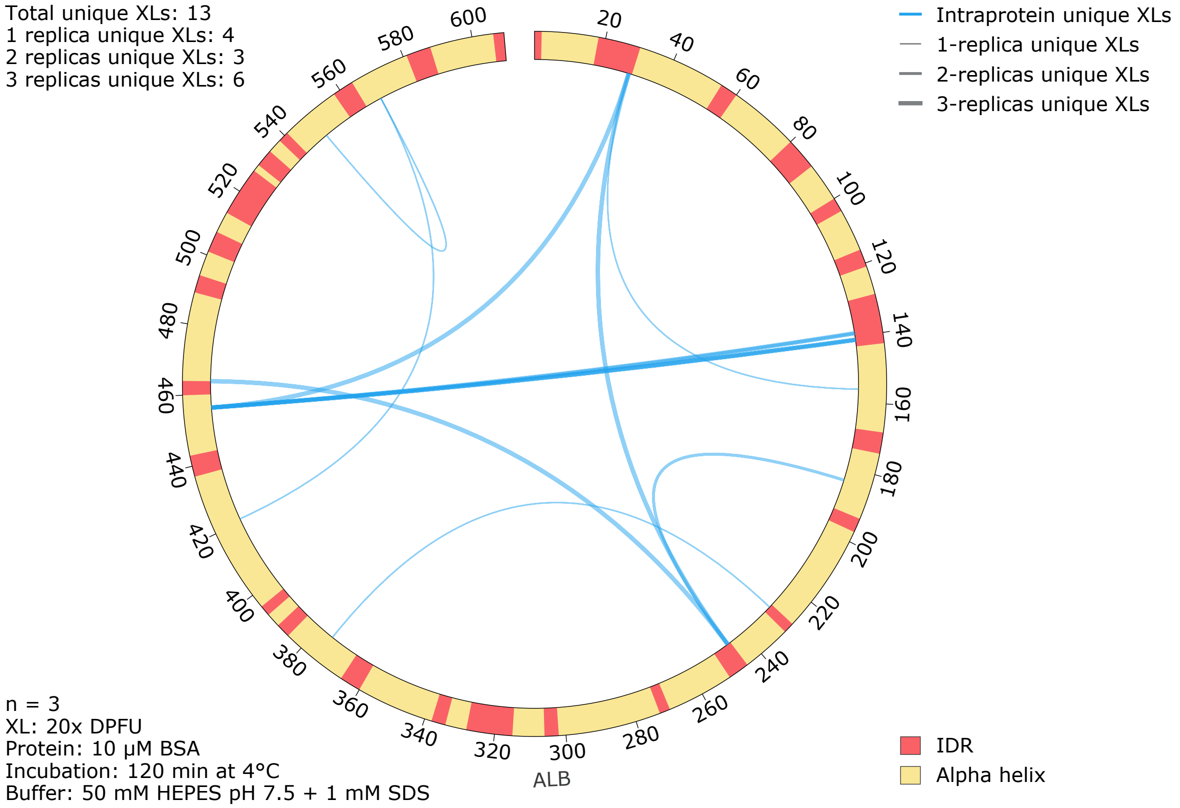

Figure S55. Circos plot of DPFU cross-links (20-fold molar excess) identified for BSA (120 min, 4°C, 1 mM SDS). Cross-links were identified using MeroX. Thickness of lines represents how often a specific cross-link was identified (1/3, 2/3 or 3/3 experiments). Intraprotein cross-links in BSA are shown in sky blue; interprotein (homotypic) cross-links occurred due to dimerization of BSA and are shown in red. In the schematic representation of BSA, α-helices are colored Naples yellow, intrinsically disordered regions (IDRs) are shown in coral red.

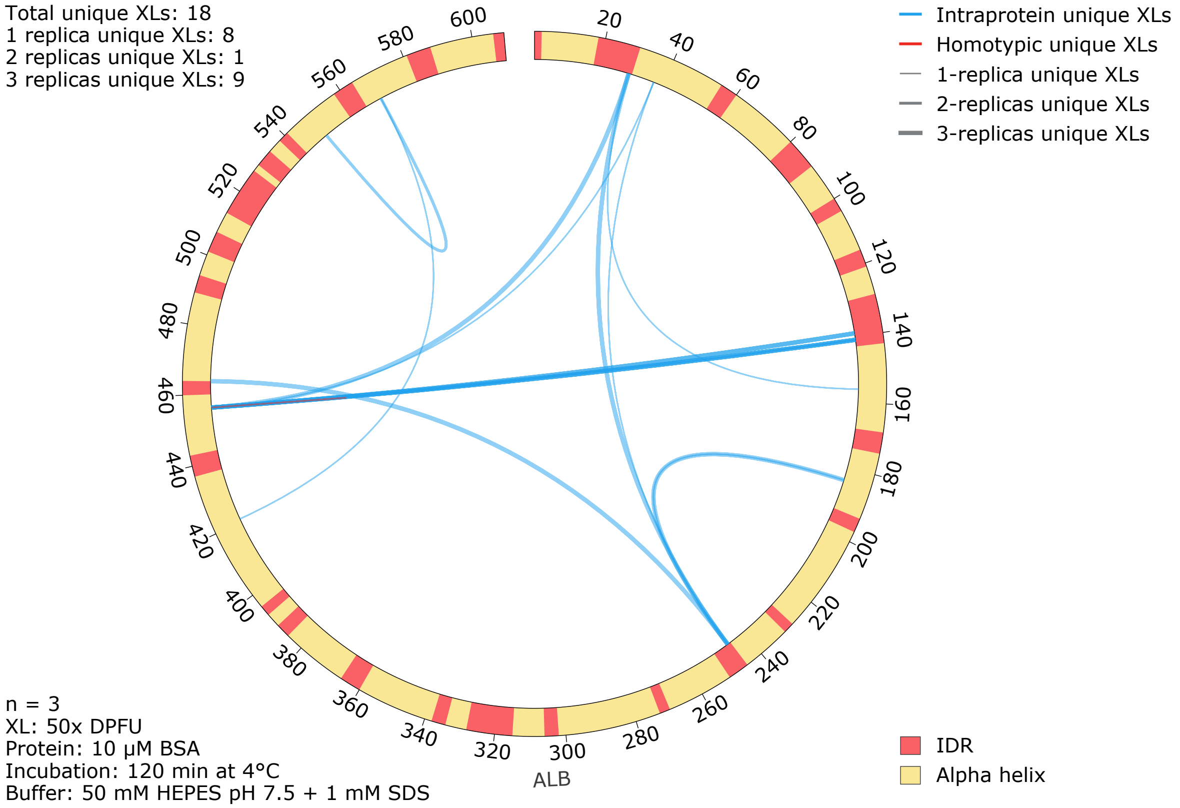

Figure S56. Circos plot of DPFU cross-links (50-fold molar excess) identified for BSA (120 min, 4°C, 1 mM SDS). Cross-links were identified using MeroX. Thickness of lines represents how often a specific cross-link was identified (1/3, 2/3 or 3/3 experiments). Intraprotein cross-links in BSA are shown in sky blue; interprotein (homotypic) cross-links occurred due to dimerization of BSA and are shown in red. In the schematic representation of BSA, α-helices are colored Naples yellow, intrinsically disordered regions (IDRs) are shown in coral red.

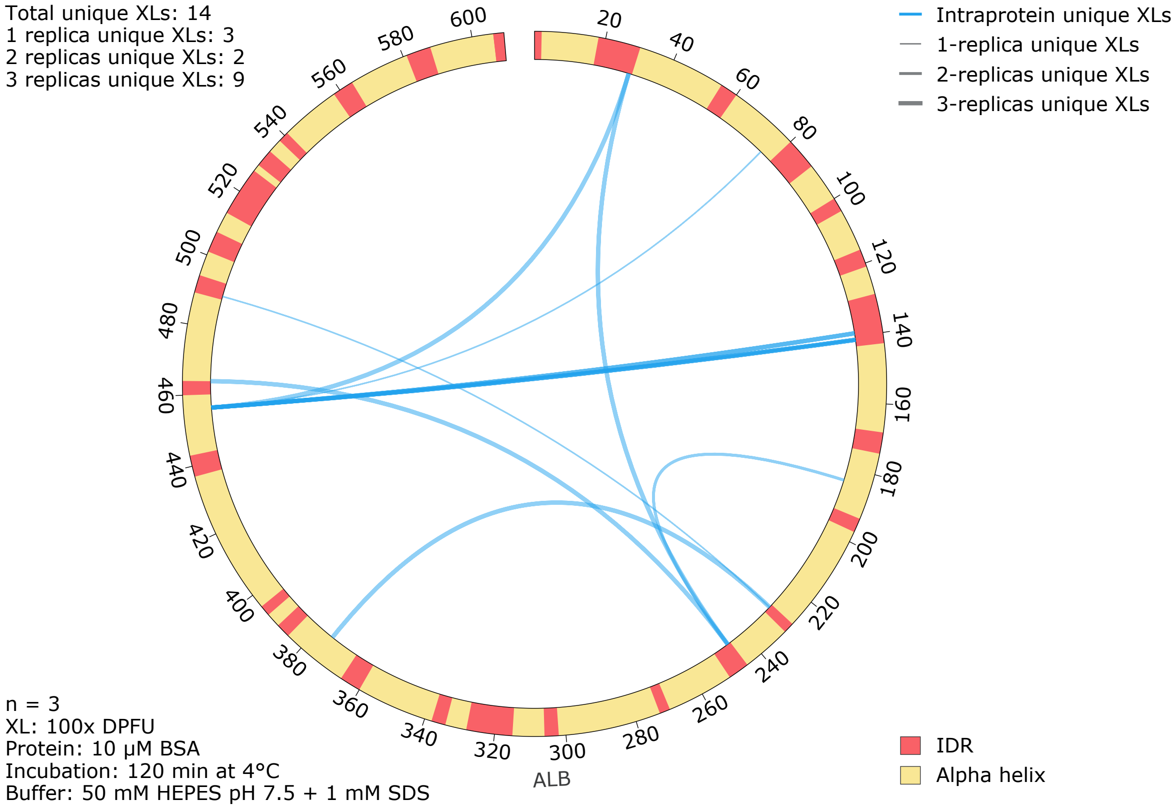

Figure S57. Circos plot of DPFU cross-links (100-fold molar excess) identified for BSA (120 min, 4°C, 1 mM SDS). Cross-links were identified using MeroX. Thickness of lines represents how often a specific cross-link was identified (1/3, 2/3 or 3/3 experiments). Intraprotein cross-links in BSA are shown in sky blue; interprotein (homotypic) cross-links occurred due to dimerization of BSA and are shown in red. In the schematic representation of BSA, α-helices are colored Naples yellow, intrinsically disordered regions (IDRs) are shown in coral red.

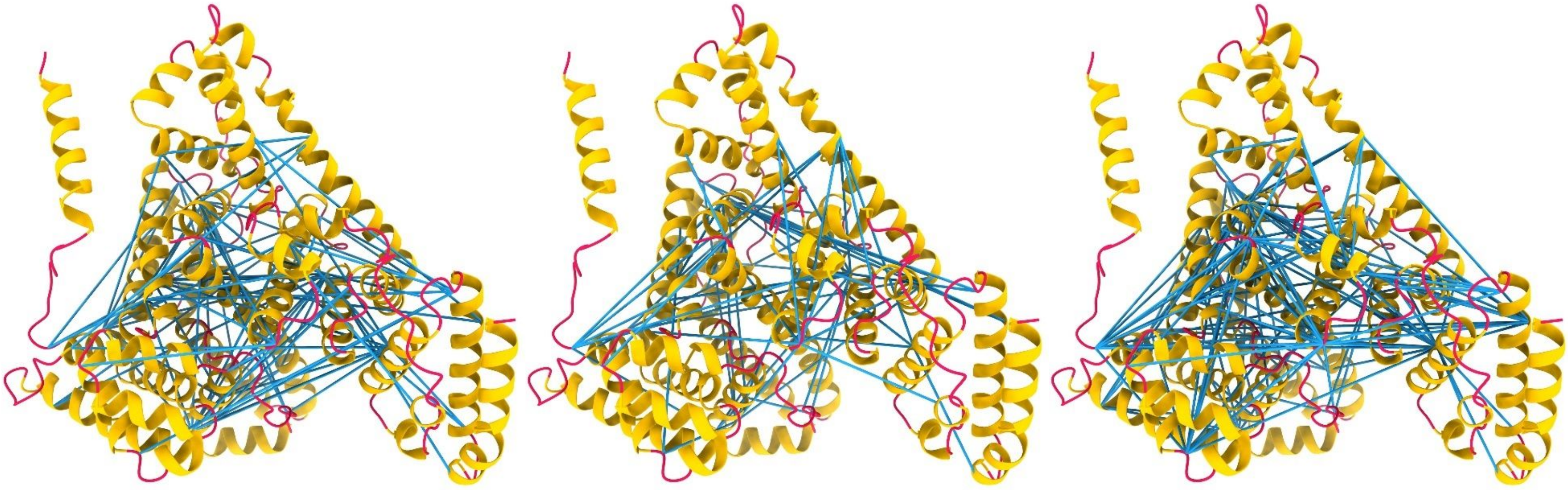

Figure S58. Three-dimensional model of DSBU cross-links (20-fold molar excess) identified for BSA (90 min, room temperature, no SDS). In the cartoon representation of BSA, α-helices are colored Naples yellow, intrinsically disordered regions (IDRs) are shown in coral red, and cross-links are colored sky blue. The numbers of unique cross-links identified in one, two, or three replicates are shown in the left, middle, and right panels. Cross-links are plotted as Cα–Cα pseudobonds between amino acid residues of the BSA AlphaFold model (AF-P02769-F1-v4) using ChimeraX (v1.9).

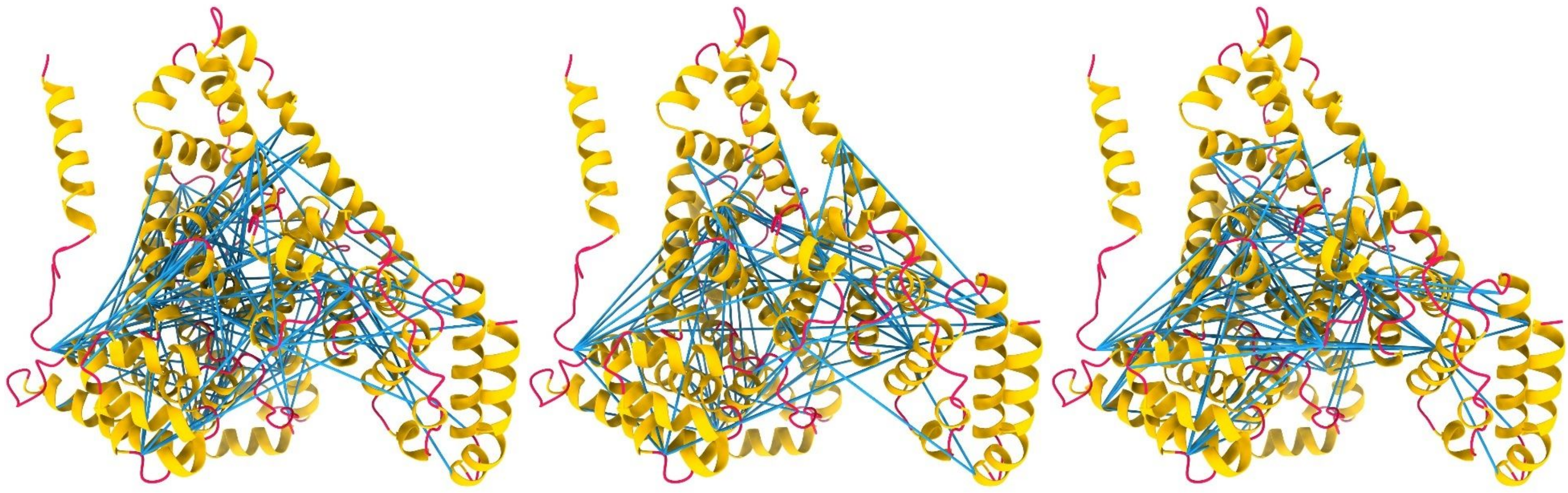

Figure S59. Three-dimensional model of DSBU cross-links (50-fold molar excess) identified for BSA (90 min, room temperature, no SDS). In the cartoon representation of BSA, α-helices are colored Naples yellow, intrinsically disordered regions (IDRs) are shown in coral red, and cross-links are colored sky blue. The numbers of unique cross-links identified in one, two, or three replicates are shown in the left, middle, and right panels. Cross-links are plotted as Cα–Cα pseudobonds between amino acid residues of the BSA AlphaFold model (AF-P02769-F1-v4) using ChimeraX (v1.9).

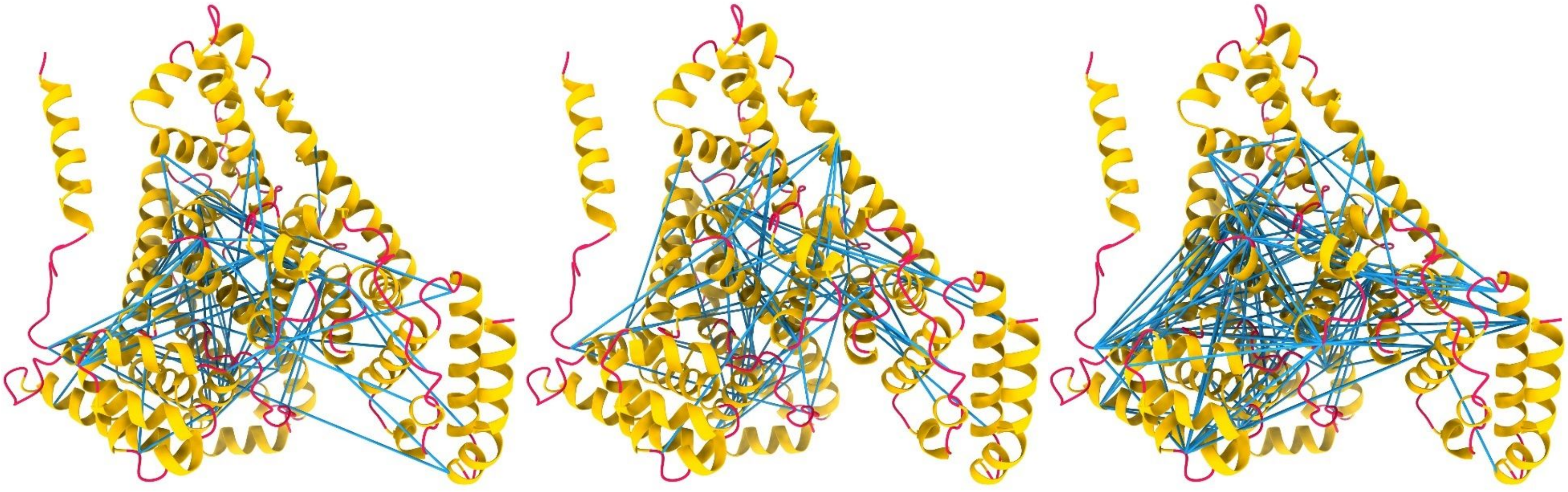

Figure S60. Three-dimensional model of DSBU cross-links (100-fold molar excess) identified for BSA (90 min, room temperature, no SDS). In the cartoon representation of BSA, α-helices are colored Naples yellow, intrinsically disordered regions (IDRs) are shown in coral red, and cross-links are colored sky blue. The numbers of unique cross-links identified in one, two, or three replicates are shown in the left, middle, and right panels. Cross-links are plotted as Cα–Cα pseudobonds between amino acid residues of the BSA AlphaFold model (AF-P02769-F1-v4) using ChimeraX (v1.9).

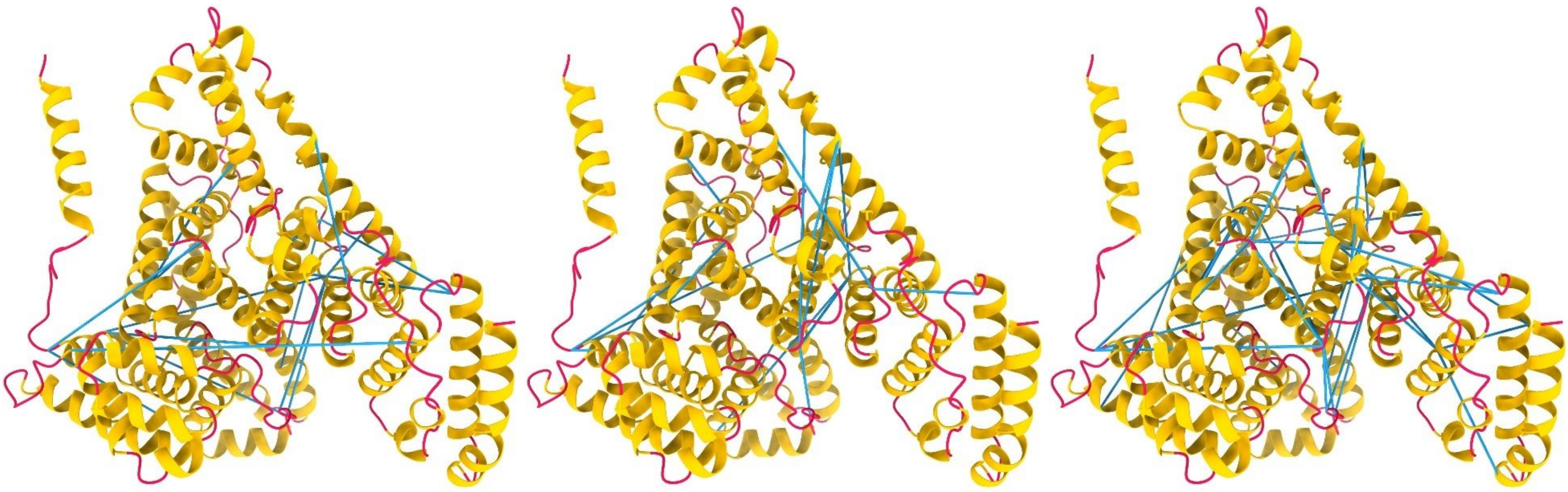

Figure S61. Three-dimensional model of DSBU cross-links (20-fold molar excess) identified for BSA (90 min, room temperature, 1 mM SDS). In the cartoon representation of BSA, α-helices are colored Naples yellow, intrinsically disordered regions (IDRs) are shown in coral red, and cross-links are colored sky blue. The numbers of unique cross-links identified in one, two, or three replicates are shown in the left, middle, and right panels. Cross-links are plotted as Cα–Cα pseudobonds between amino acid residues of the BSA AlphaFold model (AF-P02769-F1-v4) using ChimeraX (v1.9).

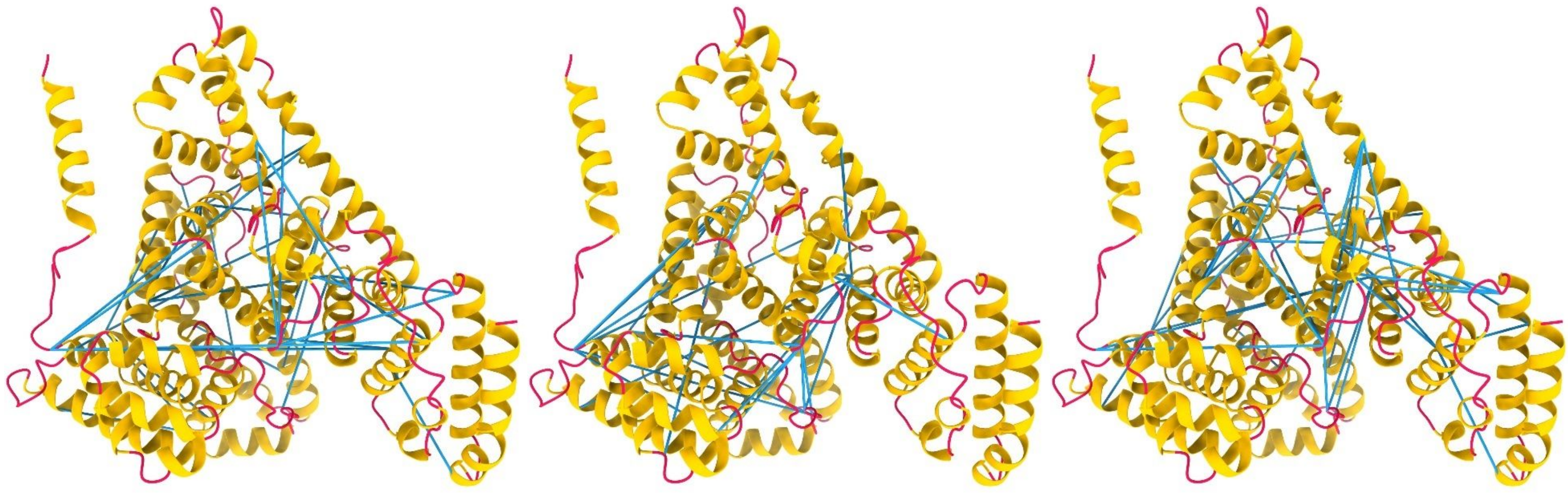

Figure S62. Three-dimensional model of DSBU cross-links (50-fold molar excess) identified for BSA (90 min, room temperature, 1 mM SDS). In the cartoon representation of BSA, α-helices are colored Naples yellow, intrinsically disordered regions (IDRs) are shown in coral red, and cross-links are colored sky blue. The numbers of unique cross-links identified in one, two, or three replicates are shown in the left, middle, and right panels. Cross-links are plotted as Cα–Cα pseudobonds between amino acid residues of the BSA AlphaFold model (AF-P02769-F1-v4) using ChimeraX (v1.9).

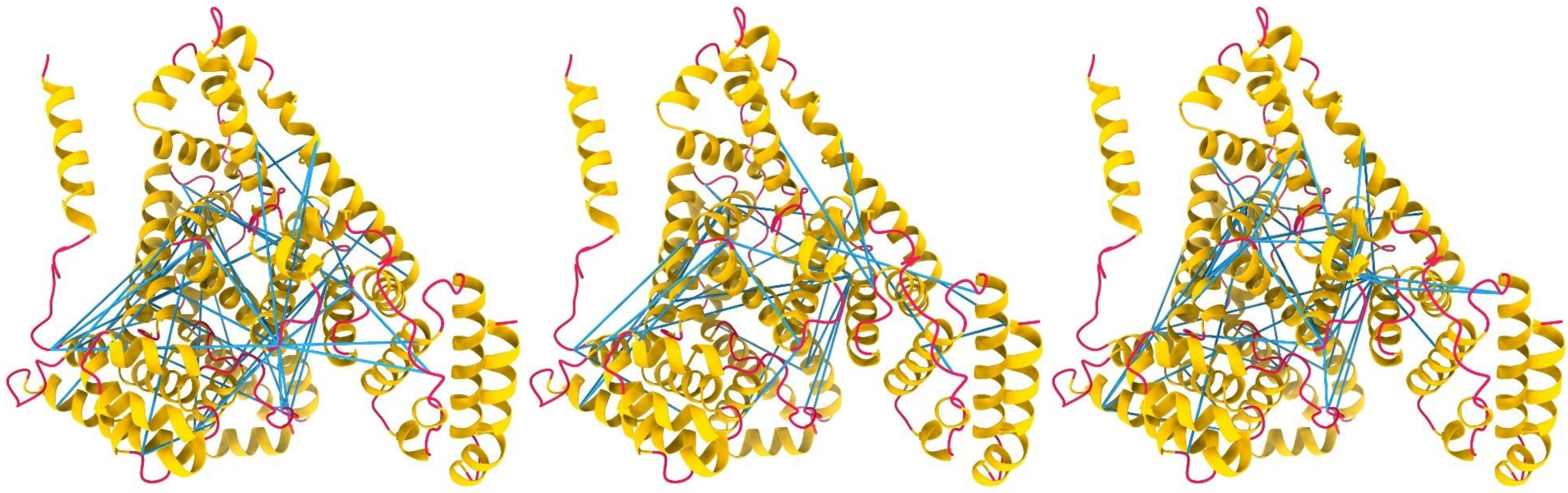

Figure S63. Three-dimensional model of DSBU cross-links (100-fold molar excess) identified for BSA (90 min, room temperature, 1 mM SDS). In the cartoon representation of BSA, α-helices are colored Naples yellow, intrinsically disordered regions (IDRs) are shown in coral red, and cross-links are colored sky blue. The numbers of unique cross-links identified in one, two, or three replicates are shown in the left, middle, and right panels. Cross-links are plotted as Cα–Cα pseudobonds between amino acid residues of the BSA AlphaFold model (AF-P02769-F1-v4) using ChimeraX (v1.9).

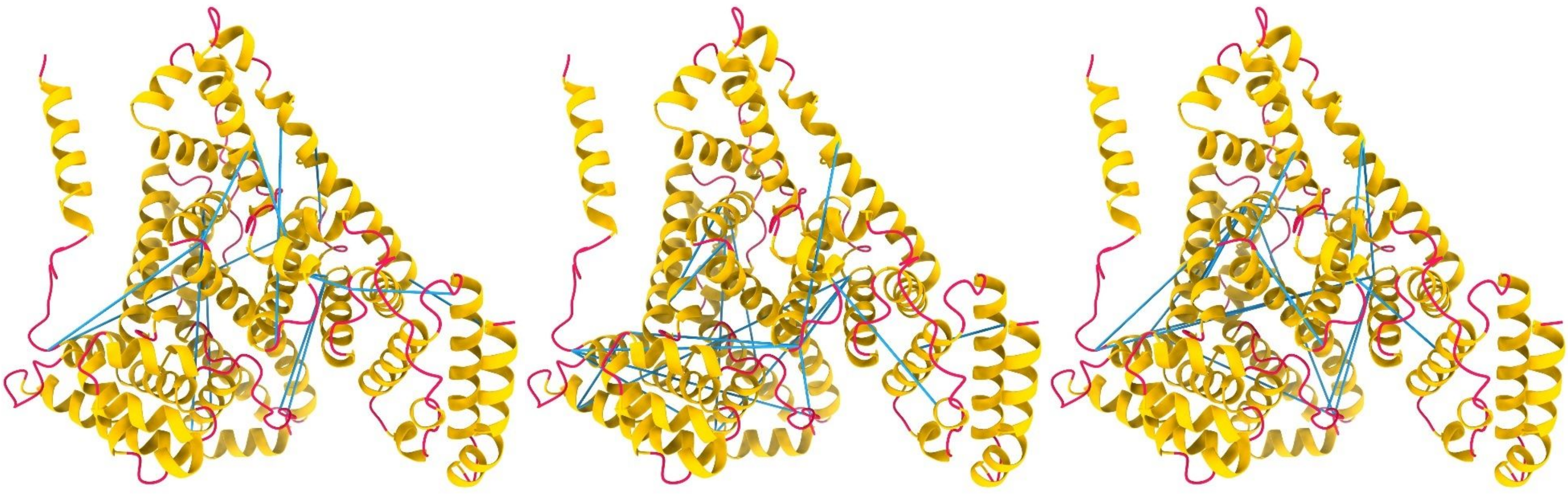

Figure S64. Three-dimensional model of DPFU cross-links (20-fold molar excess) identified for BSA (90 min, room temperature, 1 mM SDS). In the cartoon representation of BSA, α-helices are colored Naples yellow, intrinsically disordered regions (IDRs) are shown in coral red, and cross-links are colored sky blue. The numbers of unique cross-links identified in one, two, or three replicates are shown in the left, middle, and right panels. Cross-links are plotted as Cα–Cα pseudobonds between amino acid residues of the BSA AlphaFold model (AF-P02769-F1-v4) using ChimeraX (v1.9).

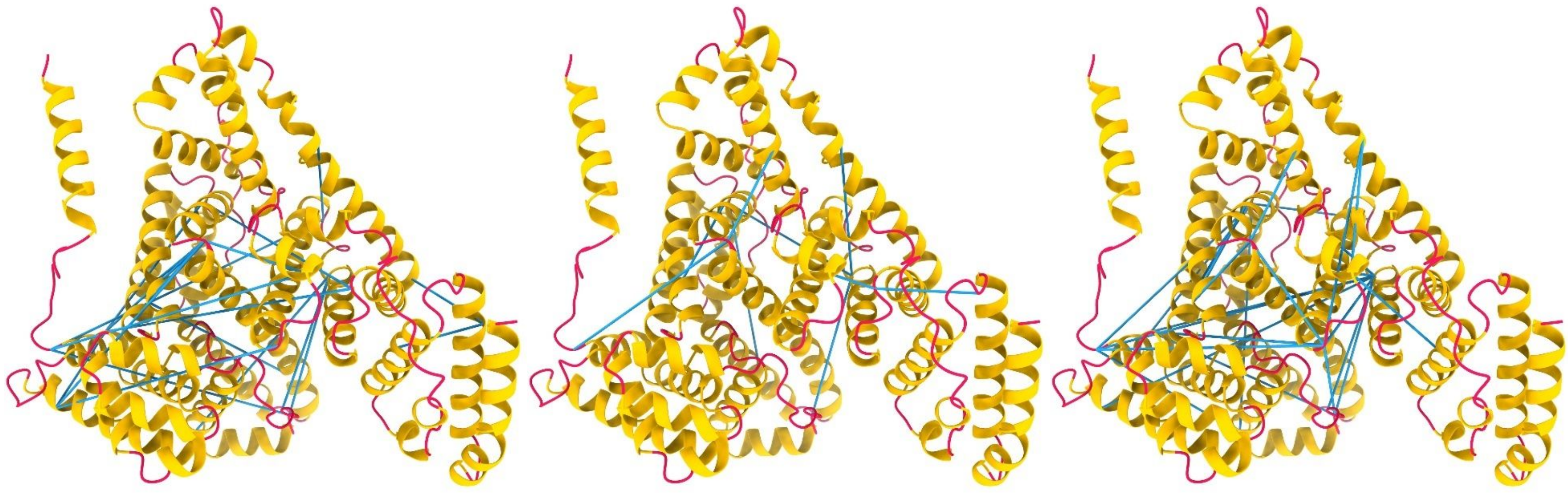

Figure S65. Three-dimensional model of DPFU cross-links (50-fold molar excess) identified for BSA (90 min, room temperature, 1 mM SDS). In the cartoon representation of BSA, α-helices are colored Naples yellow, intrinsically disordered regions (IDRs) are shown in coral red, and cross-links are colored sky blue. The numbers of unique cross-links identified in one, two, or three replicates are shown in the left, middle, and right panels. Cross-links are plotted as Cα–Cα pseudobonds between amino acid residues of the BSA AlphaFold model (AF-P02769-F1-v4) using ChimeraX (v1.9).

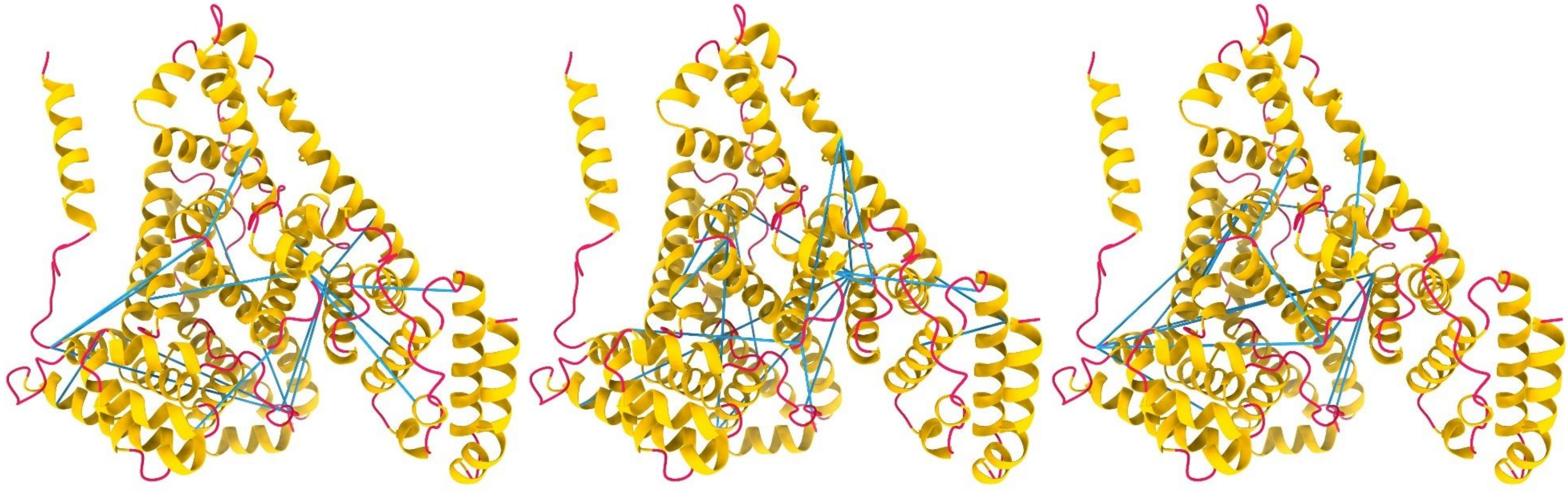

Figure S66. Three-dimensional model of DPFU cross-links (100-fold molar excess) identified for BSA (90 min, room temperature, 1 mM SDS). In the cartoon representation of BSA, α-helices are colored Naples yellow, intrinsically disordered regions (IDRs) are shown in coral red, and cross-links are colored sky blue. The numbers of unique cross-links identified in one, two, or three replicates are shown in the left, middle, and right panels. Cross-links are plotted as Cα–Cα pseudobonds between amino acid residues of the BSA AlphaFold model (AF-P02769-F1-v4) using ChimeraX (v1.9).
